# Chiral Selenium‐Integrated Multi‐Resonant Thermally Activated Delayed Fluorescent Emitters Showing Improved Reverse Intersystem Crossing Rate

**DOI:** 10.1002/anie.202506999

**Published:** 2025-06-22

**Authors:** Jingxiang Wang, Hassan Hafeez, Dongyang Chen, Jhon Sebastian Oviedo Ortiz, Yan Xu, Aidan P. McKay, David B. Cordes, Jeanne Crassous, Ifor D. W. Samuel, Eli Zysman‐Colman

**Affiliations:** ^1^ Organic Semiconductor Centre, EaStCHEM School of Chemistry University of St Andrews St Andrews Fife KY16 9ST UK; ^2^ Organic Semiconductor Centre, SUPA School of Physics and Astronomy University of St Andrews St Andrews KY16 9SS UK; ^3^ University of Rennes, CNRS ISCR (Institut des Sciences Chimiques de Rennes) – UMR 6226 Rennes F‐35000 France

**Keywords:** Aggregation‐caused quenching, Heavy atom effect, Helicene, Multi‐resonant thermally activated delayed fluorescence, Organic light‐emitting diode

## Abstract

Nitrogen/carbonyl (N/C═O) based multi‐resonant thermally activated delayed fluorescence (MR‐TADF) emitters are attractive due to their bright, narrowband emission and the ease with which they can be synthesized. However, their photophysics typically suffer from slow reverse intersystem crossing (RISC) because of their relatively large singlet‐triplet energy gap (Δ*E*_ST_). Thus, the organic light‐emitting diodes (OLEDs) with these emitters typically show severe efficiency roll‐off. Here, two MR‐TADF emitters **DiKTaSe** and **tBuCz‐DiKTaSe** have been designed and synthesized. The introduction of selenium in the form of an annelated benzoselenophene enhances spin‐orbit coupling and increases the RISC rate. The twisted *ortho*‐substituted *tert*‐butylcarbazole moiety in **tBuCz‐DiKTaSe** helps to suppress aggregation‐caused quenching of the emission in films. In addition, the large size of the selenium atom and long C─Se bonds induce helical chirality in both **DiKTaSe** and **tBuCz‐DiKTaSe**. Finally, the OLEDs with **DiKTaSe** showed maximum external quantum efficiency (EQE_max_) of 22.7% while OLEDs with **tBuCz‐DiKTaSe** showed a higher EQE_max_ of 27.8% and less‐pronounced efficiency roll‐off, with EQE at 100 cd m^−2^ (EQE_100_)/ EQE at 1000 cd m^−2^ (EQE_1000_) of 23.5/12.5%. These efficiency values are amongst the highest of devices employing DiKTa‐based emitters. Our work provides key insight into how to judiciously employ heavy atoms to increase the performance of the emitter and the device.

## Graphical abstract

The selenium‐based **tBuCz‐DiKTaSe** emitter containing a twisted *ortho*‐disposed *tert*‐butylcarbazole shows high Φ_PL_, suppressed ACQ, fast *k*_RISC_ and is chiral. The OLEDs show high EQE_max_ and alleviated efficiency roll‐off compared to devices with carbonyl‐based MR‐TADF emitters.

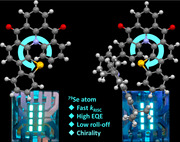

## Introduction

Multi‐resonant thermally activated delayed fluorescence (MR‐TADF) materials have generated considerable interest as emitters in organic light‐emitting diodes (OLEDs). The short‐range charge transfer (SRCT) character for the emissive S_1_ state induced by the *ortho* or *para* localized election‐donating and electron‐withdrawing atoms in the polycyclic aromatic framework is responsible for the TADF in these compounds.^[^ ^1^ ^]^ Their rigid structure endows the MR‐TADF emitter with narrowband emission and high photoluminescence (PL) quantum yields (Φ_PL_). Thus, these attractive properties permit MR‐TADF OLEDs to achieve simultaneously high external quantum efficiency (EQE) and saturated color. Hatakeyama et al. reported the first MR‐TADF emitter, **DABNA‐1**, which contained a boron atom as the electron‐withdrawing group.^[^ ^2^ ^]^ It emits at peak wavelength (λ_PL_) of 460 nm and has a full width at half maximum (FWHM) of 30 nm, a Φ_PL_ of 88% and a singlet‐triplet energy gap (Δ*E*_ST_) of 0.18 eV in 1 wt% doped films in 3,3′‐Di(9*H*‐carbazol‐9‐yl)‐1,1′‐biphenyl (mCBP). The OLEDs with **DABNA‐1** as an emitter exhibited a narrow blue emission at 459 nm (FWHM of 28 nm) and a maximum EQE (EQE_max_) of 13.5%. Since this pioneering report, a large number of boron‐based MR‐TADF emitters have been developed and used in high‐performance OLEDs.^[^ ^3^, ^4^ ^]^ However, the key borylation step frequently requires high temperature and pyrophoric reagents, and the borylation is often not well controlled in terms of the chemoselectivity, resulting in generally moderate yields.^[^ ^5^, ^6^ ^]^

Jiang, Liao and co‐workers^[^ ^7^, ^8^ ^]^ and our group^[^ ^9^ ^]^ reported another category of MR‐TADF emitter (**DiKTa**, also known as **QAO**, **QAD**) where the boron acceptor atoms are replaced with carbonyl‐based moieties. These compounds typically form under mild and high‐yielding conditions, usually via a Friedel‐Crafts acylation to produce the cyclized product.^[^ ^9^ ^]^ **DiKTa** shows a narrowband blue emission at λ_PL_ of 463 nm (FWHM of 37 nm) and has a Φ_PL_ of 75% and Δ*E*_ST_ of 0.20 eV in 3.5 wt% doped films in 1,3‐bis(*N*‐carbazolyl)benzene (mCP). Depending on the device configuration, the OLEDs showed an EQE_max_ of 19.4% at the electroluminescence peak wavelength (λ_EL_) of 468 nm^[^ ^7^ ^]^ or an EQE_max_ of 14.7% at λ_EL_ of 465 nm.^[^ ^9^ ^]^ Although many **DiKTa**‐based MR‐TADF emitters have been reported, including examples of devices showing very high EQE_max_ of >30%, their efficiency roll‐off is severe at high current density due to the slow reverse intersystem crossing (RISC) linked to the relatively large Δ*E*_ST_ (Figure S40, Table S5).^[^ ^8^, ^10^, ^11^, ^12^, ^13^, ^14^, ^15^, ^16^, ^17^, ^18^, ^19^, ^20^, ^21^, ^22^, ^23^, ^24^, ^25^, ^26^, ^27^, ^28^, ^29^, ^30^, ^31^ ^]^

According to Fermi's Golden rule, the rate constant of RISC (*k*_RISC_) can be expressed as Equation 1:^[^ ^32^ ^]^

(1) $$k_{RISC}\propto {\left|H_{SO}\right|}^{2}\;exp\left(-\frac{\Delta E_{ST}}{k_{B}\;T}\right)$$

where *k*_B_, *H_SO_* and *T* refer to Boltzmann's constant, the spin‐orbit coupling (SOC) matrix element, and temperature, respectively. Thus, despite a large Δ*E*_ST_ a fast *k*_RISC_ can be achieved by strengthening the SOC. As the value of SOC is approximately proportional to the fourth power of the nuclear charge of the atoms involved in the emissive transition, heavy atoms can be introduced to the **DiKTa** core to enhance *k*_RISC_.^[^ ^33^, ^34^ ^]^ Jiao et al. reported **JY‐2‐Cl**, a **DiKTa** derivative decorated with six chlorine atoms (Figure S40).^[^ ^35^ ^]^ It exhibits monomer and dimer dual emission at 509 and 545 nm with high Φ_PL_ of 82.6% in 3 wt% doped film in 4,4′‐Bis(*N*‐carbazolyl)‐1,1′‐biphenyl (CBP). Due to the heavy atom effect (HAE) linked to the chlorine atoms, it has much shorter delayed fluorescence lifetimes (τ_d_) of 48.1 and 206.6 µs measured at 480 and 570 nm, respectively, compared to **JY‐2**, a derivative without any chlorine atoms (τ_d_ of 628 µs). The OLEDs with **JY‐2‐Cl** showed high EQE_max_ of 29.1% yet still showed severe efficiency roll‐off, with EQE at 100 cd m^−2^ (EQE_100_) of around 10% and EQE at 1000 cd m^−2^ (EQE_1000_) of around 1%. Yu et al. embedded oxygen, sulfur and selenium atoms into the **DiKTa** (**QAO**) core in the spiro‐locked analogs **SOQ**, **SSQ** and **SSeQ** (Figure S40).^[^ ^6^ ^]^ These three compounds emit at 457, 458 and 456 nm, with narrow FWHMs of 32, 30 and 34 nm, and have high Φ_PL_ values of 95%, 92% and 96%, respectively, in 3 wt% doped film in mCBP. Although they all have comparably moderate Δ*E*_ST_ of 0.21–0.22 eV, the *k*_RISC_ calculated from the time‐resolved PL decays in doped films steadily improved from 2.06 × 10^4^ to 2.69 × 10^4^ and 4.54 × 10^4^ s^−1^ for **SOQ**, **SSQ**, and **SSeQ**, respectively. The **SSQ**‐based device showed the highest EQE_max_ of 25.5% at 456 nm (FWHM of 31 nm) and lowest efficiency roll‐off, with EQE_100_ of 13.4% and EQE_1000_ of 3.5%. Jiang et al. integrated S‐containing spiro structures into the **DiKTa** (**QAO**) unit in **SpiroS‐QAO**, **SpiroSO2‐QAO**, **SpiroO‐QAO** and **SpiroOSO2‐QAO**.^[^ ^36^ ^]^ These compounds emit at 494, 458, 484 and 485 nm, have narrow FWHMs of 43, 32, 34 and 33 nm and moderate Δ*E*_ST_ of 0.21, 0.21, 0.23 and 0.23 eV in toluene. Their doped films in PPF showed high Φ_PL_ values of 94%, 88%, 88% and 89% and *k*_RISC_ of 2.0 × 10^4^, 1.4 × 10^4^, 6.1 × 10^3^ and 4.9 × 10^3^ s^−1^, respectively. Of these four, **SpiroS‐QAO** displayed the highest Φ_PL_ and fastest *k*_RISC_ and the device exhibited the highest EQE_max_ of 27.3%.

We previously reported the brominated DiKTa derivatives, **dBr‐tBu‐DiKTa** and **tBr‐DiKTa**, containing two and three peripheral bromine atoms substituted about the DiKTa core (Figure S40).^[^ ^37^ ^]^ Compared to the reference bromine‐less emitter **Mes_3_‐DiKTa**,^[^ ^9^ ^]^ the *k*_RISC_ values increased progressively from 2.92 × 10^3^ to 1.93 × 10^4^ and 1.97 × 10^5^ s^−1^, while the Φ_PL_ decreased from 90%, 82% to 61% in 1% doped films in mCP as a function of increasing bromine content. The OLED with **dBr‐tBu‐DiKTa** showed the same EQE_max_ of 21% at low luminance but showed decreased efficiency roll‐off compared to the device with **Mes_3_‐DiKTa**. However, due to the poor stability resulting from the weaker C‐Br bonds and the stronger propensity to aggregate of **dBr‐tBu‐DiKTa** in films, the device showed a much worse EQE_max_ than that of the device with **Mes_3_‐DiKTa** at higher luminance. These examples document that it is not simply a case of incorporating heavier atoms to lead to improved device performance but that considerations as to photochemical stability and Φ_PL_ also need to be taken into account in the quest to design higher performance MR‐TADF emitters.

Here, we report two new MR‐TADF emitters, **DiKTaSe** and **tBuCz‐DiKTaSe**. The internal selenium atom was introduced to strengthen the SOC and maintain the stability of the molecules. A twisted *tert*‐butyl carbazole substituent in **tBuCz‐DiKTaSe** acts as a spacer to suppress aggregation. In addition, the large size of the selenium atom and long C‐Se bonds induce the formation of a configurationally stable helicene structure, which endows the emitters with chiroptical properties observable at room temperature. As a result, **DiKTaSe** and **tBuCz‐DiKTaSe** exhibit narrowband blue emission at 464 nm (FWHM of 34 nm) and 470 nm (FWHM of 35 nm) in toluene, respectively. In 2 wt% doped film in 2,6‐bis(3‐(carbazol‐9‐yl)phenyl)pyridine (2,6‐DCzPPy), **DiKTaSe** emits at 484 nm (FWHM of 60 nm), with Φ_PL_ of 62%. By contrast, **tBuCz‐DiKTaSe** shows narrower emission at 489 nm, with a FWHM of 50 nm, and has a higher Φ_PL_ of 82%. The red‐shift and broadening of the PL spectra of the 2 wt% doped films compared to those in toluene can be attributed to the intermolecular interaction between host and guest as well as aggregation of emitters. Thanks to the internal selenium atom, both **DiKTaSe** and **tBuCz‐DiKTaSe** show fast *k*_RISC_ of 9.97 × 10^4^ and 16.5 × 10^4^ s^−1^ (Table 1). The separated enantiomers of **DiKTaSe** and **tBuCz‐DiKTaSe** show symmetric circular dichroism (CD) signals in toluene with absorption dissymmetry factors (|*g*_abs_|) of 1.7 × 10^−3^ and 1.8 × 10^−3^, respectively. Due to their poor Φ_PL_ in toluene, we were unable to detect their CPL signals in solution. However, as a 2 wt% doped film in PMMA, it was possible to measure CPL activity in the case of the enantiomers of **tBuCz‐DiKTaSe**, with an emission dissymmetry factor (|*g*_PL_|) of around 2.3 × 10^−3^. The OLEDs with **DiKTaSe** as an emitter showed an EQE_max_ of 22.7% at λ_EL_ of 480 nm and FWHM of 63 nm. The devices with **tBuCz‐DiKTaSe** showed a higher EQE_max_ of 27.8% at λ_EL_ of 490 nm and a slightly narrower FWHM of 57 nm. In addition, the efficiency roll‐off for the device with **tBuCz‐DiKTaSe** was alleviated as compared to that with **DiKTaSe**, showing EQE_100_ /EQE_1000_ of 23.5%/12.5% (EQE_100_ /EQE_1000_ of 16.7%/7.8% for **DiKTaSe**). The devices with these two emitters showed improved EQE_max_ and reduced efficiency roll‐off compared to the device with **Mes_3_DiKTa** (EQE_max_ /EQE_100_ /EQE_1000_ of 21.1%/14.5%/4.5%), an emitter whose structure mitigates ACQ but does not contain heavy atoms.^[^ ^9^ ^]^

**Table 1:** Photophysical data of **DiKTaSe** and **tBuCz‐DiKTaSe**.

Compound	λ_abs_ ^a)^ (nm)	λ_PL_ ^b)^ (FWHM) (nm)	Δ*E*_ST_ ^c)^ (eV)	λ_PL_ ^d)^ (FWHM) (nm)	Φ_PL_ ^e)^ (%)	τ_p_ ^f)^ (ns)	τ_d_ ^f)^ (µs)	*k*_RISC_ ^f)^ (10^4^ s^—1^)	HOMO/ LUMO^g)^ (eV)
**DiKTaSe**	439	464 (34)	0.22	484 (60)	62	0.4	178	9.97	−5.98/−3.02
**tBuCz‐DiKTaSe**	448	470 (35)	0.22	489 (50)	82	0.8	183	16.5	−5.57/−3.01

^a)^ In toluene.

^b)^ In toluene at room temperature, λ_exc_ = 340 nm.

^c)^ In 2‐MeTHF at 77 K, λ_exc_ = 340 nm, Δ*E*_ST_ = *E*(S_1_) – *E*(T_1_).

^d)^ In 2 wt% doped films in 2,6‐DCzPPy at 300 K under vacuum, λ_exc _= 340 nm.

^e)^ In 2 wt% doped films in 2,6‐DCzPPy measured using an integrating sphere, under N_2_ at λ_exc _= 430 nm.

^f)^ In 2 wt% doped films in 2,6‐DCzPPy at 300 K under vacuum, λ_exc _= 375 nm.

^g)^ In degassed DCM with 0.1 M [*^n^* Bu_4_N]PF_6_ as the supporting electrolyte and Fc/Fc^+^ as the internal reference (0.46 V vs. SCE).^[^ ^45^ ^]^

## Results and Discussion

**Figure 1:**
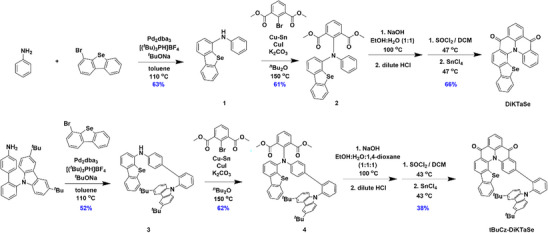
Synthesis of **DiKTaSe** and **tBuCz‐DiKTaSe**.

**Figure 2:**
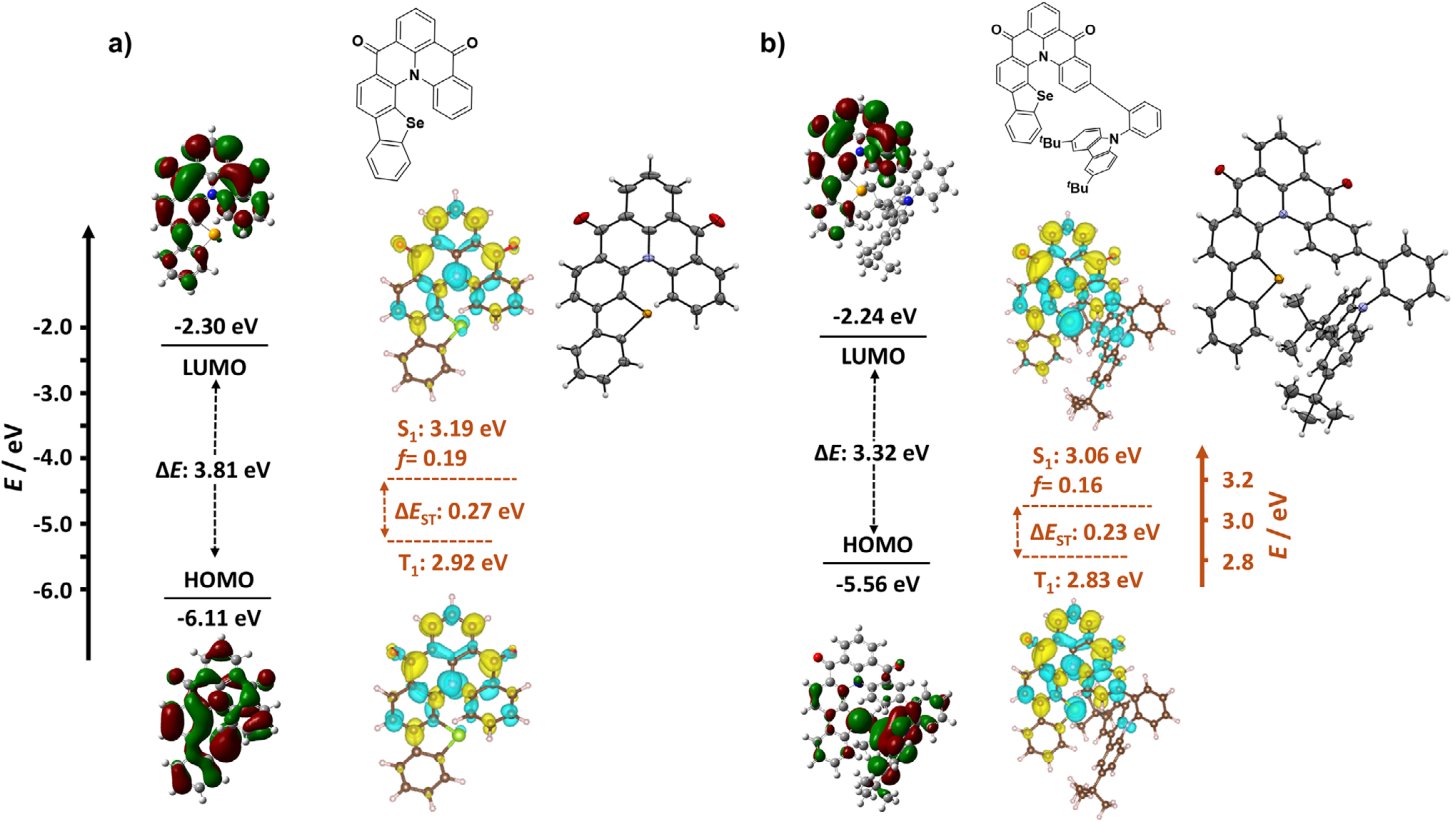
Calculated electron density distribution (isovalue: 0.02) of the HOMO, LUMO and energy levels in the gas phase at the PBE0/6‐31G(d,p) level, difference density plots and energies of S_1_ and T_1_ calculated in the gas phase at SCS‐(ADC)2/cc‐pVDZ level (Blue indicates an area of decreased electron density while yellow indicates increased electronic density between the ground and excited states), and thermal ellipsoid plot of the single crystal structures, respectively, for a) **DiKTaSe** and b) **tBuCz‐DiKTaSe** (ellipsoids are drawn at the 50% probability level and solvent molecules are omitted for clarity).

The syntheses of **DiKTaSe** and **tBuCz‐DiKTaSe** are outlined in Figure 1. The intermediates **1** and **3** were prepared via a palladium‐catalyzed Buchwald–Hartwig amination in 63% and 52% yield, respectively. These intermediates were reacted with 2‐bromo*iso*phthalic acid dimethyl ester under Ullmann coupling conditions to afford **2** and **4** in 61% and 62% yield, respectively, which were then hydrolyzed to their diacids, activated using thionyl chloride and ultimately cyclized under Friedel–Crafts acylation conditions to afford racemic **DiKTaSe** and **tBuCz‐DiKTaSe** in yields of 66% and 38%, respectively. The identity and purity of **DiKTaSe** and **tBuCz‐DiKTaSe** were characterized by melting point determination, ^1^H and ^13^C nuclear magnetic resonance (NMR) spectroscopy, high‐resolution mass spectrometry (HRMS), high‐performance liquid chromatography (HPLC), elemental analysis (Figures S1–S22) and single crystal X‐ray diffraction analysis (Figure 2, Table S1). The enantiomers of **DiKTaSe** and **tBuCz‐DiKTaSe** were separated using chiral HPLC to afford (*P*)‐**DiKTaSe** (*ee*. of 100%), (*M*)‐**DiKTaSe** (*ee*. of 99.8%), (*P*)‐**tBuCz‐DiKTaSe** (*ee* of 100%) and (*M*)‐**tBuCz‐DiKTaSe** (*ee* of 98.2%) (Figures S23, S24). The absolute configurations (*P* and *M*) were tentatively assigned by comparison of the measured and simulated CD spectra in toluene solution (Figure S25). Their thermal stability was assessed by thermogravimetric analysis (TGA) under a nitrogen atmosphere. Both **DiKTaSe** and **tBuCz‐DiKTaSe** have high thermal decomposition temperatures (*T*_d_, corresponding to 5% weight loss) of 372 °C and 448 °C, respectively, which are sufficiently high to permit sublimation of the material without decomposition during the vacuum deposition process of OLED fabrication. No glass transition temperature was observed in the differential scanning calorimetry (DSC) measurement of either compound (Figure S26).

The optimized ground‐state geometries and frontier molecular orbitals of **DiKTaSe** and **tBuCz‐DiKTaSe** were first simulated using density functional theory (DFT) at the PBE0/6‐31G(d,p) level (Figure 2).^[^ ^38^, ^39^ ^]^ The highest occupied molecular orbital (HOMO) and lowest unoccupied molecular orbital (LUMO) of **DiKTaSe** are distributed over the entire polycyclic aromatic system. The HOMO and LUMO energies are calculated to be −6.11/−2.30 eV, with an energy gap (Δ*E*) of 3.81 eV. The slightly shallower HOMO, deeper LUMO and narrower Δ*E* compared to those of the parent compound **DiKTa** (HOMO of −6.20 eV, LUMO of −2.23 eV and Δ*E* of 3.97 eV)^[^ ^16^ ^]^ are attributed to a combination of the introduction of the electron‐donating selenium atom and the extension of π‐conjugation within the framework. The LUMO distribution of **tBuCz‐DiKTaSe** is similar to that of **DiKTaSe** while the HOMO is mainly localized on the *tert*‐butyl carbazole moiety due to its stronger electron‐donating ability, which results in shallower HOMO and similar LUMO energies (−5.56/−2.24 eV) compared to those of **DiKTaSe**.

The excited‐state energies were firstly calculated using time‐dependent DFT (TD‐DFT) within the Tamm‐Dancoff approximation (TDA) at the PBE0/6‐31G(d,p) level (Figure S27). The S_1_ and T_1_ energies are calculated to be 3.21/2.61 and 2.78/2.53 eV for **DiKTaSe** and **tBuCz‐DiKTaSe** and the corresponding Δ*E*_ST_ values are 0.60 and 0.25 eV, respectively, which, not surprisingly, are overestimated given the level of theory employed.^[^ ^40^, ^41^ ^]^ Nonetheless, it is clear that the TADF efficiency of **tBuCz‐DiKTaSe** will be higher than of **DiKTaSe**. The SOC matrix elements (SOCME) were then calculated based on the optimized T_1_ geometry at the PBE0/def2‐TZVP level (Figure S27). There are five low‐lying triplet states below the S_1_ of **DiKTaSe**, which may be implicated in facilitating RISC.^[^ ^42^, ^43^, ^44^ ^]^ The SOCME for S_1_‐T_1_, S_1_‐T_2_, S_1_‐T_3_, S_1_‐T_4_ and S_1_‐T_5_ transitions are 3.22, 13.3, 16.9, 10.8 and 3.97 cm^−1^, respectively, for **DiKTaSe**. By contrast, the S_1_ state of **tBuCz‐DiKTaSe** shows long‐range charge transfer (LRCT) character. There are only two triplet states that lie below the S_1_ state and the SOCME values are 2.31 and 10.2 cm^−1^ for the S_1_‐T_1_ and S_1_‐T_2_ transitions. The larger SOCME values compared to those of **DiKTa** (0.68, 7.29, 9.86 and 7.70 cm^−1^, respectively) can thus be directly attributed to the HAE linked to the presence of selenium. The energies and difference densities of the excited singlet and triplet states were also calculated using Spin‐Component Scaling second‐order algebraic diagrammatic construction (SCS‐(ADC)2/cc‐pVDZ), which we have previously shown to provide very accurate predictions of the state energies in MR‐TADF compounds.^[^ ^40^, ^41^ ^]^ The difference density plots for the transitions to both S_1_ and T_1_ states of **DiKTaSe** and **tBuCz‐DiKTaSe** show the typical alternating increasing and decreasing electron density patterns on the adjacent atoms of the MR‐TADF core transitions, which indicate these states have SRCT character (Figure 2). The S_1_ and T_1_ energies are calculated to be 3.19/2.92 and 3.06/2.83 eV and the corresponding Δ*E*_ST_ values are calculated to be 0.27 and 0.23 eV, respectively. These values are comparable to those of other **DiKTa**‐based MR‐TADF emitters^[^ ^16^, ^26^, ^31^ ^]^ including **DiKTa** (Δ*E*_ST_ = 0.26 eV),^[^ ^26^ ^]^ which indicates that these two compounds are likely to be TADF. The more stabilized S_1_ and T_1_ energies compared to those of **DiKTa** (S_1 _= 3.46 eV and T_1 _= 3.20 eV)^[^ ^26^ ^]^ indicate that their emission should be red‐shifted compared to the parent.

The HOMO and LUMO energy levels of **DiKTaSe** and **tBuCz‐DiKTaSe** were then inferred from cyclic voltammetry (CV) and differential pulse voltammetry (DPV) measurements in degassed DCM, with 0.1 M [*^n^* Bu_4_N]PF_6_ as the supporting electrolyte and Fc/Fc^+^ as the internal reference (0.46 V vs. SCE).^[^ ^45^ ^]^ As shown in Figure S28, the CVs of both **DiKTaSe** and **tBuCz‐DiKTaSe** show reversible reduction waves at almost the same reduction peak potentials (*E*_red_) from the DPV of −1.32 and −1.33 V vs. SCE, values that are close to that of **DiKTa** at −1.34 V.^[^ ^16^ ^]^ The CV of **DiKTaSe** shows an irreversible oxidation wave, which is consistent with the electrochemical behavior of **DiKTa**.^[^ ^16^ ^]^ In line with the computations, the oxidation peak potential (*E*_ox_) of **DiKTaSe** at 1.64 V is cathodically shifted compared to that of **DiKTa** (1.78 V).^[^ ^16^ ^]^ By contrast, the CV of **tBuCz‐DiKTaSe** shows a reversible oxidation wave that is strongly cathodically shifted at *E*_ox_ of 1.23 V, implying oxidation of the *tert*‐butyl carbazole group; indeed, a similar *E*_ox_ of 1.27 V was also observed for **Cz‐Ph‐DiKTa**.^[^ ^16^ ^]^ The corresponding HOMO and LUMO energies of **DiKTaSe** and **tBuCz‐DiKTaSe** are calculated to be −5.98/−3.02 and −5.57/−3.01 eV, leading to Δ*E* of 2.96 and 2.56 eV, respectively (Table 1, Table S2). The trends align well with the computational values.

**Figure 3:**
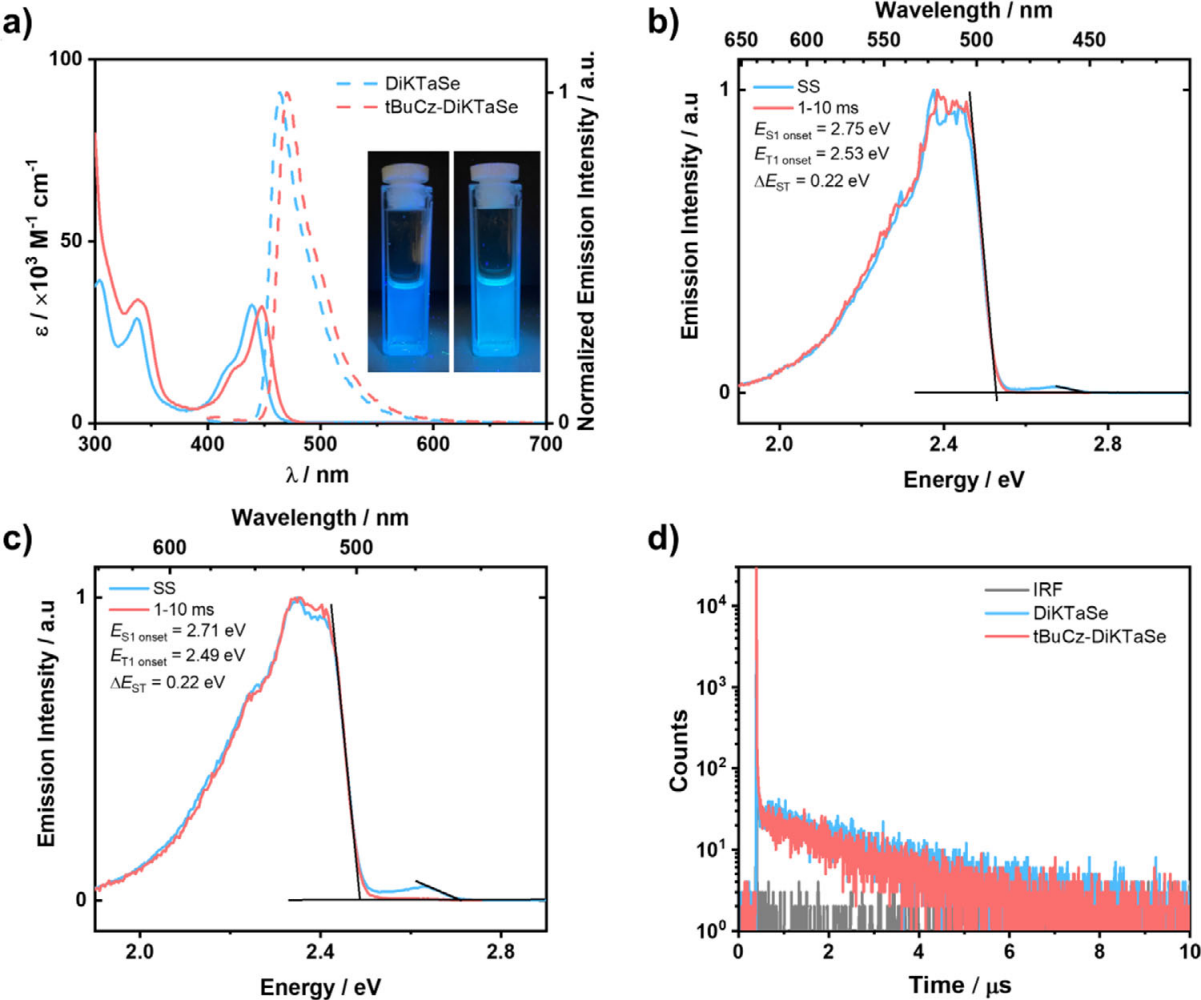
a) UV–vis absorption and PL spectra in toluene at 300K (λ_exc_ = 340 nm); Inset: photos of **DiKTaSe** (left) and **tBuCz‐DiKTaSe** (right) in toluene excited at 365 nm. Steady‐state PL spectra and phosphorescence spectra of b) **DiKTaSe** and c) **tBuCz‐DiKTaSe** in 2‐MeTHF at 77 K (λ_exc_ = 340 nm). d) Time‐resolved PL decays of **DiKTaSe** and **tBuCz‐DiKTaSe** in degassed toluene at 300 K (λ_exc_ = 375 nm).

We next investigated the ultraviolet‐visible (UV‐vis) absorption and PL properties of the two emitters in toluene at room temperature (Figure 3a). The absorption spectra of **DiKTaSe** and **tBuCz‐DiKTaSe** show similar lowest‐energy S_0_‐S_1_ SRCT absorption bands at 439 and 448 nm, which are close to that of **DiKTa** (433 nm).^[^ ^9^ ^]^ The molar absorptivity of these bands is the same at 3.2 × 10^4^ M^−1^ cm^−1^ and larger than that of **DiKTa** (2.1 × 10^4^ M^−1^ cm^−1^).^[^ ^9^ ^]^ The modest bathochromic shifting of the SRCT band of **DiKTaSe** and **tBuCz‐DiKTaSe** compared to that of **DiKTa** is consistent with their smaller Δ*E* values and the trend from the SCS‐(ADC)2 calculations. The PL spectra of both **DiKTaSe** and **tBuCz‐DiKTaSe** show narrowband emission at λ_PL_ of 464 and 470 nm, with FWHMs of 34 nm/0.19 eV and 35 nm/0.19 eV. There are small Stokes shifts of 25 nm/0.15 eV and 22 nm/0.13 eV, respectively, which indicates that there is a small geometry relaxation between ground and excited states. The small degree of positive solvatochromism observed in the PL spectra of **DiKTaSe** confirms the SRCT character of its S_1_ state across the polarity range encompassed by these solvents (Figure S29). By contrast, a new emission band at 630 nm is observed in the PL spectra of **tBuCz‐DiKTaSe** in the high polarity solvent acetone, which can be ascribed to the stabilization of a LRCT state between the *tert*‐butyl carbazole donor and the **DiKTa** core, acting now as the electron‐acceptor. Similar behavior has been reported previously for donor‐substituted **DiKTa** derivatives.^[^ ^16^, ^31^ ^]^

The singlet and triplet energies were determined from the onsets of the steady‐state PL (SS PL) and phosphorescence (Ph) spectra in 2‐MeTHF at 77 K, respectively. As shown in Figure 3b,c, the S_1_ and T_1_ energies of **DiKTaSe** and **tBuCz‐DiKTaSe** are 2.75/2.53 and 2.71/2.49 eV, respectively, resulting in Δ*E*_ST_ values for both of 0.22 eV, which are close to that of **DiKTa** (0.20 eV).^[^ ^9^ ^]^ The time‐resolved PL decays in degassed toluene are shown in Figures 3d and S30. Due to the strong SOC originating from the heavy selenium, the ISC is enhanced and competes with direct fluorescence, making it impossible to determine the prompt fluorescence lifetime in solution for each of the two emitters. Moreover, RISC rates are also accelerated and the delayed lifetimes, τ_d_, are 1.9 and 1.6 µs for **DiKTaSe** and **tBuCz‐DiKTaSe**, respectively, despite their large Δ*E*_ST_ values. This PL decay behavior was not observed in toluene solutions of **DiKTa**.^[^ ^26^ ^]^ Due to competing non‐radiative decay processes, the Φ_PL_ values of **DiKTaSe** and **tBuCz‐DiKTaSe** in degassed toluene are only 1.0% and 1.5%, respectively.

The photophysical properties of the emitters in films were then investigated. The bipolar host material 2,6‐DCzPPy was used because of its high triplet energy (*E*_T_ = 2.71 eV) and capacity to balance charge transport within the layer in OLEDs.^[^ ^46^ ^]^ The highest Φ_PL_ values of 69% and 88% for **DiKTaSe** and **tBuCz‐DiKTaSe** were obtained in 1 wt% doped films in 2,6‐DCzPPy. Increasing the doping concentration to 2, 5 and 10 wt% for the films of both **DiKTaSe** and **tBuCz‐DiKTaSe** results in a modestly red‐shifted emission (from 477 to 494 nm for **DiKTaSe** and from 481 to 496 nm for **tBuCz‐DiKTaSe**) that becomes steadily broader (FWHMs from 58 to 76 nm for **DiKTaSe** and from 51 to 67 nm for **tBuCz‐DiKTaSe**), which is the result of increasing contributions from aggregates (Figure S31). This also leads to a decrease in Φ_PL_ of **DiKTaSe** and **tBuCz‐DiKTaSe** to 62%, 53% and 44% and 82%, 69% and 62%, respectively, for the 2, 5 and 10 wt% doped films (Table S3).

**Figure 4:**
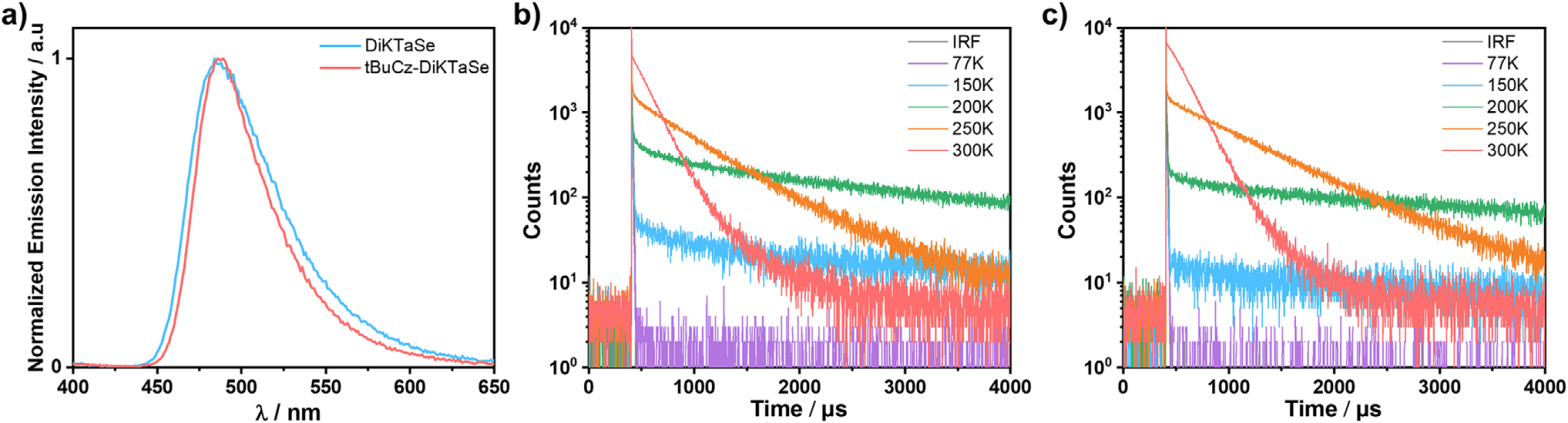
a) Steady‐state PL spectra at 300 K of 2 wt% doped film of **DiKTaSe** and **tBuCz‐DiKTaSe** in 2,6‐DCzPPy (λ_exc_ = 340 nm). Variable temperature time‐resolved PL decays of 2 wt% doped film of b) **DiKTaSe** and c) **tBuCz‐DiKTaSe** in 2,6‐DCzPPy (λ_exc_ = 375 nm).

Steady‐state PL spectra of 2 wt% doped films of **DiKTaSe** and **tBuCz‐DiKTaSe** in 2,6‐DCzPPy peak at λ_PL_ of 484 and 489 nm and have FWHMs of 60 nm/0.30 eV and 50 nm/0.25 eV, respectively (Figure 4a, Table 1). The small bathochromic shifting of the PL spectra compared to that of **DiKTa** (λ_PL_ of 469 nm) is due to the extension of the π‐conjugation within the framework, which aligns well with the calculated Δ*E* values (Figure S32a). The narrower emission band of **tBuCz‐DiKTaSe** is attributed to the introduction of the *tert*‐butyl carbazole, which suppresses the aggregation of the emitters and also conformational motion. The 77 K SS PL and Ph spectra of the doped films indicate that the S_1_/T_1_ energies (Figure S33) are 2.71/2.50 and 2.67/2.48 eV. The Δ*E*_ST_ values for **DiKTaSe** and **tBuCz‐DiKTaSe** are thus 0.21 and 0.19 eV, respectively, slightly smaller than those in toluene. The time‐resolved PL decays under vacuum (Figure 4b,c) reveal prompt fluorescence lifetimes, τ_p_, of 0.4 and 0.8 ns (Figure S33) and τ_d_ of 178 and 183 µs at 300 K, respectively. There is an increase in the delayed emission intensity with increasing temperature that confirms the TADF properties of **DiKTaSe** and **tBuCz‐DiKTaSe**. The calculated photophysical kinetics parameters are summarized in Table S4. The rate constants for ISC (*k*_ISC_) for **DiKTaSe** and **tBuCz‐DiKTaSe** (1.66 × 10^9^ and 0.90 × 10^9^ s^−1^, respectively) are much faster than the radiative decay rate constant from S_1_, *k*_r_^S^ (5.31 × 10^7^ and 2.26 × 10^7^ s^−1^, respectively), which explains the very weak prompt emission in the films. The *k*_RISC_ for **DiKTaSe** and **tBuCz‐DiKTaSe** are 9.97 × 10^4^ and 16.5 × 10^4^ s^−1^, respectively. The larger proportion of delayed emission to the total Φ_PL_ (Table S4) compared to that of **DiKTa** (Figure S32b) is mainly responsible for the improved *k*_RISC_ compared to those of **DiKTa** (*k*_RISC_ of 1.25 × 10^4^ s^−1^) and **SSeQ** (*k*_RISC_ of 4.54 × 10^4^ s^−1^),^[^ ^6^ ^]^ and the similar *k*_RISC_ compared to that of **tBr‐DiKTa** (*k*_RISC_ of 19.7 × 10^4^ s^−1^)^[^ ^37^ ^]^ can all be attributed to the strong HAE from the selenium atom adjacent to the **DiKTa** core.

Time‐resolved PL decays of the films of **DiKTaSe** and **tBuCz‐DiKTaSe** at higher doping concentrations in 2,6‐DCzPPy were also measured. As shown in Figure S34, biexponential delayed decays were observed, with τ_d_ of 94/349 and 79/414 µs for the 5 and 10 wt% doped films of **DiKTaSe**, respectively, and 130/500 and 77/338 µs for 5 and 10 wt% doped films of **tBuCz‐DiKTaSe**, respectively. The longer lifetime component of the delayed emission may be due to the PL decay from aggregates (Figure S31). The *k*_RISC_ values are calculated to be 18.5 × 10^4^ and 13.1 × 10^4^ s^−1^ for **DiKTaSe** and 15.4 × 10^4^ and 13.3 × 10^4^ s^−1^ for **tBuCz‐DiKTaSe** for the 5 and 10 wt% doped films, respectively, which are similar to the *k*_RISC_ values of 9.97 × 10^4^ and 16.5 × 10^4^ s^−1^ for 2 wt% doped films of **DiKTaSe** and **tBuCz‐DiKTaSe**.

**Figure 5:**
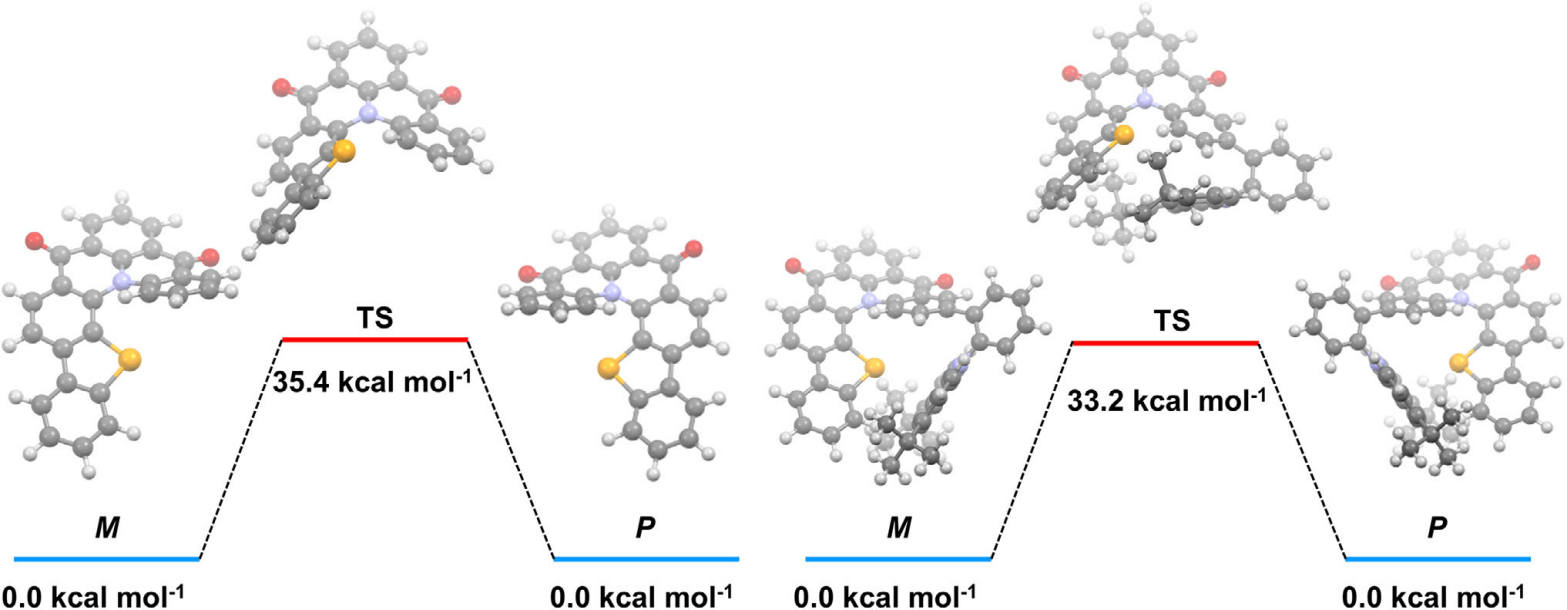
Calculated racemization energy barriers of **DiKTaSe** and **tBuCz‐DiKTaSe** at the M06‐2X/def2‐TZVP level.

To explore the stereochemical stability of **DiKTaSe** and **tBuCz‐DiKTaSe**, the racemization energy barriers of the two helicenes were calculated using DFT at the M06‐2X/def2‐TZVP level of theory (Figure 5). The M06‐2X functional was chosen here because it accurately predicts the SRCT character of the S_1_ state of **tBuCz‐DiKTaSe** instead of the LRCT character predicted by PBE0 (Figure S35). Generally, [n]helicenes (*n* = 5–8) have transition states (TS) (with only one imaginary frequency) belonging to the *C*_s_ point group during racemization.^[^ ^47^ ^]^ As **DiKTaSe** and **tBuCz‐DiKTaSe** have asymmetric structures, the TS cannot strictly satisfy the *C*_s_ symmetry. The large size of Se and relatively long C‐Se bonds contribute to **DiKTaSe** and **tBuCz‐DiKTaSe** having relatively high racemization energy barriers of ΔG^‡^ = 35.4 and 33.2 kcal mol^−1^. The rate constant *k* of a chemical reaction against temperature can be described using the Eyring equation (Equation 2) and the half‐life *t*_1/2_ is defined according to Equation 3:

(2) $$k=\frac{\kappa k_{B}T}{h}e^{-\frac{△G^{‡}}{\mathrm{RT}}}=2\text{.1}\;\times \;10^{\text{10}}Te^{-\frac{\text{1000}△G^{‡}}{1\text{.986T}}}$$

(3) $$t_{1/2}=\frac{ln2}{k}\;$$

Where *κ* is the transmission coefficient (which is often assumed to be equal to one), *k*_B_ is the Boltzmann constant, *T* is the temperature and *h* is the Planck constant. A ΔG^‡^ of 30 kcal mol^−1^ corresponds to a half‐life of more than 35 years at room temperature (298.15 K). Therefore, the calculated high energy barrier to racemization in **DiKTaSe** and **tBuCz‐DiKTaSe** enables the chiral separation of the enantiomers and indicates high stereochemical stability.^[^ ^48^, ^49^, ^50^, ^51^ ^]^

**Figure 6:**
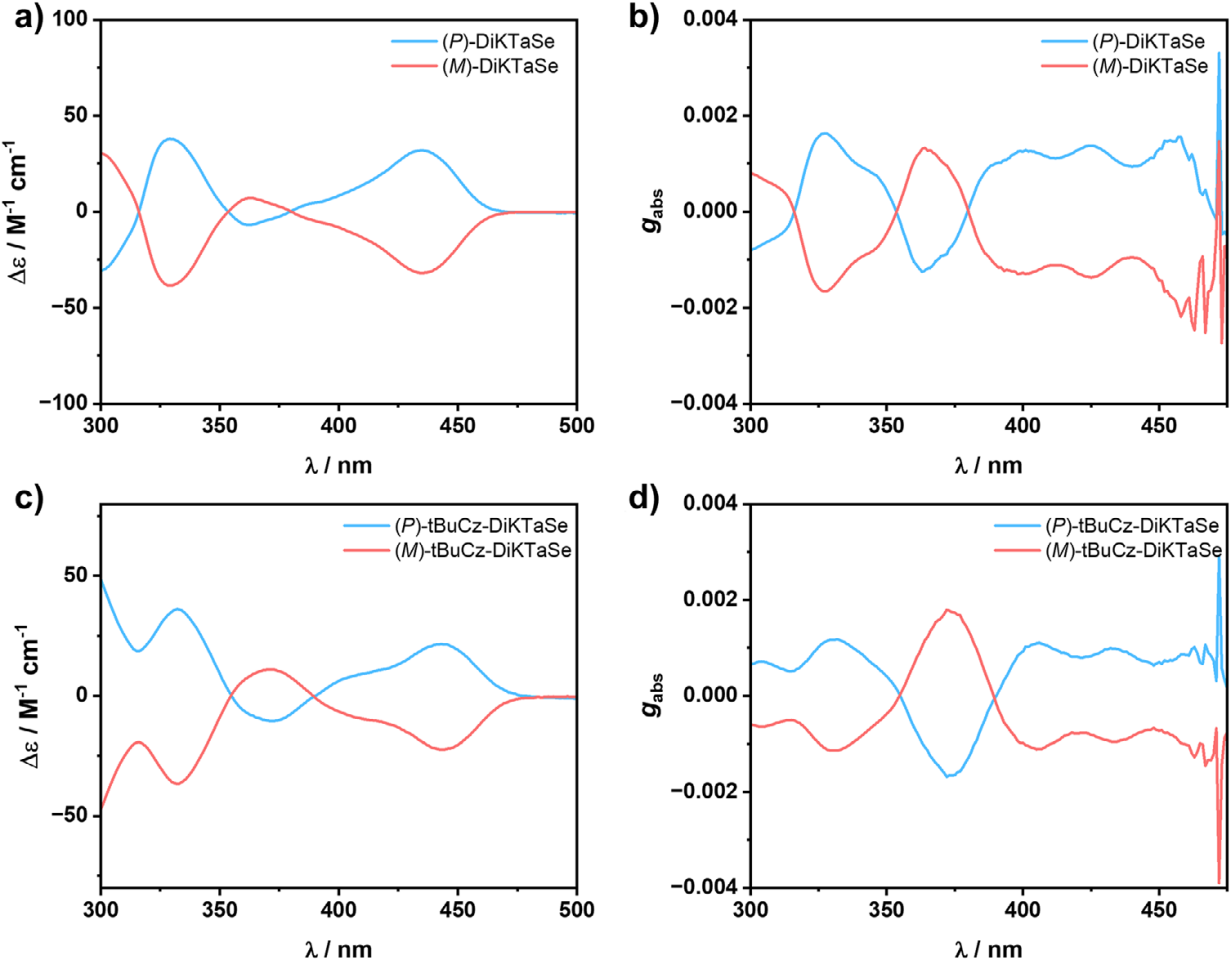
a) CD spectra and b) *g*_abs_ values of **DiKTaSe** recorded in toluene solution. c) CD spectra and d) *g*_abs_ values of **tBuCz‐DiKTaSe** recorded in toluene solution.

The chiroptical properties of both enantiomers of **DiKTaSe** and **tBuCz‐DiKTaSe** were then investigated by CD in toluene. As shown in Figure 6a and c, the CD spectra of both emitters display mirror image Cotton effects. The *g*_abs_ values are + 1.6 × 10^−3^ and ‐1.7 × 10^−3^ at 327 nm for (*P*)/(*M*)‐**DiKTaSe** and ‐1.7 × 10^−3^ and + 1.8 × 10^−3^ at 373 nm for (*P*)/(*M*)‐**tBuCz‐DiKTaSe**. The *g*_abs_ at the lowest‐energy absorption bands (439 and 448 nm for **DiKTaSe** and **tBuCz‐DiKTaSe**, respectively) are 9.5 × 10^−4^/‐9.6 × 10^−4^ for (*P*)/(*M*)‐**DiKTaSe** and 6.3 × 10^−4^/‐6.7 × 10^−4^ for (*P*)/(*M*)‐**tBuCz‐DiKTaSe** (Figure 6b and d). Due to their weak Φ_PL_ in toluene, we were unable to detect the CPL signals in solution (Figure S36). However, it was possible to obtain CPL activity in the case of the 2 wt% doped films of the enantiomers of **tBuCz‐DiKTaSe** in PMMA, with an emission dissymmetry factor |*g*_PL_| of around 2.3 × 10^−3^ (Figure S37). Unfortunately, we did not detect a mirror image CPL signal for **DiKTaSe** because of its weak emission and some anisotropy effects resulting from some aggregation in the solid‐state sample.

## Organic Light‐Emitting Diodes

**Figure 7:**
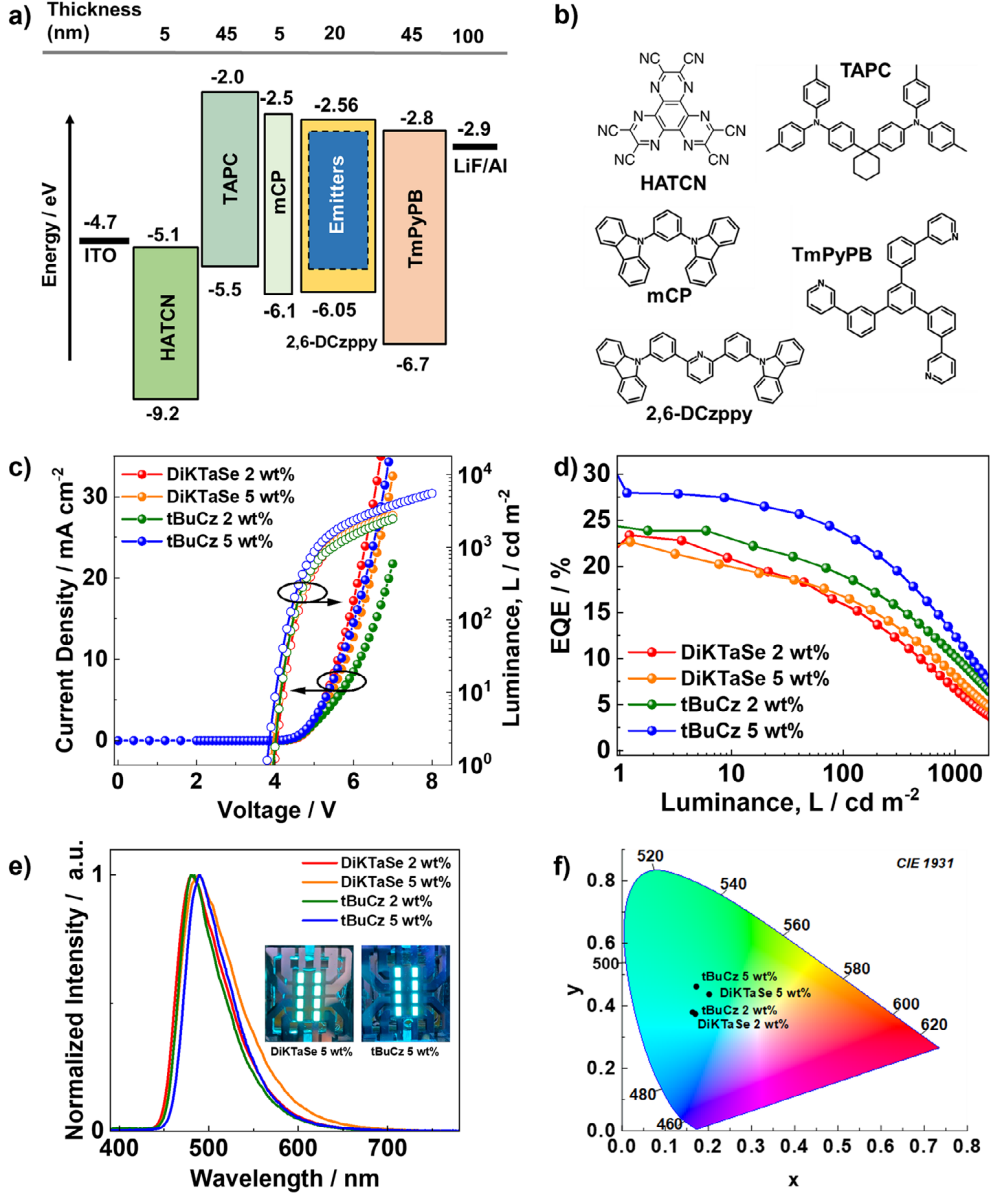
a) Schematic of the OLED stack; b) Chemical structures of the materials used in the device; c) Current density‐voltage‐luminance (*JVL*) characteristics; d) EQE versus luminance; e) Electroluminescence spectra, inset figures are photographs of the OLEDs fabricated with 5 wt% of **DiKTaSe** and **tBuCz DiKTaSe**; f) CIE coordinates of the devices.

Based on their attractive photophysical properties, we next fabricated vacuum‐deposited OLEDs. The device stack and the chemical structures of the organic layers are shown in Figure 7a and b, respectively. The OLED structure consisted of: indium tin oxide (ITO)/1,4,5,8,9,11‐hexaazatriphenylenehexacarbonitrile (HATCN, 5 nm)/1,1‐bis[(di‐4‐tolylamino)phenyl]cyclohexane (TAPC, 45 nm)/mCP (5 nm)/2,6‐DCzPPy as host (20 nm) with **DiKTaSe** and **tBuCz‐DiKTaSe** as emitter dopants/ 1,3,5‐tris(3‐pyridyl‐3‐phenyl)benzene (TmPyPB, 45 nm)/lithium fluoride (LiF, 1 nm)/aluminum (Al, 100 nm). Here, HATCN was used as the hole injection layer, TAPC as the hole transport layer, mCP as an exciton blocking layer, TmPyPB as electron transport layer, and LiF to reduce the work function of the top Al electrode. The OLEDs were fabricated with 2 and 5 wt% of the emitter concentrations. Figure 7c shows the current density‐voltage‐luminance characteristics of the OLEDs where all devices presented a turn‐on voltage of 3.8–4 V.

The devices with 2 and 5 wt% of **DiKTaSe** showed similar efficiency trends, with EQE_max_ of 22.7% and 21.4%, respectively (Figure 7d and Table 2). The EQE_100_ (at 100 cd m^−2^) for the 2 wt% OLEDs was 15.8%, which dropped to 6.5% at 1000 cd m^−2^. Despite the fast *k*_RISC_ of the emitter, significant efficiency roll‐off remains. This is because *k*_ISC_ (Table S4) is also large, and efficiency roll‐off is determined by a combination of *k*_RISC_, *k*_ISC_ and *k*_r_^S^.^[^ ^52^ ^]^ The electroluminescence maximum (λ_EL_) for the 2 wt% device is 480 nm with a FWHM of 63 nm (Figure 7e) and these properties are similar to those of the thin films (Table 1). The Commission Internationale de l'Éclairage chromaticity coordinates, CIE(x, y), for the 2 wt% OLED are (0.169, 0.373), indicating an emission in the sky‐blue region of the spectrum (Figure 7f).

**Table 2:** Device performance for OLEDs with **DiKTaSe** and **tBuCz‐DiKTaSe**.

	x (wt%)	λ_EL_ (nm)	FWHM (nm)	V_on_ (V)	EQE_max/100/1000_ (%)	CIE / x, y
**DiKTaSe**	2	480	63	4.0	22.7/15.8/6.5	0.169, 0.373
	5	489	70	3.9	21.4/16.7/7.8	0.201, 0.436
**tBuCz‐DiKTaSe**	2	480	55	3.9	23.8/19/10.2	0.163, 0.379
	5	490	57	3.8	27.8/23.5/12.5	0.172, 0.461

**Figure 8:**
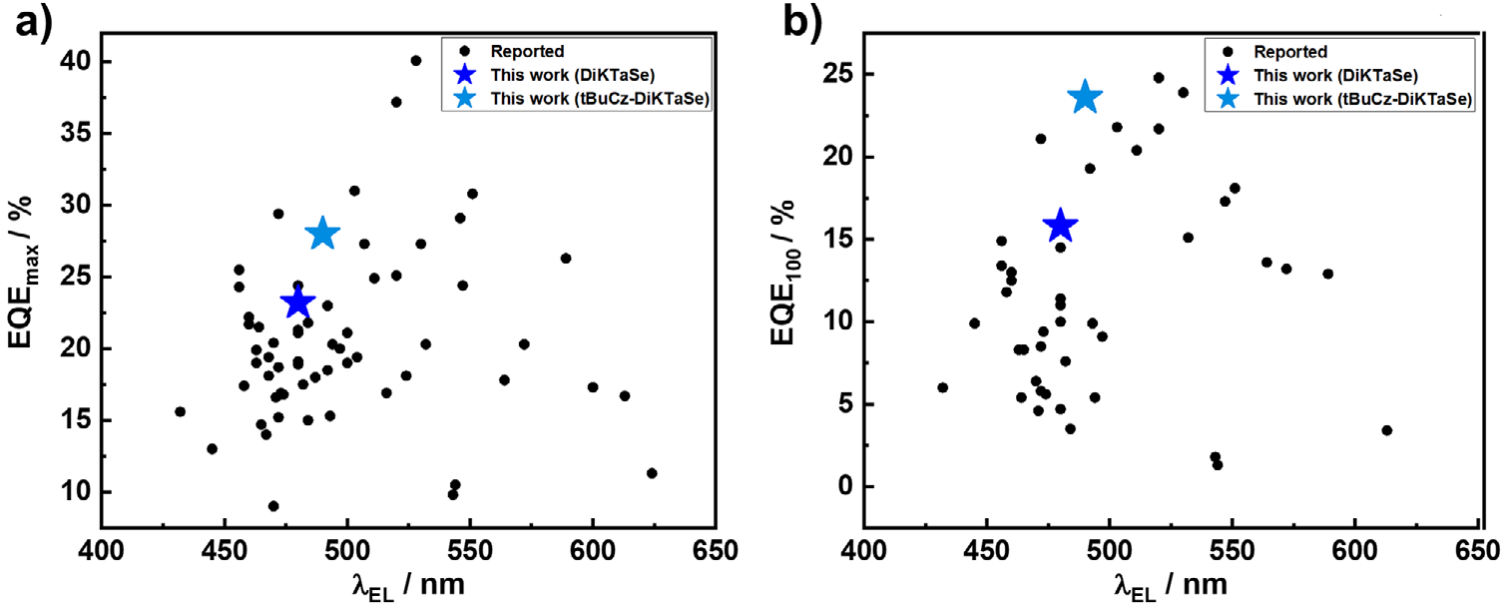
a) EQE_max_ vs λ_EL_ comparison and b) EQE_100_ vs λ_EL_ comparison for reported MR‐TADF DiKTa‐based OLEDs versus devices with **DiKTaSe** and **tBuCz‐DiKTaSe** (detailed chemical structures and device data are provided in Figure S40 and Table S5, respectively).

The **tBuCz‐DiKTaSe** OLEDs showed a slightly higher EQE_max_ of 23.8% using 2 wt% of the emitter, compared to OLEDs with **DiKTaSe**. The efficiency roll‐off of the devices with **tBuCz‐DiKTaSe** was less‐pronounced with an EQE_100_ of 19% and an EQE_1000_ of 10.2% (Figure 7d and Table 2). This higher efficiency of the **tBuCz‐DiKTaSe** OLEDs compared to the ones with **DiKTaSe** is attributed to the higher Φ_PL_ (82% vs. 62%) of the former. The 2 wt% **tBuCz‐DiKTaSe** OLEDs showed an λ_EL_ at 480 nm with a narrower FWHM of 55 nm as compared to that of the device with **DiKTaSe**; and the CIE coordinates for the devices with **tBuCz‐DiKTaS** are (0.163, 0.379). Higher efficiencies at all luminances were observed with an increase in concentration of **tBuCz‐DiKTaSe** in the EML of the OLED to 5 wt%, with EQE_max_/EQE_100_/EQE_1000_ of 27.8%, 23.5% and 12.5%. The devices emitted at λ_EL_ of 490 nm (FWHM of 57 nm), with corresponding CIE coordinates of (0.172, 0.461). Figure 8a and b evidence that the OLEDs with 5 wt% of **tBuCz‐DiKTaSe** showed an outstanding performance that is amongst the highest EQE_max_ and EQE_100_ compared to reported **DiKTa**‐based MR‐TADF OLEDs. This improved performance is attributed to a combination of suppressed aggregation due to the presence of the *ortho*‐substituted *tert*‐butylcarbazole and improved *k*_RISC_ arising from the HAE. The increase in efficiency for the 5 wt% OLEDs occurs in spite of the lower Φ_PL_ of the **tBuCz‐DiKTaSe** emitter for 5 wt% doped film compared to 2 wt% doped film (69% vs. 82%, respectively. This suggests improved charge balance could arise from charge injection directly onto the emitter, and possibly charge transport between emitter molecules, which is supported by the higher current density of the 5 wt% devices (Figure 7c). The barrier to hole injection into the **tBuCz‐DiKTaSe** emitter (HOMO = ‐5.57 eV) is much smaller than injection into the host (HOMO = ‐6.05 eV). OLEDs with higher concentrations (7.5, 10 and 15 wt%) were also fabricated (Figure S38), which all showed slightly lower EQE values and broader EL compared to the 5 wt% doped device. A preliminary assessment of the operational stability of the OLEDs with Se‐based emitters (**DiKTaSe** and **tBuCz‐DiKTaSe**) was compared with **DiKTa** devices at 100 cd m^−2^ as shown in Figure S39. The results show that although lifetimes are short, the addition of Se into the emitter design not only increases the efficiency but also improves the operational stability of the devices.

## Conclusions

Two new selenium‐containing MR‐TADF emitters **DiKTaSe** and **tBuCz‐DiKTaSe** have been designed and synthesized. The strong HAE provided by the selenium accelerates RISC with *k*_RISC_ reaching 9.97 × 10^4^ and 16.5 × 10^4^ s^−1^ in 2 wt% doped film in 2,6‐DCzPPy, respectively. The presence of the twisted *ortho*‐substituted *tert*‐butylcarbazole suppresses emitter aggregation in films of **tBuCz‐DiKTaSe**, leading to higher Φ_PL_ value of 82% and narrower emission in 2 wt% doped film in 2,6‐DCzPPy compared to those of **DiKTaSe**. The OLEDs with **DiKTaSe** showed EQE_max_ of 22.7% at λ_EL_ of 480 nm (FWHM of 63 nm), while the devices with **tBuCz‐DiKTaSe** showed higher EQE_max_ of 27.8% at λ_EL_ of 490 nm and a slightly narrower FWHM of 57 nm. In addition to high EQE_max_, the efficiency roll‐off of the devices with **tBuCz‐DiKTaSe** was less‐pronounced as compared to the devices with both **DiKTaSe** and other reported **DiKTa**‐based OLEDs, showing an impressive EQE_100_ /EQE_1000_ of 23.5%/12.5%. The higher efficiencies for the OLEDs with **tBuCz‐DiKTaSe** attest to the superior emitter design.

## Supporting Information

^1^H and ^13^C NMR spectra, HRMS, HPLC and EA of all target compounds; supplementary computational data, photophysical data and devices data; the structures and properties of all the chiral MR‐TADF emitters.

## Author Contributions

J.W. designed the emitters, performed part of the synthesis, carried out the theoretical and optoelectronics characterization. H.H. fabricated the OLEDs. D.C. performed part of the synthesis. J.S.O.O. measured the chiroptical properties. Y.X. performed some of the separation of enantiomers. A.P.M. and D.B.C carried out the single crystal characterization. J.C. supervised J.S.O.O., I.D.W.S. supervised H.H., and E.Z.‐C. supervised J.W., D.C. and Y.X. All authors contributed to the writing and editing of the manuscript.

## Conflict of Interests

The authors declare no conflict of interest.

## Supporting information



Supporting Information

## Data Availability

The research data supporting this publication can be accessed at https://doi.org/10.17630/4d3f056f‐bf62‐4b92‐bb0f‐629db8997a67.
