# Supplementary material for: Chiral Selenium‐Integrated Multi‐Resonant Thermally Activated Delayed Fluorescent Emitters Showing Improved Reverse Intersystem Crossing Rate
Source: Angew Chem Int Ed Engl. 2025 Jun 22;64(33):e202506999. doi: 10.1002/anie.202506999 (PMC12338389; doi:10.1002/anie.202506999)
Supplement: Supplementary file 1 — Supporting Information [file ANIE-64-e202506999-s001.pdf]

Chiral Selenium-integrated Multi-resonant Thermally Activated Delayed Fluorescent Emitters Showing Improved Reverse Intersystem Crossing Rate

Jingxiang Wang[a‡], Hassan Hafeez[b‡], Dongyang Chen[a], Jhon Sebastian Oviedo Ortiz[c], Yan Xu[a], Aidan P. McKay[a], David B. Cordes[a], Jeanne Crassous[c], Ifor D. W. Samuel[b] and Eli Zysman-Colman[a*]*

[a] Organic Semiconductor Centre, EaStCHEM School of Chemistry, University of St Andrews, St Andrews, Fife, UK, KY16 9ST, Fax: +44-1334 463808; Tel: +44-1334 463826; E-mail: eli.zysman-colman@st-andrews.ac.uk.

[b] Organic Semiconductor Centre, SUPA School of Physics and Astronomy, University of St Andrews, St Andrews, UK, KY16 9SS. Email: idws@st-andrews.ac.uk.

[c] University of Rennes, CNRS, ISCR (Institut des Sciences Chimiques de Rennes) – UMR 6226, F-35000 Rennes, France. E-mail: jeanne.crassous@univ-rennes.fr.

[‡] These authors contributed equally to this work.

Table of Contents

General information **S2**

Synthesis **S7**

Photophysical characterization and computations **S34**

Devices **S41**

Literature study **S43**

References **S47**

General information:

General synthetic information.

All reagents and solvents for the synthesis and characterization were obtained from commercial sources. Anhydrous dichloromethane and toluene were obtained from an MBraun SPS5 solvent purification system. Other chemicals were used directly without additional purification. Air-sensitive reactions are conducted under a nitrogen atmosphere using Schlenk techniques. Flash column chromatography was carried out using silica gel (Silia-P from Silicycle, 60 Å, 40-63 µm). Analytical thin-layer-chromatography (TLC) was performed with silica plates with plastic backings (250 µm with F-254 indicator). TLC visualization was accomplished by 365 nm UV lamp. Melting points were measured using open-ended capillaries on an Electrothermal 1101D Mel-Temp apparatus and are uncorrected. HPLC analysis was conducted on a Shimadzu LC-40 HPLC system. HPLC traces were performed using a Shim-pack GIST 3µm C18 reverse phase analytical column. ^{1}H and ^{13}C NMR spectra were measured using a Bruker AVII 400 and Bruker AVIII-HD 500 NMR spectrometers. The following abbreviations have been used for multiplicity assignments: "s" for singlet, "d" for doublet, "t" for triplet, "dd" for doublet of doublets, "td" for triplet of doublets, "ddd" for doublet of doublets of doublets, "ddt" for doublet of doublets of triplets and "m" for multiplet. Electrospray ionization (ESI) high-resolution mass spectrometry (HRMS) was obtained at the University of Edinburgh Mass Spectrometry Facility. Elemental analyses were performed at London Metropolitan University. TGA/DSC analyses were performed on a Netzsch STA449C instrument under nitrogen gas at a 10 K/min heating rate. Enantiomers were isolated using a Chiralpak IF column (10 mm I.D. x 250 mm) with DCM: Hexane= 95: 5 for **DiKTaSe** and 100% DCM for **tBuCz-DiKTaSe** as the mobile phase, a 2 ml min^{-1} flowrate and UV monitoring at 280 nm. Subsequent analytical cHPLC revealed high enantiopurity (*ee.* 100% for (*P*)-**DiKTaSe**, 99.8% for (*M*)-**DiKTaSe**, 100% for (*P*)-**tBuCz-DiKTaSe** and 98.2% for (*M*)-**tBuCz-DiKTaSe**) (Figure **S23-S24**).

Theoretical Calculations.

All ground-state optimizations were carried out using Density Functional Theory (DFT) level with Gaussian 16[1] using the PBE0[2] functional and the 6-31G(d,p) basis set[3], starting from a structure drawn and optimized using Chem3D. The optimized geometry in T_1 states, natural transition orbitals of excited states were calculated using Time-Dependent DFT (TD-DFT) within the Tamm-Dancoff approximation (TDA)[4] at the PBE0/6-31G(d,p) and PBE0/def2-TZVP levels of theory. The simulated CD spectra were calculated at the M06-2X/def2-TZVP level of theory in toluene. The transition state of the chiral molecules was calculated at the M06-2X/def2-TZVP level. Frequency calculations were performed to verify that the structure is a transition state as there was only one imaginary frequency. Molecular orbitals were visualized using GaussView 6.0 software.[5] Vertical excited states were also calculated using Spin-Component Scaling second-order algebraic diagrammatic construction (SCS-ADC2)/cc-pVDZ calculations based on the ground-state optimized structure using DFT method.[6] Difference density plots were used to visualize change in electronic density between the ground and excited state and were visualized using the VESTA package.[7] Calculations were submitted and processed using Silico V3,[8] which incorporates a number of publicly available software libraries, including: cclib[9] for parsing of result files, VMD[10]/Tachyon[11] for 3D rendering, Matplotlib[12] for drawing of graphs, Open Babel[13]/Pybel[14] for file interconversion and PySOC[15] for the calculation of spin-orbit coupling.

Electrochemistry measurements.

Cyclic Voltammetry (CV) analysis was performed on an Electrochemical Analyzer potentiostat model 620E from CH Instruments at a sweep rate of 100 mV/s. Differential pulse voltammetry (DPV) was conducted with an increment potential of 0.01 V and a pulse amplitude, width, and period of 50 mV, 0.06, and 0.5 s, respectively. All measurements were performed in degassed DCM with 0.1 M tetra-n-butylammonium hexafluorophosphate ($[^{n}Bu_4N]PF_6$) as the supporting electrolyte and ferrocene/ferrocenium (Fc/Fc^+) as the internal reference (0.46 V vs SCE).[16] An Ag/Ag^+ electrode, a glassy carbon electrode and a platinum electrode were used as the reference electrode, working electrode and counter electrode, respectively. The HOMO and LUMO

energies were determined using the relation the relation HOMO/LUMO = -(E_{ox}/ E_{red} vs Fc/Fc^+ +4.8),[17] where E_{ox} and E_{red} are the oxidation and reduction peak potentials versus Fc/Fc^+, respectively, calculated from the DPV.

Photophysical measurements:

Optically dilute solutions of concentrations on the order of 10^{-5} M were prepared in HPLC grade solvent for absorption and emission analysis. Absorption spectra were recorded at room temperature on a Shimadzu UV-2600 double beam spectrophotometer. Molar absorptivity determination was verified by linear regression analysis of values obtained from five independent solutions at varying concentrations from 2×10^{-6} to 2×10^{-5} M. For emission studies, steady-state photoluminescence (PL) spectra and time-resolved PL decays in solution were recorded at 298 K using Edinburgh Instruments FS5 fluorophotometer. Degassed solutions were prepared via three freeze-pump-thaw cycles and spectra were taken using home-made Schlenk quartz cuvettes. Samples were excited at 340 nm for steady-state measurements. Time-resolved PL measurements of solutions were carried out using the time-correlated single-photon counting (TCSPC) technique. The samples were excited at 375 nm by a pulsed laser diode. Photoluminescence quantum yields for solutions were determined using the optically dilute method[18], in which four sample solutions with absorbances between 0.3 to 0.01 at 350 nm were used. The Beer-Lambert law was found to remain linear at the concentrations of the solutions. For each sample, linearity between absorption and emission intensity was verified through linear regression analysis with the Pearson regression factor (R^2) for the linear fit of the data set surpassing 0.9. Individual relative quantum yield values were calculated for each solution and the values reported represent the slope obtained from the linear fit of these results. The quantum yield of the sample, Φ_{PL}, can be determined by the equation $\Phi_{PL} = \left(\Phi_r * \frac{A_r}{A_s} * \frac{I_s}{I_r} * \frac{n_s^2}{n_r^2}\right)$, where A stands for the absorbance at the excitation wavelength (λ_{exc}: 350 nm), I is the integrated area under the corrected emission curve and n is the refractive index of the solvent with the subscripts "s" and "r" representing sample and reference respectively.[19] Φ_r is the absolute quantum yield of the external reference quinine sulfate (Φ_r = 54.6% in 0.5 M

H_2SO_4).[20] The experimental uncertainty in the emission quantum yields is conservatively estimated to be 10%, though we have found that statistically we can reproduce FPL values to 3% relative error.

Thin doped films of emitters in a host matrix were spin-coated on a quartz substrate (for quantum yield measurements) or a sapphire substrate (for time-resolved PL measurements) using a spin speed of 1500 rpm for 60 s to give a thickness of ~80 nm. An integrating sphere (Edinburgh Instruments FS5, SC30 module) was employed for quantum yield measurements for thin film samples. The Φ_{PL} of the films were measured in air and then in N_2 by purging the integrating sphere with N_2 gas flow for 2 min. The photophysical properties of the film samples were measured using an Edinburgh Instruments FS5 fluorimeter. Time-resolved PL measurements of the thin films were carried out using the multi-channel scaling (MCS) and TCSPC technique. The samples were excited at 375 nm by a pulsed laser diode or a xenon flashlamp and were kept in a vacuum of $< 8 \times 10^{-4}$ mbar. The singlet-triplet splitting energy ΔE_{ST} of solutions and films were estimated from the onset of the steady state PL spectrum and phosphorescence spectrum at 77 K. Samples were excited by a xenon flashlamp emitting at 340 nm (EI FS5, SC-70). Phosphorescence spectra were measured with a time-gated window of 1-10 ms.

Electronic circular dichroism (ECD) and circularly polarized luminescence (CPL)

ECD spectra of 10^{-4} M solutions of **DiKTaSe** and **tBuCz-DiKTaSe** in toluene were measured using a Jasco J-815 instrument. CPL measurements were performed using a home-built CPL spectrofluoropolarimeter (constructed with the help of the JASCO Company). The samples were excited using a 90° geometry with a 150 W LS Xenon ozone-free lamp. The concentrations of the samples were measured precisely at ca. 10^{-5} M in toluene. Samples were excited at 410 nm.

Fitting of time-resolved luminescence measurements:

Time-resolved PL measurements were fitted to a sum of exponentials decay model, with chi-squared (χ^2) values between 1 and 2, using the EI FS5 software. Each component of the decay is assigned a weight, (w_i), which is the contribution of the emission from each component to the total emission.

The average lifetime was then calculated using the following:

- Two exponential decay model:

$$\tau_{AVG} = \tau_1 w_1 + \tau_2 w_2$$

with weights defined as $w_1 = \frac{A1\tau_1}{A1\tau_1 + A2\tau_2}$ and $w_2 = \frac{A2\tau_2}{A1\tau_1 + A2\tau_2}$ where A1 and A2 are the preexponential-factors of each component.

- Three exponential decay model:

$$\tau_{AVG} = \tau_1 w_1 + \tau_2 w_2 + \tau_3 w_3$$

with weights defined as $w_1 = \frac{A1\tau_1}{A1\tau_1 + A2\tau_2 + A3\tau_3}$, $w_2 = \frac{A2\tau_2}{A1\tau_1 + A2\tau_2 + A3\tau_3}$ and $w_3 = \frac{A3\tau_3}{A1\tau_1 + A2\tau_2 + A3\tau_3}$ where A1, A2 and A3 are the preexponential-factors of each component.

OLED Fabrication and Characterization:

The bottom-emitting OLEDs were fabricated using pre-patterned indium tin oxide (ITO) on a glass substrate. The substrate was cleaned with acetone then isopropanol for 30 min each, using an ultrasonication bath set at 60 °C. The OLEDs were fabricated using a thermal evaporator (Angstrom Engineering, USA) under a high vacuum with a base pressure of around 3×10^{-7} mbar. The substrate was loaded into the thermal evaporator using a custom-made air-tight metal container to avoid dust particles and contamination. All organic layers were deposited on the ITO anode with evaporation rates between 0.3-0.6 Å/s. This was followed by lithium fluoride (LiF) and aluminium (Al) deposition at the rates of 0.05 Å/s and 2 Å/s, respectively. The

fabricated OLEDs were encapsulated in an N_2 atmosphere using glass that were cleaned using the same method mentioned above for the substrate. A UV-curable epoxy resin (NOA 68) was used to ensure an air-tight attachment of the encapsulation with the substrate. The active (emission) area of the OLEDs was 2 mm^2. The fabricated OLEDs were measured for their current-voltage-luminance characteristics using a Keithley 2400 power source meter and a custom-made photodiode system connected to a Keithley 2000 multimeter. The electroluminescence spectra of the devices were analyzed using a CCD (Andor DV420-BV) spectrometer.

Synthesis:

***N*-phenyldibenzo[*b*,*d*]selenophen-4-amine (1)**

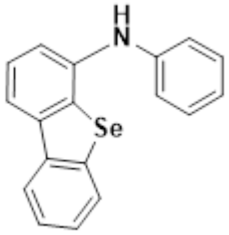

4-bromodibenzo[*b*,*d*]selenophene (600 mg, 1.94 mmol, 1.0 equiv.), aniline (210 µL, 2.32 mmol, 1.2 equiv.), $Pd_2(dba)_3$ (53.2 mg, 0.058 mmol, 0.03 equiv.), tri-tert-butylphosphonium tetrafluoroborate (44.9 mg, 0.155 mmol, 0.08 equiv.), NaO*t*-Bu (558 mg, 5.81 mmol, 3 equiv.) and toluene (15 mL) were added to a 2-neck Schlenk tube. The mixture was stirred at 105 °C for 24 h under a nitrogen atmosphere. After cooling to room temperature, the reaction was quenched by adding water and extracted with 3× 50 mL dichloromethane. The organic phase was then separated and concentrated under reduced pressure. The crude product was purified by column chromatography on silica gel (dichloromethane: hexane= 1: 8) to afford compound **1** as a white solid. **Yield** 63%, 394 mg. **Mp**: 142-143 °C. **R_f**: 0.25 (dichloromethane: hexane= 1: 2). **^{1}H NMR (500 MHz, DMSO-*d*^{6})** δ 8.30 (d, *J* = 7.8 Hz, 1H), 8.14 – 8.05 (m, 2H), 7.98 (d, *J* = 7.7 Hz, 1H), 7.54 – 7.39 (m, 3H), 7.29 – 7.19 (m, 3H), 7.02 – 6.95 (m, 2H), 6.85 (td, *J* = 7.4, 1.4 Hz, 1H). **^{13}C NMR (126 MHz, DMSO-*d*^{6})** δ 144.17, 141.07, 139.89, 139.07, 138.93,

132.60, 129.49, 127.55, 126.79, 126.73, 125.49, 123.81, 120.31, 117.91, 117.64, 117.46.

HRMS (ESI-MS): $[C_{18}H_{13}NSe +H]^+$ **Calculated**: 324.0286; **Found:** 324.0275.

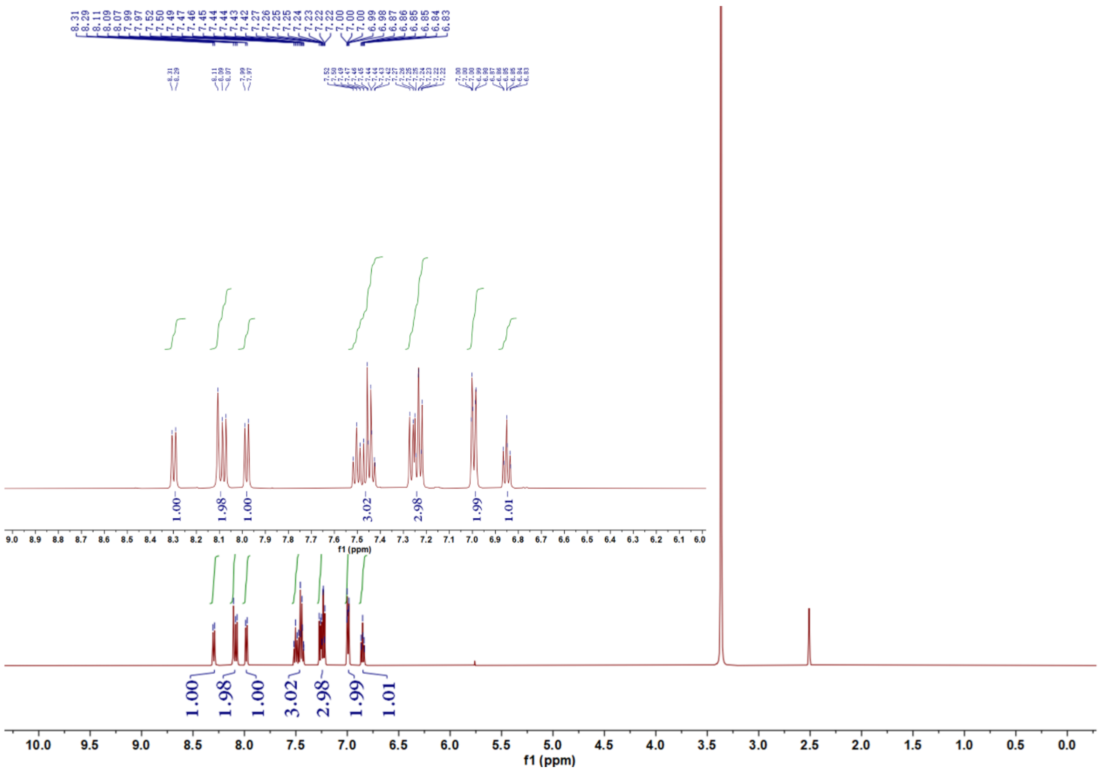

Figure **S1**. ^{1}H NMR spectrum of **1** in DMSO-d^6.

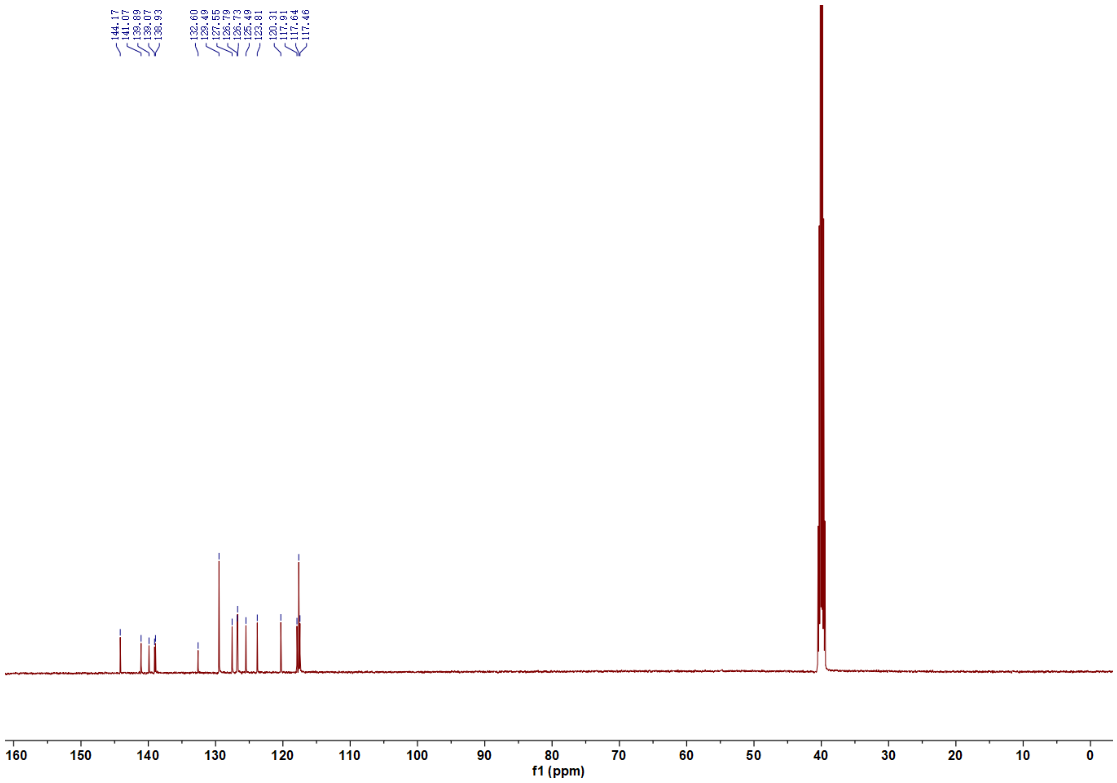

Figure **S2**. ^{13}C NMR spectrum of **1** in DMSO-d^6.

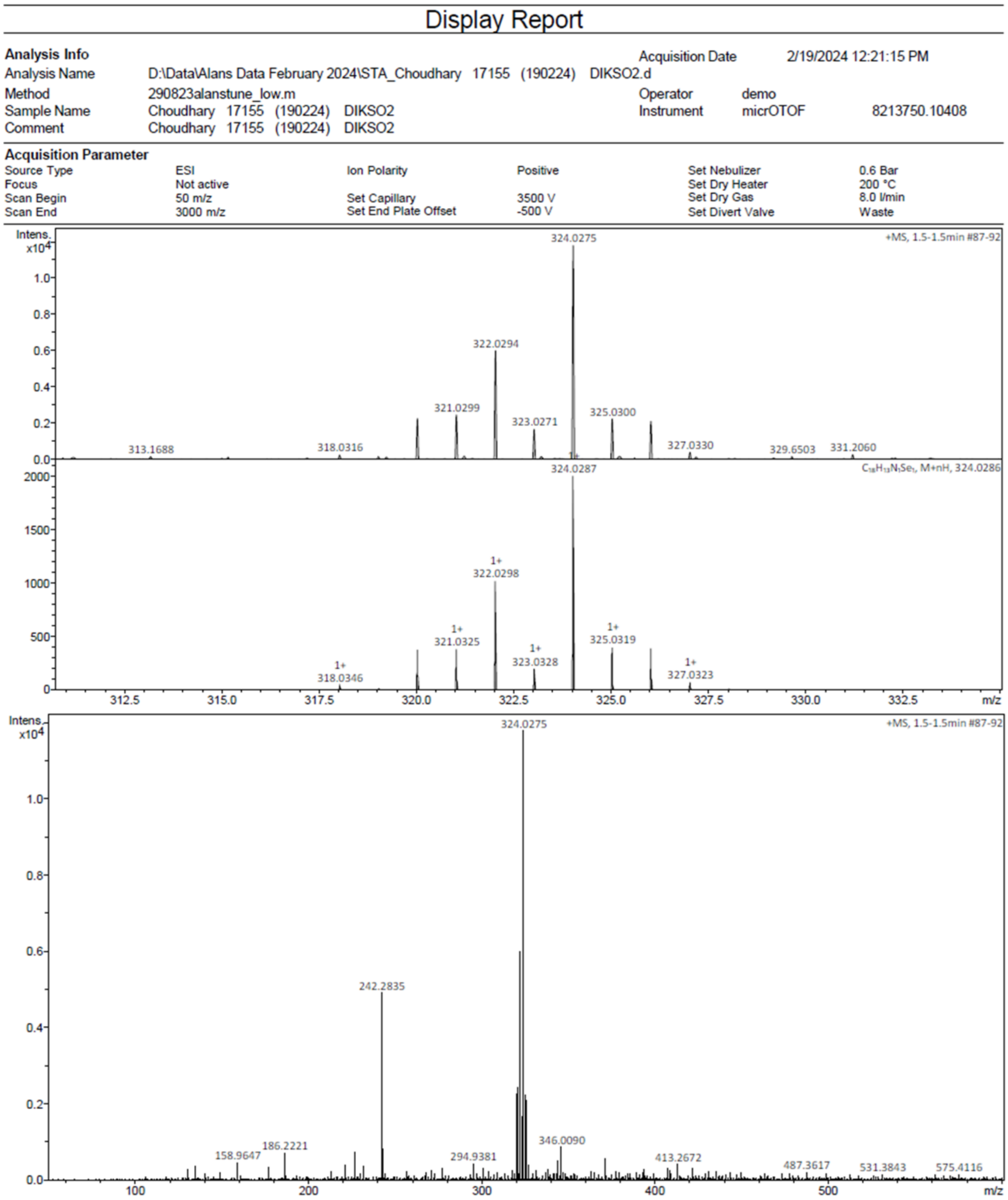

Figure **S3**. HRMS spectrum of **1**.

Dimethyl 2-(dibenzo[*b*,*d*]selenophen-4-yl(phenyl)amino)isophthalate (2)

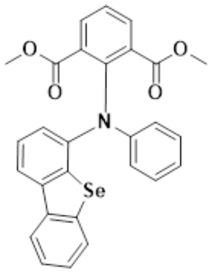

Compound **1** (350 mg, 1.09 mmol, 1.0 equiv.), dimethyl 2-bromoisophthalate (534 mg, 1.96 mmol, 1.8 equiv.), potassium carbonate (450 mg, 3.26 mmol, 3.0 equiv.), copper(I) iodide (20.7 mg, 0.109 mmol, 0.1 equiv.), 2,2,6,6-tetramethyl-3,5-heptanedione (22.6 µL, 0.109 mmol, 0.1 equiv.), copper tin alloy (19.8 mg, 0.109 mmol, 0.1 equiv.) and anhydrous di-*n*-butyl ether (10 mL) were added to a 2-neck Schlenk tube. The resulting reaction mixture was heated to 150 °C under nitrogen and stirred for 3 days. After cooling to room temperature, the reaction was quenched by adding water and extracted with 3× 50 mL dichloromethane. The organic phase was then separated and concentrated under reduced pressure. The crude product was purified by column chromatography on silica gel (ethyl acetate: hexane= 1: 15) to afford compound **2** as a yellowish powder. **Yield** 61%, 341 mg. **Mp**: 203-204 °C. **R_f**: 0.35 (ethyl acetate: hexane= 1: 4). **^{1}H NMR (400 MHz, DMSO-*d*^{6})** δ 8.31 – 8.26 (m, 1H), 8.04 (dd, *J* = 7.9, 1.1 Hz, 1H), 7.94 (ddd, *J* = 7.8, 1.2, 0.6 Hz, 1H), 7.74 (d, *J* = 7.7 Hz, 2H), 7.49 – 7.35 (m, 4H), 7.30 – 7.24 (m, 2H), 7.08 – 7.02 (m, 1H), 6.96 (dd, *J* = 7.8, 1.0 Hz, 1H), 6.87 – 6.81 (m, 2H), 3.25 (s, 6H). **^{13}C NMR (101 MHz, DMSO-*d*^{6})** δ 167.24, 145.12, 143.81, 142.64, 140.25, 140.07, 137.80, 133.56, 132.75, 132.63, 129.21, 127.55, 126.30, 126.16, 125.29, 123.91, 123.72, 123.46, 118.58, 52.48. **HRMS (ESI-MS)**: $[C_{28}H_{21}NO_4Se + H]^+$ **Calculated**: 516.0709; **Found:** 516.0718.

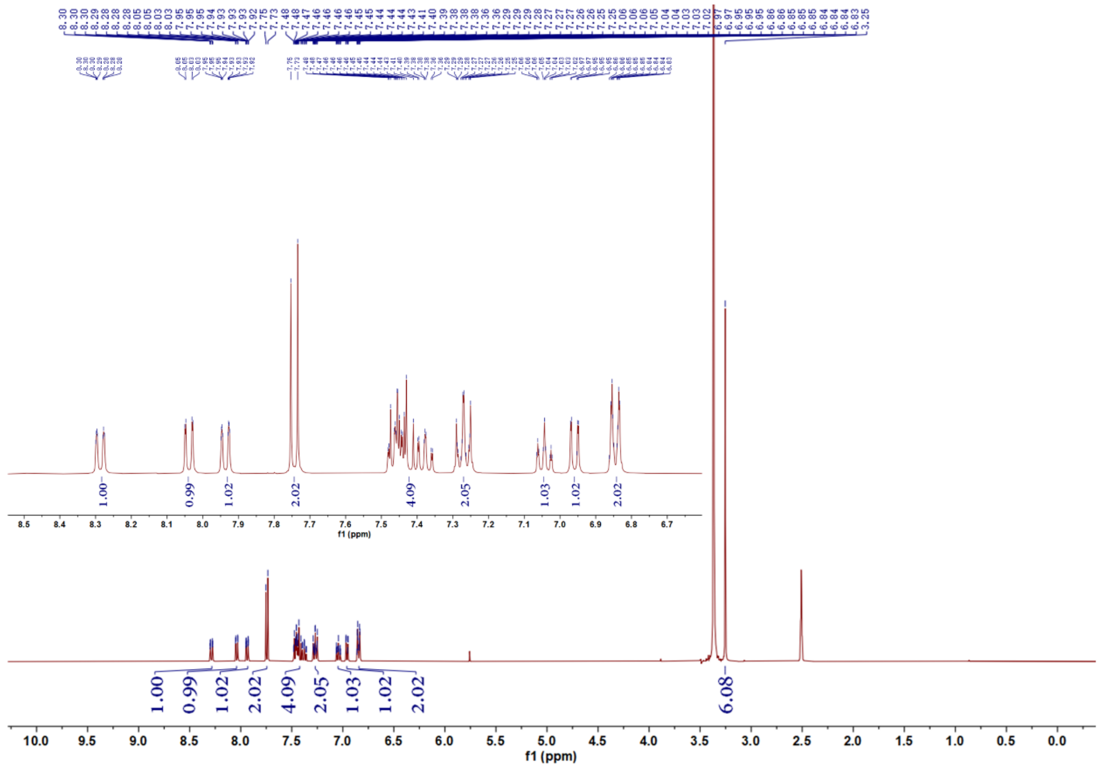

Figure **S4**. ^{1}H NMR spectrum of **2** in DMSO-d^{6}.

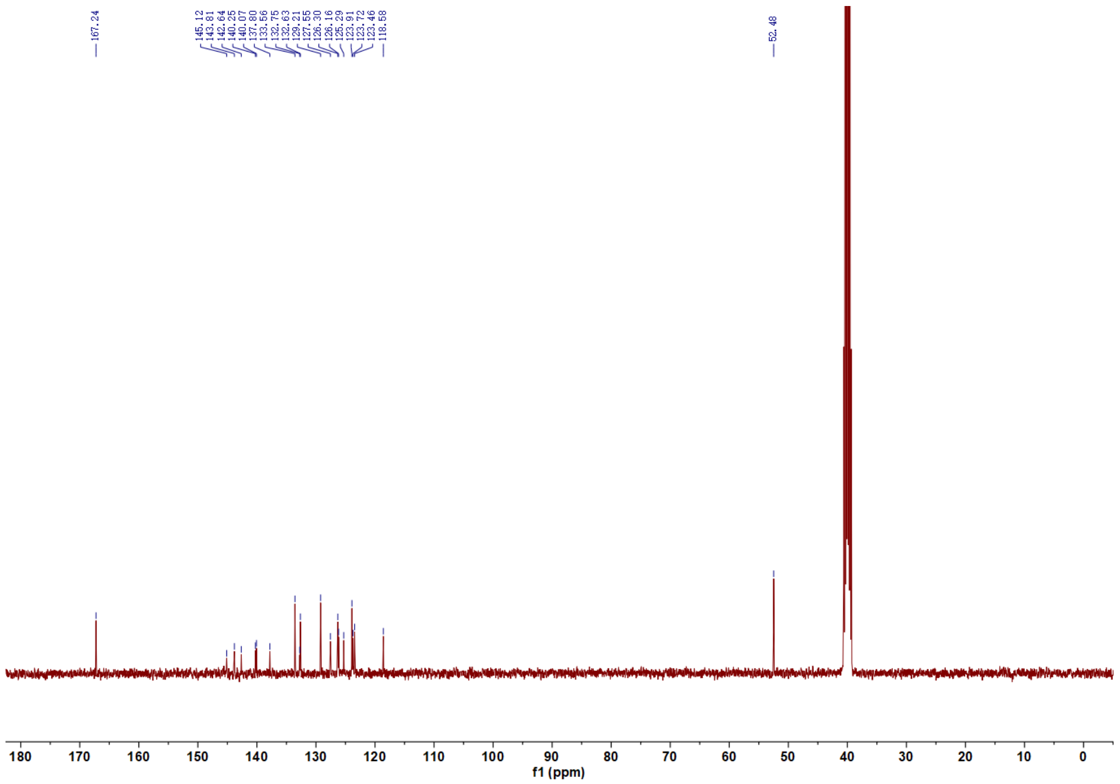

Figure **S5**. ^{13}C NMR spectrum of **2** in DMSO-d^{6}.

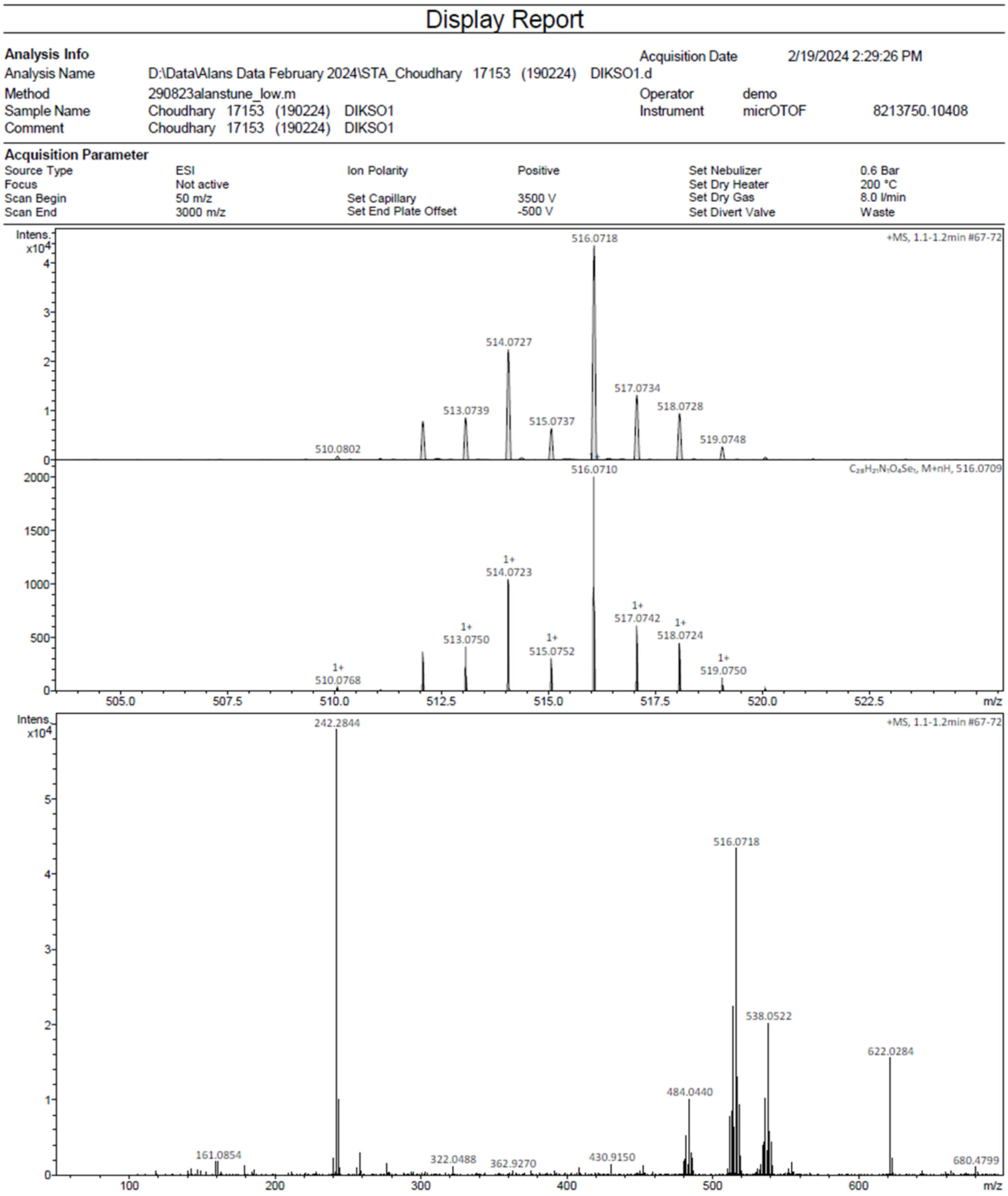

Figure **S6**. HRMS spectrum of **2**.

Benzo[4,5]selenopheno[3,2-*c*]quinolino[1,2,3-*fg*]acridine-4,17-dione (DiKTaSe)

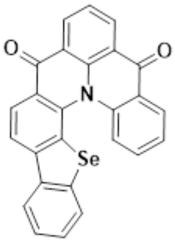

Compound **2** (300 mg, 0.583 mmol, 1.0 equiv.) was combined with sodium hydroxide (117 mg, 2.92 mmol, 5.0 equiv.) in 10 mL of an ethanol/water (1:1) mixture. The reaction mixture was heated to 100 °C for 18 h. After cooling to room temperature, the pH was adjusted to 2-3 by addition of dilute hydrochloric acid. The diacid was precipitated as a light yellow solid and was collected by vacuum filtration, washed thoroughly with water and dried under vacuum. Then the diacid (280 mg, 0.576 mmol, 1.0 equiv.) was dispersed in 10 mL dichloromethane under a nitrogen atmosphere. Thionyl chloride (105 µL, 1.44 mmol, 2.5 equiv.) and 2 drops of DMF were added to the reaction mixture sequentially. After 2 h under reflux, the reaction mixture was cooled to room temperature. 1M Tin(IV) chloride solution (2.88 mL, 2.88 mmol, 5.0 equiv.) was added slowly. After heating to 47 °C for 18 h, the reaction mixture was cooled to room temperature and quenched by dropwise addition of water. The resulting mixture was extracted with 3× 50 mL dichloromethane. The organic phase was then separated and concentrated under reduced pressure. The crude product was purified by column chromatography on silica gel (dichloromethane: hexane = 2: 1) to afford **DiKTaSe** as a yellow powder. **Yield** 66%, 172 mg. **Mp**: 343-344 °C. **R_f**: 0.3 (dichloromethane: hexane = 2: 1). **^{1}H NMR (500 MHz, CD_2Cl_2)** δ 8.64 (ddt, *J* = 7.5, 6.1, 1.3 Hz, 2H), 8.56 (dd, *J* = 8.3, 0.8 Hz, 1H), 8.45 – 8.41 (m, 1H), 8.34 – 8.28 (m, 2H), 7.84 – 7.80 (m, 1H), 7.68 – 7.61 (m, 2H), 7.57 – 7.52 (m, 3H), 7.48 (ddd, *J* = 8.1, 7.2, 1.2 Hz, 1H). **^{13}C NMR (126 MHz, CD_2Cl_2)** δ 179.15, 178.77, 144.00, 141.50, 140.88, 138.33, 136.87, 136.81, 132.10, 132.01, 131.95, 130.33, 128.63, 127.19, 126.78, 126.59, 125.46, 125.38, 125.13, 124.23, 124.11, 123.87, 123.80, 122.81, 120.18. **HRMS (ESI-MS)**: $[C_{26}H_{13}NO_2Se + H]^+$ **Calculated**: 452.0184; **Found:** 452.0202. **Anal. Calcd. For $C_{26}H_{13}NO_2Se$**: C 69.34%, H 2.91%, N 3.11% **Found**: C 69.33%, H 2.71%, N 2.86%. **HPLC** (75% Tetrahydrofuran and 25% Water): 99.02% pure, retention time 11.100 min.

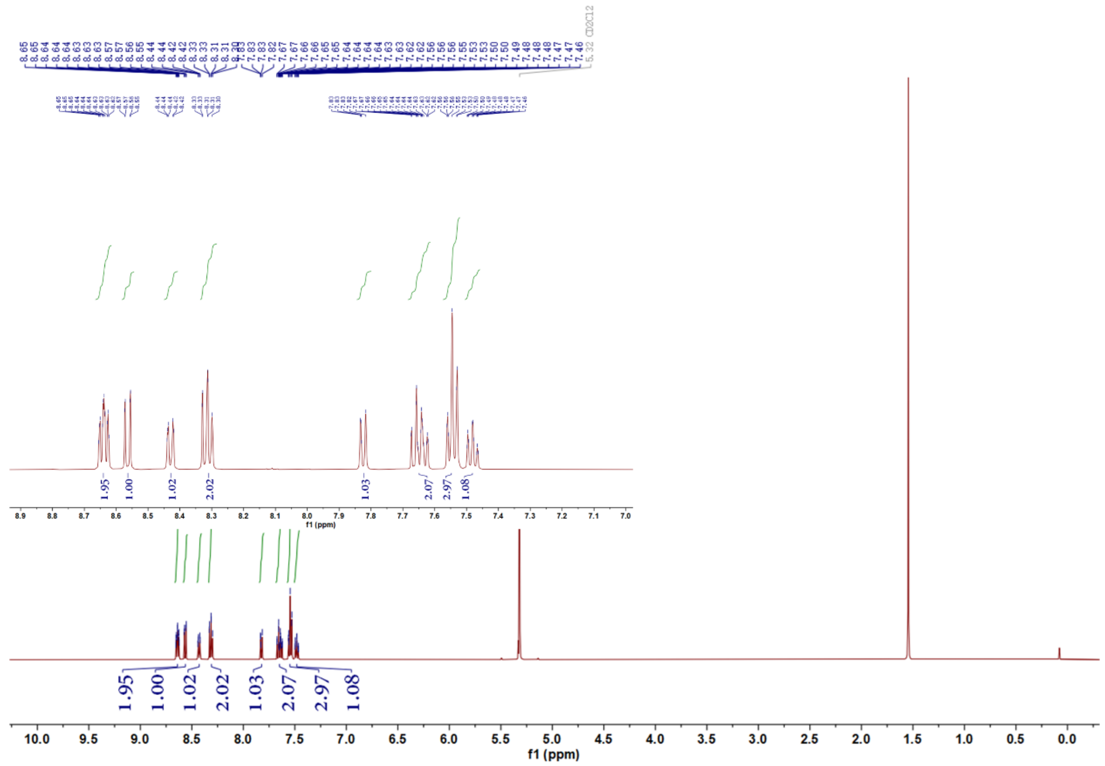

Figure **S7**. ^{1}H NMR spectrum of **DiKTaSe** in CD_2Cl_2.

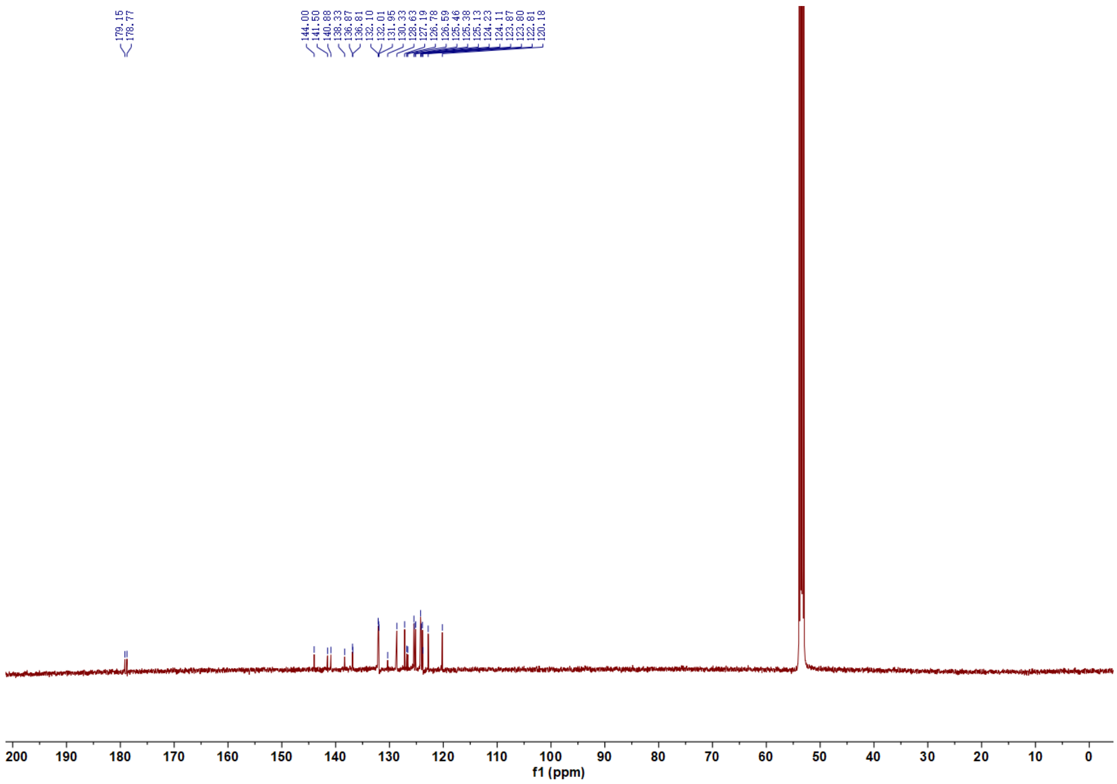

Figure **S8**. ^{13}C NMR spectrum of **DiKTaSe** in CD_2Cl_2.

Display Report

Analysis Info		Acquisition Date	2/19/2024 2:16:16 PM
Analysis Name	D:\Data\Alans Data February 2024\STA_Choudhary 17159 (190224) DIKTSE.d		
Method	290823alanstune_low.m	Operator	demo
Sample Name	Choudhary 17159 (190224) DIKTSE	Instrument	micrOTOF 8213750.10408
Comment	Choudhary 17159 (190224) DIKTSE		

Acquisition Parameter

Source Type	ESI	Ion Polarity	Positive	Set Nebulizer	0.6 Bar
Focus	Not active			Set Dry Heater	200 °C
Scan Begin	50 m/z	Set Capillary	3500 V	Set Dry Gas	8.0 l/min
Scan End	3000 m/z	Set End Plate Offset	-500 V	Set Divert Valve	Waste

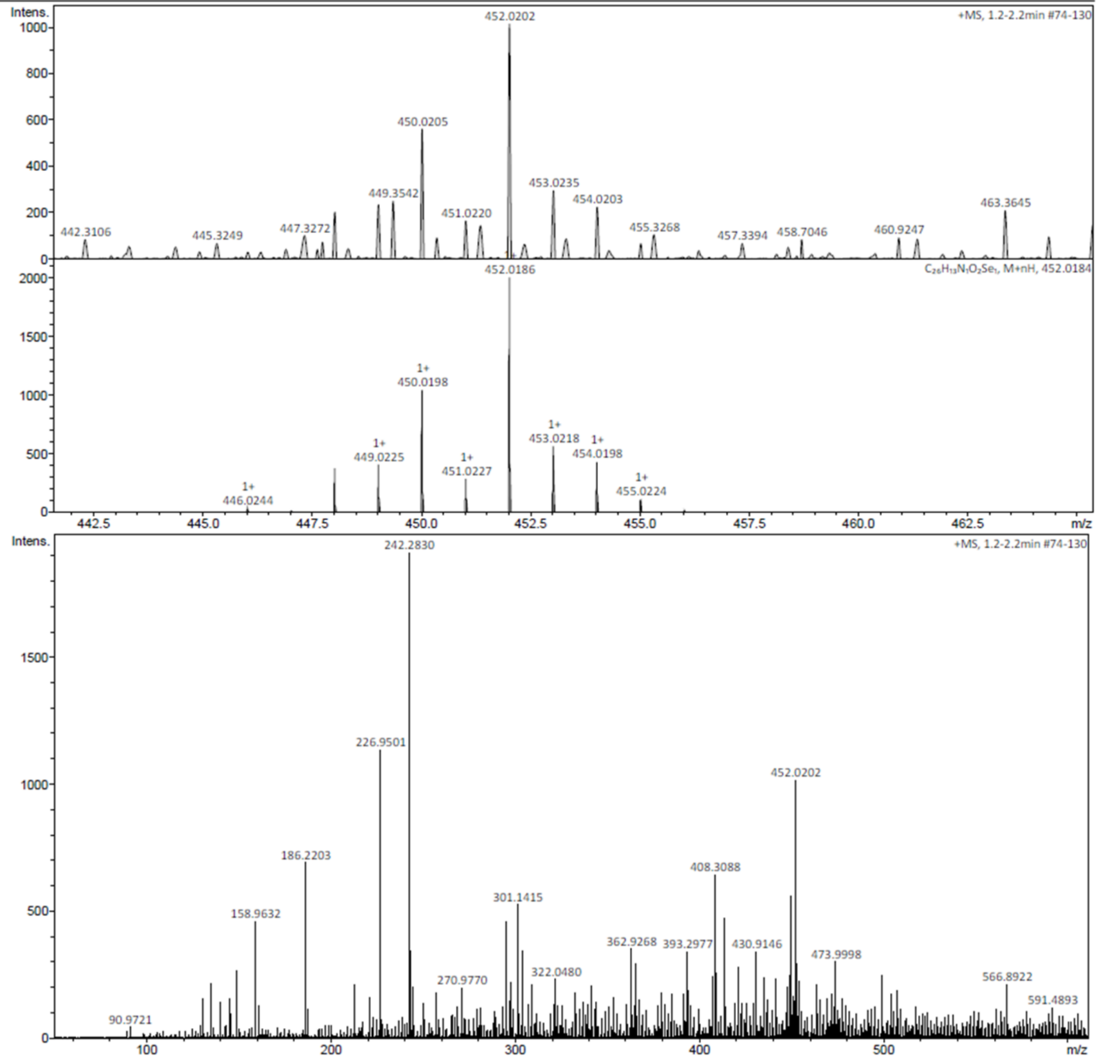

Figure **S9**. HRMS spectrum of **DiKTaSe**.

LONDON METROPOLITAN UNIVERSITY
HUMAN SCIENCES

Elemental Analysis Sample Results

**Name**	Jingxiang Wang
**Organisation Name**	University of St Andrews
**Purchase order number**	

**Standard – Acetanilide**		
**Element**	**Expected %**	**Found**
**Carbon**	71.10 (+/- 0.23)	71.02
**Hydrogen**	6.71 (+/- 0.07)	6.64
**Nitrogen**	10.34 (+/- 0.09)	10.29

**Analysis – DiKTaSe**			
**Element**	**Expected %**	**Found (1)**	**Found (2)**
**Carbon**	69.34	69.31	69.35
**Hydrogen**	2.91	2.66	2.75
**Nitrogen**	3.11	2.79	2.92

**Date completed**	16.04.2024
**Signature**	O. McCullough
**Comments**	

Figure **S10**. Elemental analysis data of **DiKTaSe**.

HPLC Trace Report24Aug2023

<Sample Information>

Sample Name : DiKTaSe
Sample ID :
Method Filename : 75% THF 25% water 0.6 mlmin 20 mins.lcm
Batch Filename : TRZ_TCZ_Ph_C12.lcb
Vial # : 2-29 Sample Type : Unknown
Injection Volume : 10 uL
Date Acquired : 22/08/2023 12:54:41 Acquired by : System Administrator
Date Processed : 22/08/2023 13:14:44 Processed by : System Administrator

<Chromatogram>

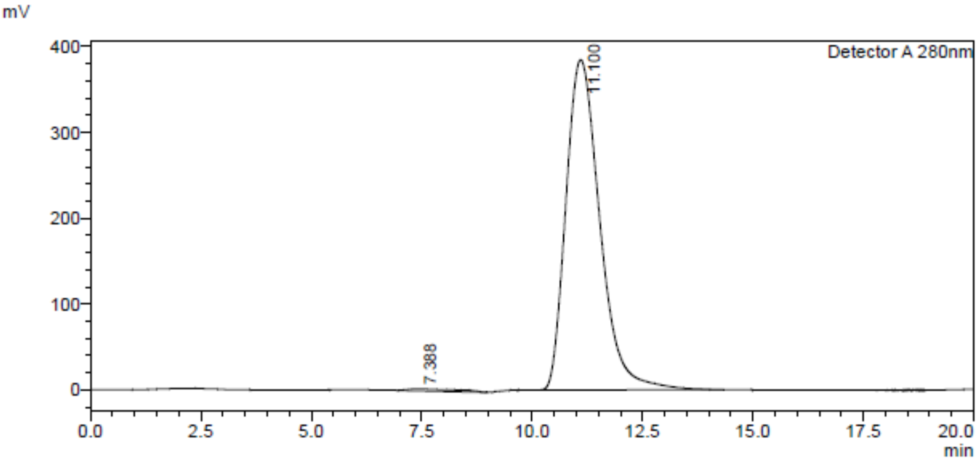

<Peak Table>

Detector A 280nm

Peak#	Ret. Time	Area	Height	Area%	Area/Height	Width at 5% Height
1	7.388	202469	2086	0.977	97.050	1.917
2	11.100	20524186	384664	99.023	53.356	1.788
Total		20726655	386750	100.000		

Figure **S11**. HPLC spectrum of **DiKTaSe**.

***N*-(2'-(3,6-di-*tert*-butyl-9*H*-carbazol-9-yl)-[1,1'-biphenyl]-4-yl)dibenzo[*b*,*d*]selenophen-4-amine (3)**

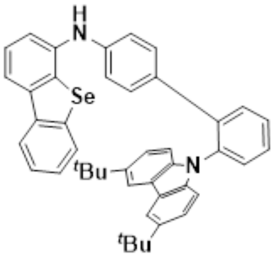

4-bromodibenzo[*b*,*d*]selenophene (450 mg, 1.45 mmol, 1.0 equiv.), 2'-(3,6-di-*tert*-butyl-9*H*-carbazol-9-yl)-[1,1'-biphenyl]-4-amine (681 mg, 1.52 mmol, 1.05 equiv.), $Pd_2(dba)_3$ (39.9 mg, 0.044 mmol, 0.03 equiv.), tri-tert-butylphosphonium tetrafluoroborate (33.7 mg, 0.116 mmol, 0.08 equiv.), NaO*t*-Bu (418 mg, 4.35 mmol, 3 equiv.) and toluene (20 mL) were added to a 2-neck Schlenk tube. The mixture was stirred at 105 °C for 48 h under a nitrogen atmosphere. After cooling to room temperature, the reaction was quenched by adding water and extracted with 3× 50 mL dichloromethane. The organic phase was then separated and concentrated under reduced pressure. The crude product was purified by column chromatography on silica gel (ethyl acetate: hexane = 1: 8) to afford compound **1** as a brown solid. **Yield** 52%, 510 mg. **Mp**: 213-214 °C. **R*f***: 0.5 (ethyl acetate: hexane = 1: 10). **^{1}H NMR (500 MHz, DMSO-*d*^{6})** δ 8.23 (d, *J* = 13.8 Hz, 3H), 8.02 (d, *J* = 9.3 Hz, 2H), 7.90 (d, *J* = 7.7 Hz, 1H), 7.65 (dd, *J* = 27.3, 7.6 Hz, 2H), 7.56 – 7.29 (m, 7H), 6.96 (dd, *J* = 34.9, 8.1 Hz, 5H), 6.62 (d, *J* = 8.2 Hz, 2H), 1.38 (s, 18H). **^{13}C NMR (126 MHz, DMSO-*d*^{6})** δ 143.07, 142.21, 140.64, 140.51, 139.82, 139.67, 139.01, 138.82, 134.79, 132.39, 131.56, 130.27, 129.85, 129.32, 128.83, 128.69, 127.56, 126.61, 126.54, 125.46, 123.86, 123.78, 123.06, 117.83, 117.53, 117.10, 116.93, 109.62, 34.90, 32.32. **HRMS (ESI-MS)**: $[C_{44}H_{40}N_2Se + H]^+$ **Calculated**: 677.2429; **Found:** 677.2409.

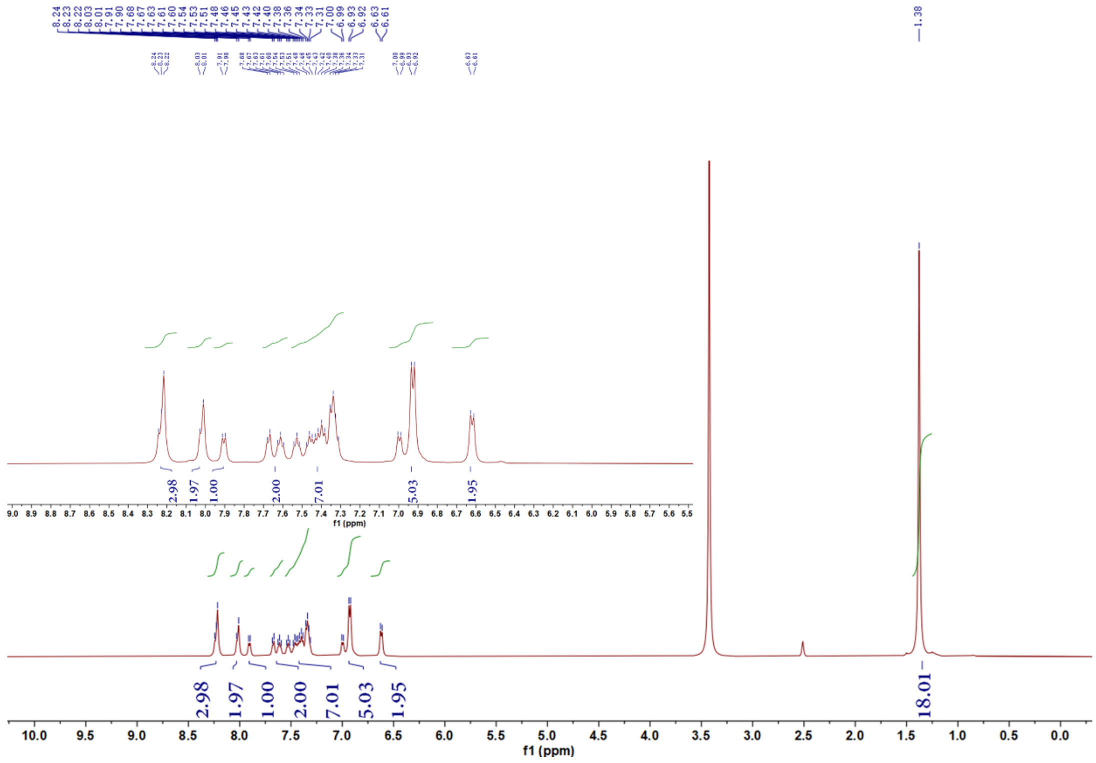

Figure **S12**. ^{1}H NMR spectrum of **3** in DMSO-d^6.

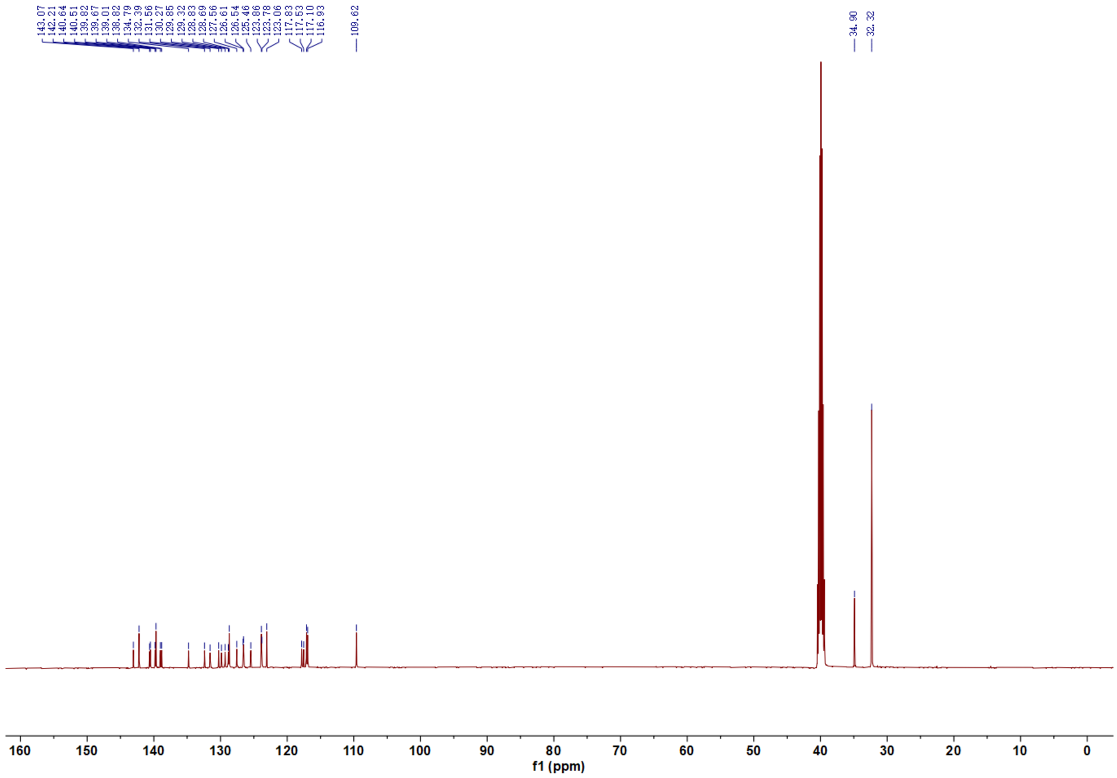

Figure **S13**. ^{13}C NMR spectrum of **3** in DMSO-d^6.

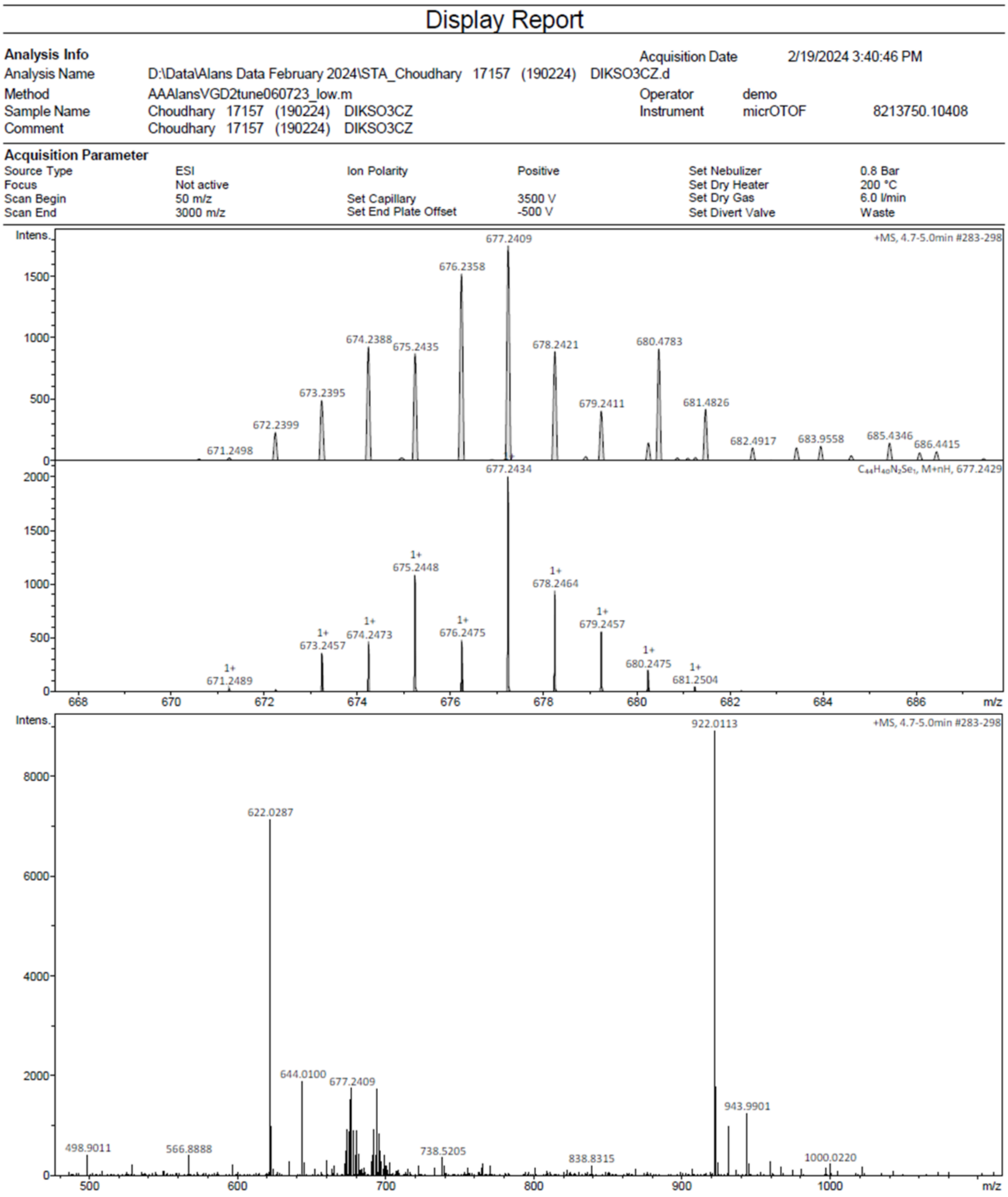

Figure **S14**. HRMS spectrum of **3**.

Dimethyl 2-((2'-(3,6-di-*tert*-butyl-9*H*-carbazol-9-yl)-[1,1'-biphenyl]-4-yl)(dibenzo[*b*,*d*] selenophen-4-yl)amino)isophthalate (4)

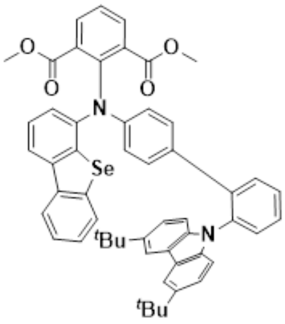

Compound **3** (450 mg, 0.666 mmol, 1.0 equiv.), dimethyl 2-bromoisophthalate (327 mg, 1.20 mmol, 1.8 equiv.), potassium carbonate (276 mg, 2.00 mmol, 3.0 equiv.), copper(I) iodide (25.4 mg, 0.133 mmol, 0.2 equiv.), 2,2,6,6-tetramethyl-3,5-heptanedione (27.7 µL, 0.133 mmol, 0.2 equiv.), copper tin alloy (24.3 mg, 0.133 mmol, 0.2 equiv.) and anhydrous di-*n*-butyl ether (15 mL) were added to a 2-neck Schlenk tube. The resulting reaction mixture was heated to 150 °C under nitrogen and stirred for 3 days. After cooling to room temperature, the reaction was quenched by adding water and extracted with 3× 50 mL dichloromethane. The organic phase was then separated and concentrated under reduced pressure. The crude product was purified by column chromatography on silica gel (ethyl acetate: hexane= 1: 15) to afford compound **4** as a pale-yellow powder. **Yield** 62%, 357 mg. **Mp**: 264-265 °C. **R*f***: 0.3 (ethyl acetate: hexane= 1: 4). **^{1}H NMR (500 MHz, DMSO-*d*^{6})** δ 8.26 – 8.19 (m, 3H), 7.97 (dd, *J* = 8.0, 1.1 Hz, 1H), 7.92 – 7.88 (m, 1H), 7.73 – 7.63 (m, 4H), 7.56 (td, *J* = 7.5, 1.6 Hz, 1H), 7.47 – 7.34 (m, 6H), 7.30 (t, *J* = 7.8 Hz, 1H), 7.08 – 7.00 (m, 2H), 6.94 (d, *J* = 8.6 Hz, 2H), 6.72 (dd, *J* = 7.9, 1.0 Hz, 1H), 6.52 – 6.46 (m, 2H), 2.91 (s, 6H), 1.39 (s, 18H). **^{13}C NMR (126 MHz, DMSO-*d*^{6})** δ 166.89, 144.22, 143.34, 142.34, 142.02, 140.29, 140.17, 139.94, 139.88, 137.72, 134.95, 133.71, 133.02, 132.77, 132.40, 131.83, 130.04, 129.45, 129.37, 128.45, 127.56, 126.50, 126.04, 125.94, 125.25, 123.99, 123.70, 123.62, 123.20, 122.54, 118.74, 116.93, 109.59, 52.16, 34.91, 32.31. **HRMS (ESI-MS)**: [$C_{54}H_{48}N_2O_4Se$ +H]$^+$ **Calculated**: 869.2857; **Found:** 869.2852.

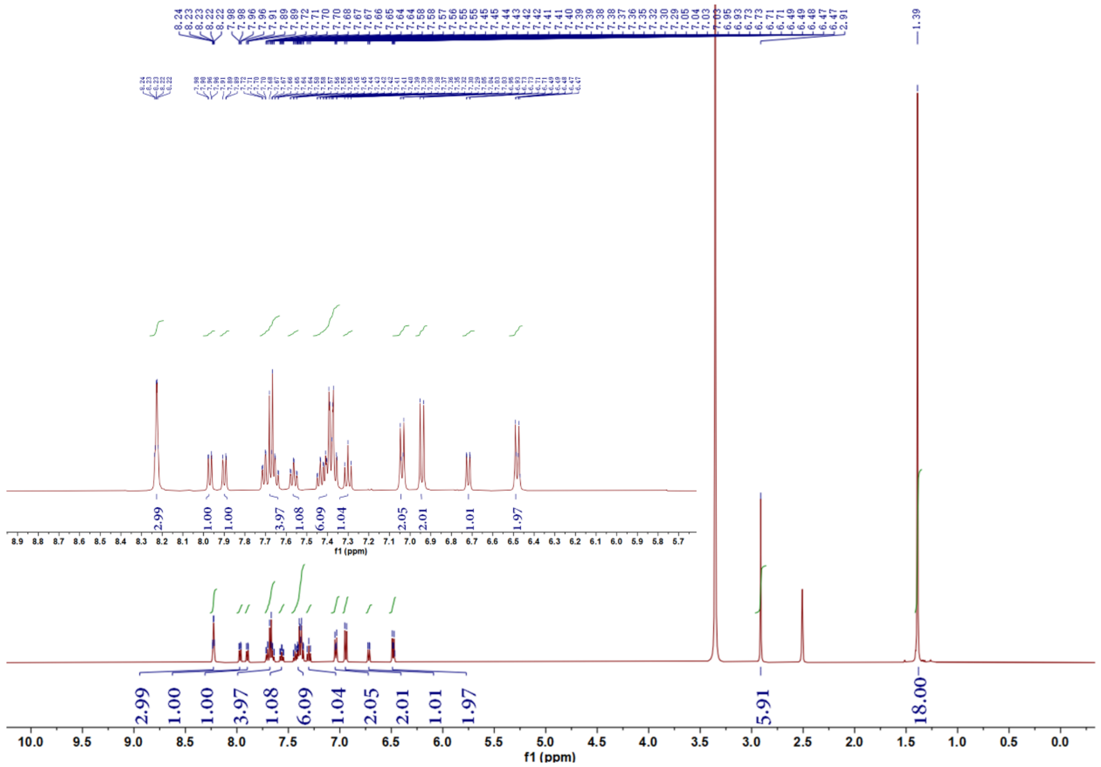

Figure **S15**. ^{1}H NMR spectrum of **4** in DMSO-d^6.

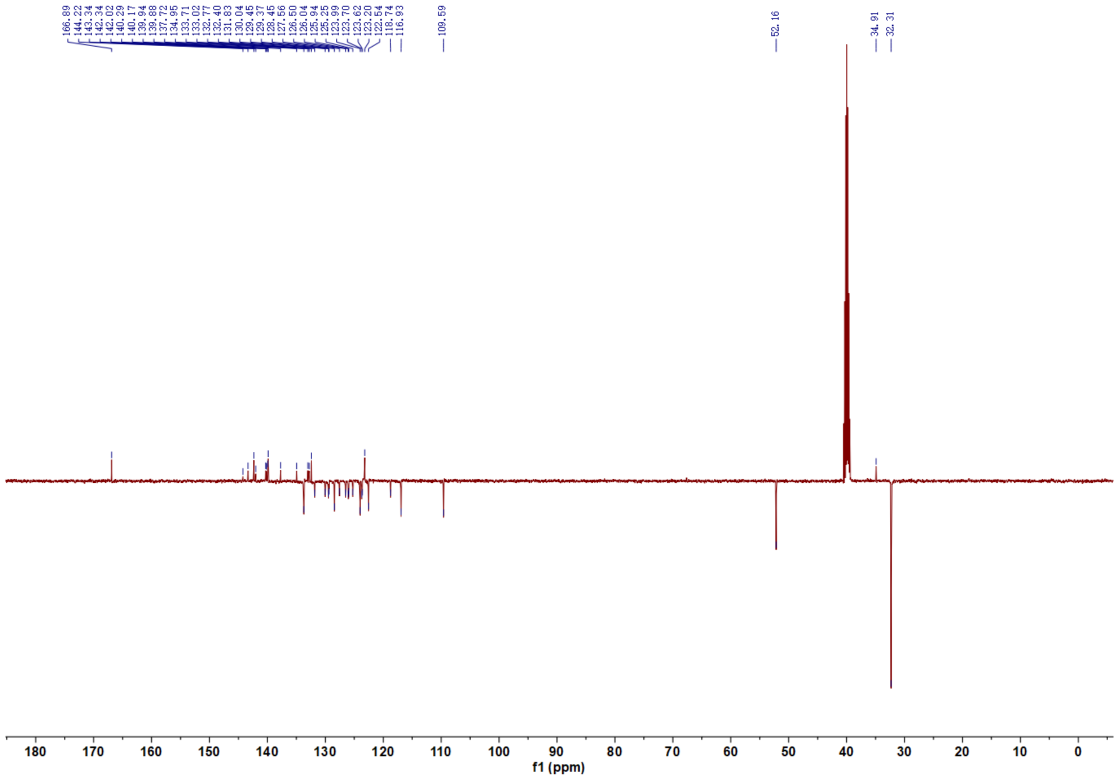

Figure **S16**. DEPTQ ^{13}C NMR spectrum of **4** in DMSO-d^6.

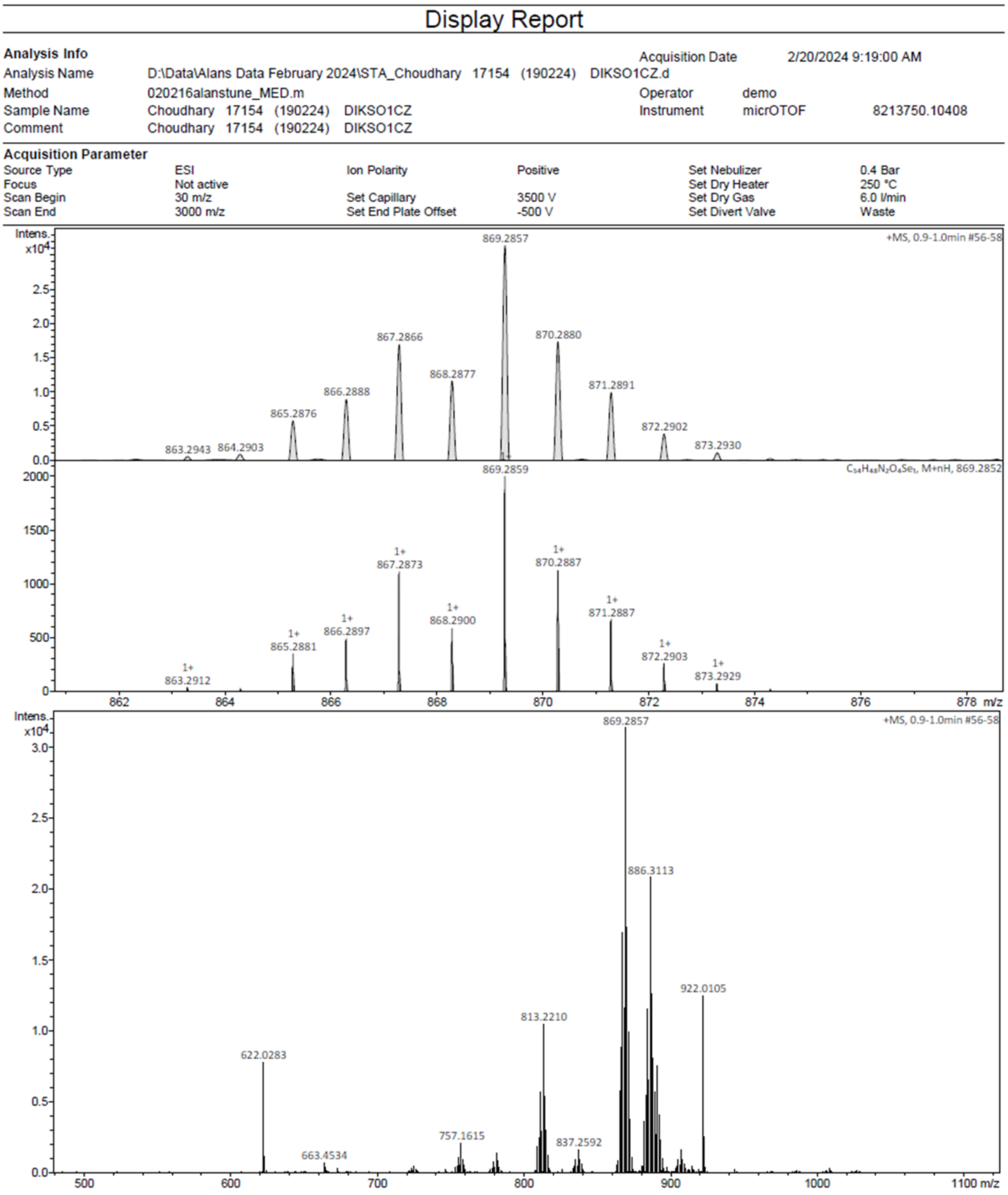

Figure **S17**. HRMS spectrum of **4**.

15-(2-(3,6-di-*tert*-butyl-9*H*-carbazol-9-yl)phenyl)benzo[4,5]selenopheno[3,2-*c*]quinolino[1,2,3-*fg*]acridine-4,17-dione (tBuCz-DiKTaSe)

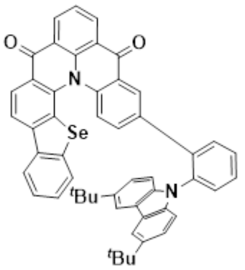

Compound **4** (320 mg, 0.369 mmol, 1.0 equiv.) was combined with sodium hydroxide (148 mg, 3.69 mmol, 10 equiv.) in 15 mL of an ethanol/water/1,4-dioxane (1:1:1) mixture. The reaction mixture was heated to 100 °C for 18 h. After cooling to room temperature, the pH was adjusted to 2-3 by addition of dilute hydrochloric acid. The diacid was precipitated as a light yellow solid and was collected by vacuum filtration, washed thoroughly with water and dried under vacuum. Then the diacid (300 mg, 0.357 mmol, 1.0 equiv.) was dispersed in 10 mL dichloromethane under a nitrogen atmosphere. Thionyl chloride (65 μL, 0.893 mmol, 2.5 equiv.) and 2 drops of DMF were added to the reaction mixture sequentially. After 2 h under reflux, the reaction mixture was cooled to room temperature. 1M Tin(IV) chloride solution (1.43 mL, 1.43 mmol, 4.0 equiv.) was added slowly. After heating to 47 °C for 18 h, the reaction mixture was cooled to room temperature and quenched by dropwise addition of water. The resulting mixture was extracted with 3× 50 mL dichloromethane. The organic phase was then separated and concentrated under reduced pressure. The crude product was purified by column chromatography on silica gel (dichloromethane: hexane = 3: 2) and washed with methanol to afford **tBuCz-DiKTaSe** as a yellow powder. **Yield** 38%, 109 mg. **Mp**: 284-285 °C. **R_f**: 0.15 (dichloromethane: hexane = 3: 1). **^{1}H NMR (500 MHz, CD_2Cl_2)** δ 8.70 (dd, *J* = 7.7, 1.7 Hz, 1H), 8.63 (dd, *J* = 7.5, 1.8 Hz, 2H), 8.50 (d, *J* = 8.3 Hz, 1H), 8.30 (d, *J* = 1.8 Hz, 1H), 8.23 (dd, *J* = 8.3, 3.0 Hz, 2H), 8.17 (d, *J* = 1.9 Hz, 1H), 8.08 (d, *J* = 7.9 Hz, 1H), 7.88 (dd, *J* = 7.6, 1.7 Hz, 1H), 7.72 – 7.61 (m, 4H), 7.58 – 7.52 (m, 2H), 7.42 (ddd, *J* = 12.8, 8.6, 1.9 Hz, 2H), 7.27 (d, *J* = 8.5 Hz, 1H), 7.14 – 7.08 (m, 2H), 7.01 (d, *J* = 8.6 Hz, 1H), 1.38 (s, 18H). **^{13}C NMR (126 MHz, CD_2Cl_2)** δ 178.64, 178.51, 143.91, 142.96, 142.63, 141.60, 140.61, 139.86, 139.10,

137.99, 136.73, 136.55, 135.64, 135.42, 132.15, 131.99, 131.54, 131.30, 130.55, 129.55, 129.26, 128.63, 128.45, 126.66, 126.53, 126.36, 125.28, 124.69, 124.10, 124.00, 123.87, 123.83, 123.68, 123.41, 123.16, 122.60, 119.96, 116.20, 115.55, 109.96, 109.27, 34.59, 34.52, 31.67, 31.64. **HRMS (ESI-MS)**: $[C_{52}H_{40}N_2O_2Se + H]^+$ **Calculated**: 805.2328; **Found:** 805.2322. **Anal. Calcd. For $C_{52}H_{40}N_2O_2Se$**: C 77.70%, H 5.02%, N 3.48% **Found**: C 77.38%, H 5.02%, N 3.17%. **HPLC** (70% Tetrahydrofuran and 30% Water): 98.636% pure, retention time 6.268 min.

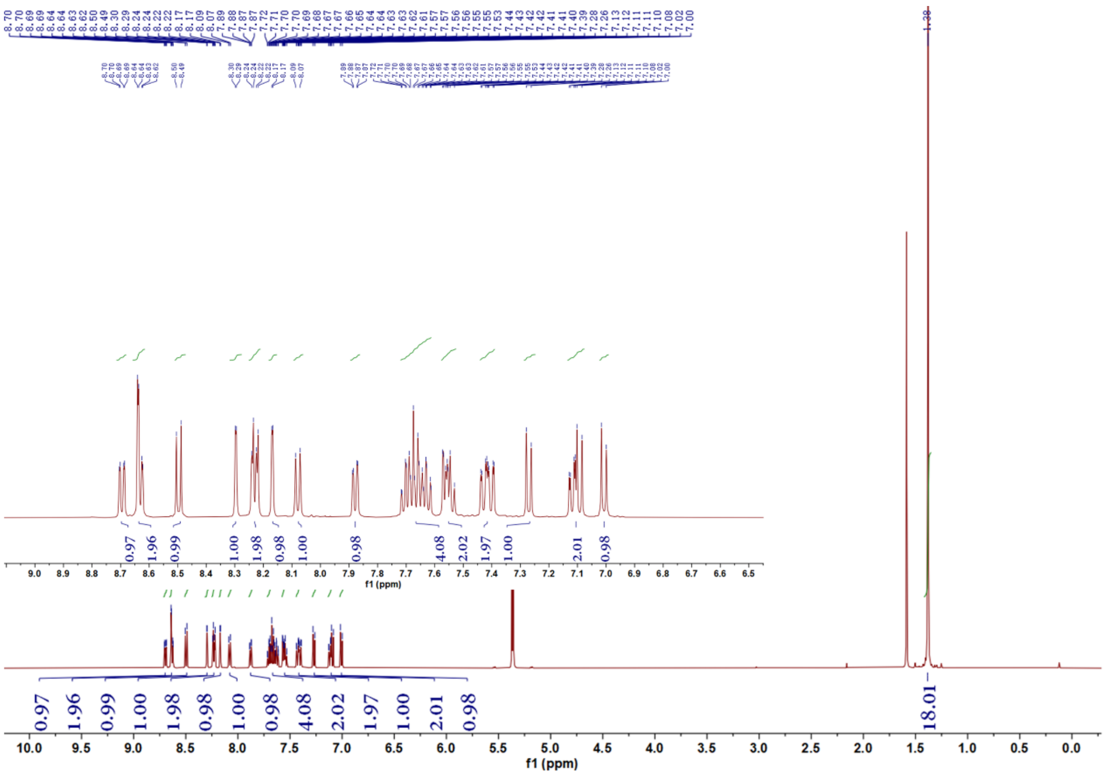

Figure **S18**. ^{1}H NMR spectrum of **tBuCz-DiKTaSe** in CD_2Cl_2.

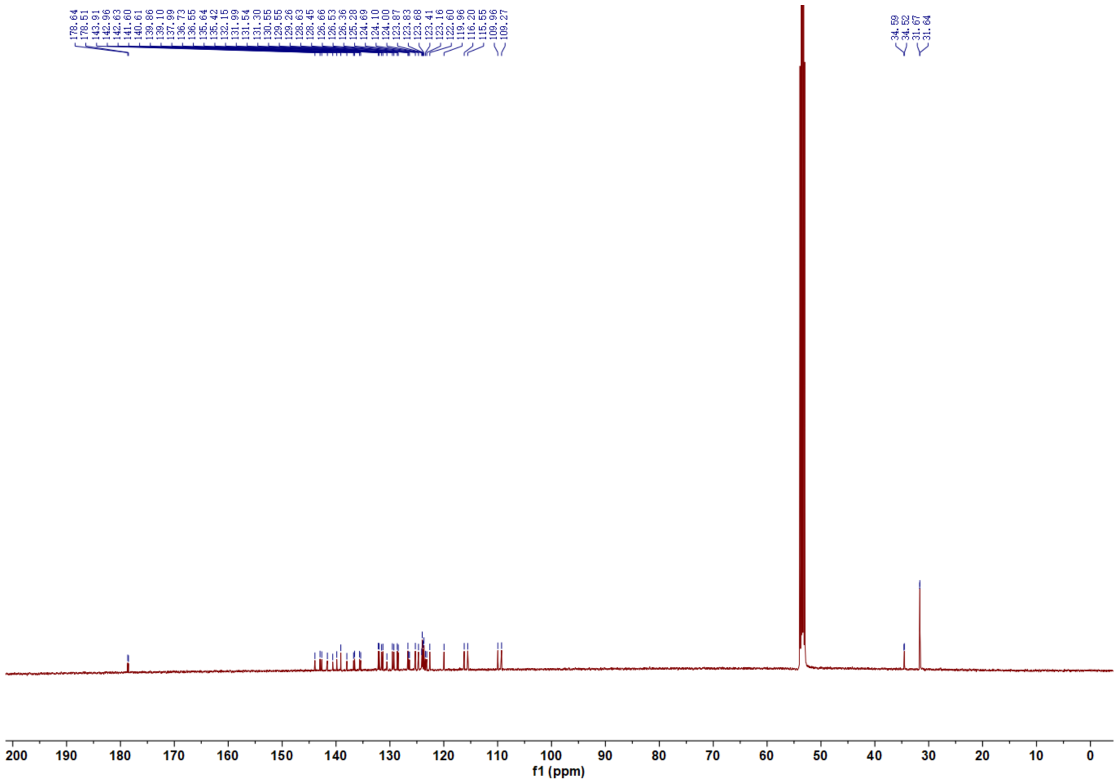

Figure **S19**. ^{13}C NMR spectrum of **tBuCz-DiKTaSe** in CD_2Cl_2.

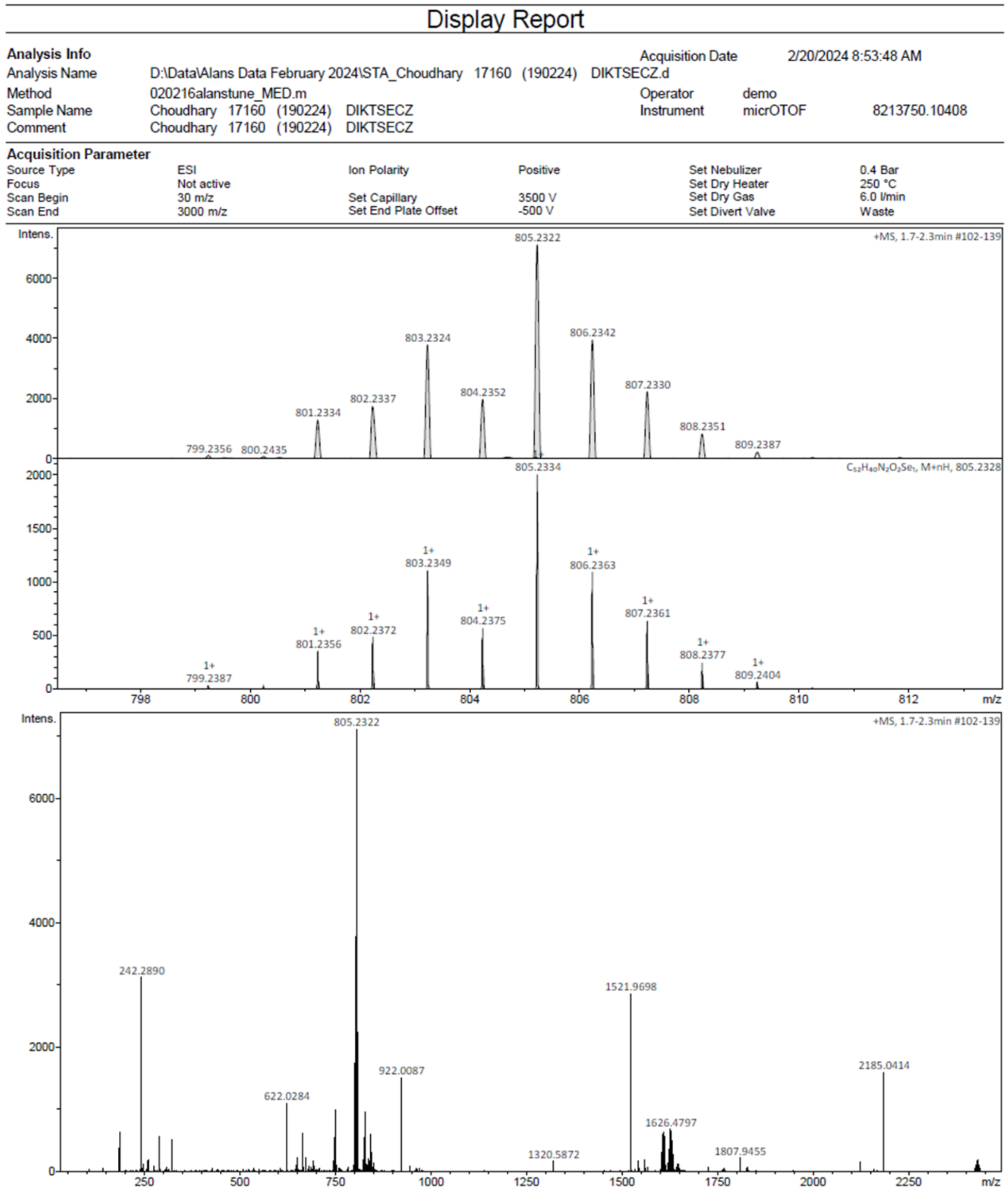

Figure **S20**. HRMS spectrum of **tBuCz-DiKTaSe**.

LONDON METROPOLITAN UNIVERSITY
HUMAN SCIENCES

Elemental Analysis Sample Results

**Name**	Jingxiang Wang
**Organisation Name**	University of St Andrews
**Purchase order number**	

**Standard – Acetanilide**		
**Element**	**Expected %**	**Found**
**Carbon**	71.10 (+/- 0.23)	71.02
**Hydrogen**	6.71 (+/- 0.07)	6.64
**Nitrogen**	10.34 (+/- 0.09)	10.29

**Analysis – tBuCz-DiKTaSe**			
**Element**	**Expected %**	**Found (1)**	**Found (2)**
**Carbon**	77.70	77.27	77.48
**Hydrogen**	5.02	5.08	4.96
**Nitrogen**	3.48	3.15	3.18

**Date completed**	16.04.2024
**Signature**	O. McCullough
**Comments**	

Figure **S21**. Elemental analysis data of **tBuCz-DiKTaSe**.

HPLC Trace Report13Jan2024

<Sample Information>

Sample Name : tbuczdiktase
Sample ID :
Method Filename : 70% THF 30% water 0.6 mlmin 20 mins.lcm
Batch Filename : JMDS_208.lcb
Vial # : 1-18
Sample Type : Unknown
Injection Volume : 5 uL
Date Acquired : 12/01/2024 18:26:57
Acquired by : System Administrator
Date Processed : 12/01/2024 18:46:59
Processed by : System Administrator

<Chromatogram>

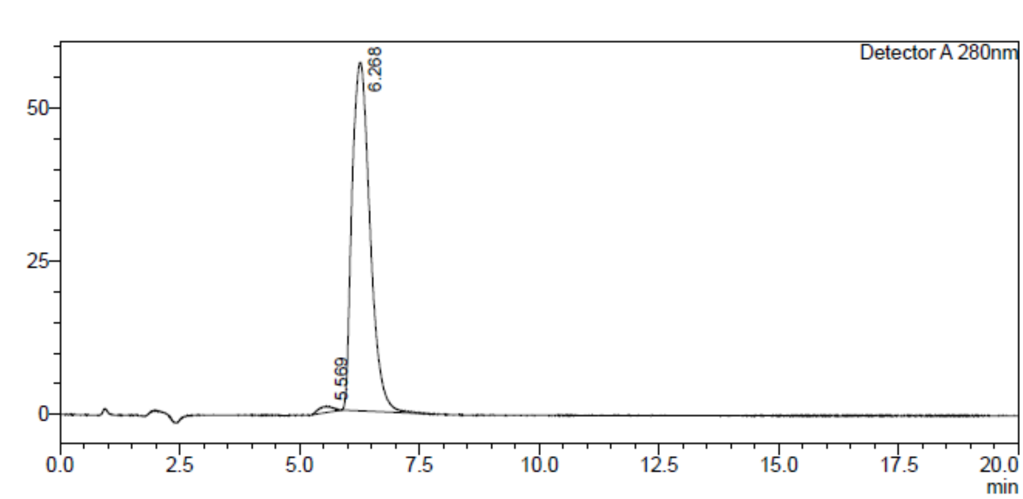

<Peak Table>

Detector A 280nm

Peak#	Ret. Time	Area	Height	Area%	Area/Height	Width at 5% Height
1	5.569	20494	966	1.364	21.224	0.554
2	6.268	1481800	56883	98.636	26.050	0.830
Total		1502294	57848	100.000		

Figure **S22**. HPLC spectrum of **tBuCz-DiKTaSe**.

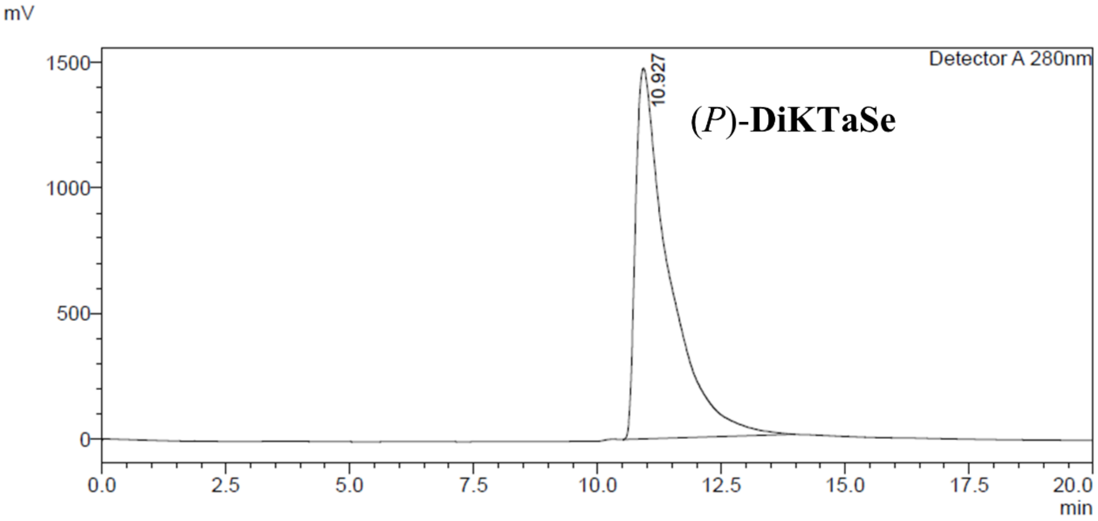

<Peak Table>

Detector A 280nm

Peak#	Ret. Time	Area	Height	Area%	Area/Height	Width at 5% Height
1	10.927	69377068	1476556	100.000	46.986	1.955
Total		69377068	1476556	100.000		

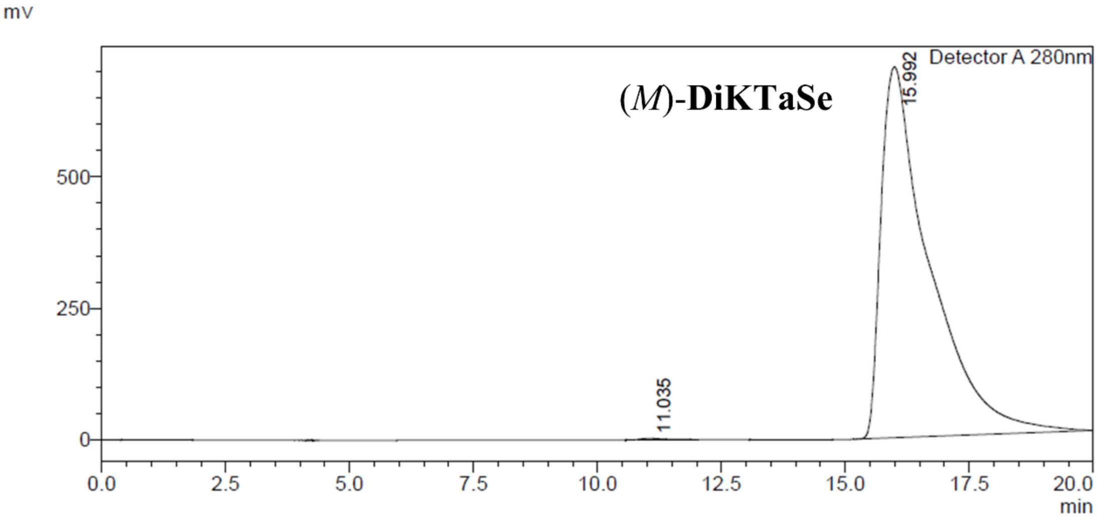

<Peak Table>

Detector A 280nm

Peak#	Ret. Time	Area	Height	Area%	Area/Height	Width at 5% Height
1	11.035	54759	1814	0.114	30.180	1.091
2	15.992	48174032	705102	99.886	68.322	2.682
Total		48228791	706916	100.000		

Figure **S23**. Chiral HPLC spectra of (*P*)-**DiKTaSe** and (*M*)-**DiKTaSe** (Column: CHIRALPAK IF; Solvent system: Hexane:DCM 5:95; Detector: 280nm).

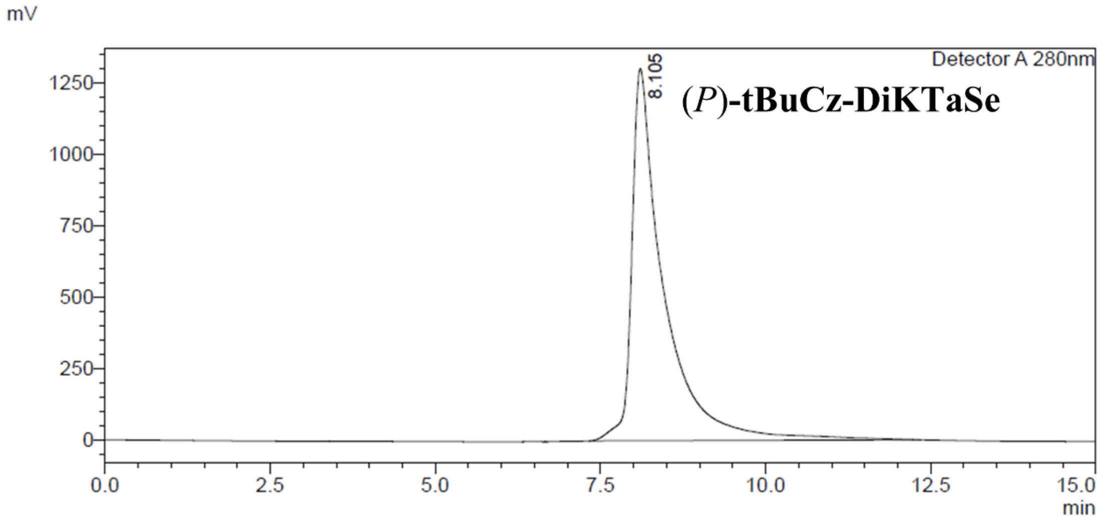

<Peak Table>

Detector A 280nm

Peak#	Ret. Time	Area	Height	Area%	Area/Height	Width at 5% Height
1	8.105	44074803	1302118	100.000	33.849	1.504
Total		44074803	1302118	100.000		

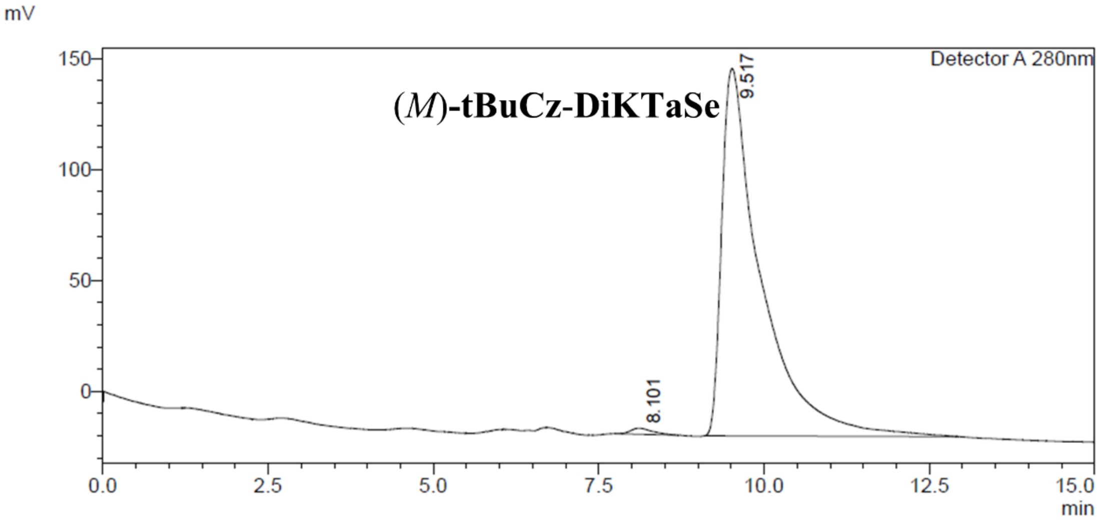

<Peak Table>

Detector A 280nm

Peak#	Ret. Time	Area	Height	Area%	Area/Height	Width at 5% Height
1	8.101	62054	2621	0.896	23.677	0.795
2	9.517	6861436	165483	99.104	41.463	1.794
Total		6923491	168104	100.000		

Figure **S24**. Chiral HPLC spectra of (*P*)-**tBuCz-DiKTaSe** and (*M*)-**tBuCz-DiKTaSe** (Column: CHIRALPAK IF; Solvent system: DCM 100%; Detector: 280nm).

X-ray Crystallography

X-ray diffraction data for **DiKTaSe** and **tBuCz-DiKTaSe** were collected at 173 K using a Rigaku FR-X Ultrahigh Brilliance Microfocus RA generator/confocal optics with XtaLAB P200 diffractometer [Mo Kα radiation (λ = 0.71073 Å)]. Data for all compounds analysed were collected (using a calculated strategy) and processed (including correction for Lorentz, polarization and absorption) using CrysAlisPro.[21] Structures were solved by dual-space methods (SHELXT[22]) and refined by full-matrix least-squares against F^2 (SHELXL-2019/3[23]). Non-hydrogen atoms were refined anisotropically. Crystals of **tBuCz-DiKTaSe** were affected by non-merohedral twinning, the twin fraction refined to 0.326(3). The HKL for refinement was calculated using twinrotmat running in PLATON.[24] All other calculations were performed using the Olex2[25] interface. Selected crystallographic data are presented in Table **S1**. CCDC 2433095-2433096 contains the supplementary crystallographic data for this paper. These data can be obtained free of charge from The Cambridge Crystallographic Data Centre via www.ccdc.cam.ac.uk/structures.

Table **S1**. Selected crystallographic data.

	**DiKTaSe**	**tBuCz-DiKTaSe**
formula	$C_{26}H_{13}NO_2Se$	$C_{54}H_{46}Cl_2N_2O_3Se$
fw	450.33	920.79
temperature [K]	173	173
crystal description	Yellow prism	Yellow plate
crystal size [mm^3]	0.12 × 0.11 × 0.09	0.08 × 0.05 × 0.007
space group	$P2_1/c$	$P\bar{1}$
a [Å]	8.44323(15)	11.1415(3)
b [Å]	12.38411(18)	11.5572(3)
c [Å]	17.3647(3)	17.8628(8)
α [°]		81.018(3)
β [°]	96.7347(16)	83.942(3)
γ [°]		75.253(2)
vol [Å]^3	1803.16(5)	2191.84(13)
Z	4	2
ρ (calc) [g/cm^3]	1.659	1.395
μ [mm^{-1}]	2.108	1.027
F(000)	904	952
reflections collected	38676	39758
independent reflections (R_{int})	4446 (0.0276)	10309 (0.0278)
parameters, restraints	271, 0	615, 95

GoF on F^2	1.063	1.178
R_1 [$I > 2\sigma(I)$]s	0.0294	0.0796
wR_2 (all data)	0.0676	0.2157
largest diff. peak/hole [e/Å^3]	0.41/-0.28	1.55/-0.92

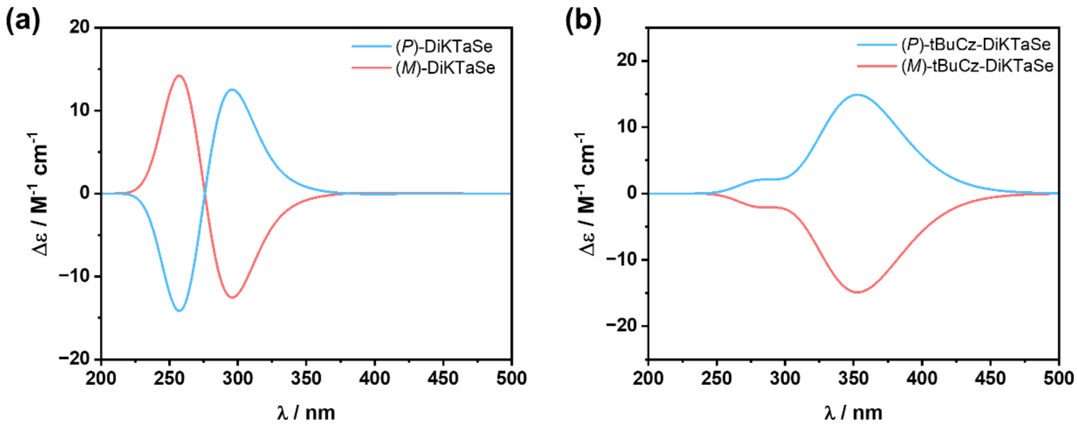

Figure **S25**. Theoretically simulated CD spectra of (*P*)/(*M*)-**DiKTaSe** and (*P*)/(*M*)-**tBuCz-DiKTaSe** calculated at the TDA-DFT/M06-2X/def2-TZVP in toluene.

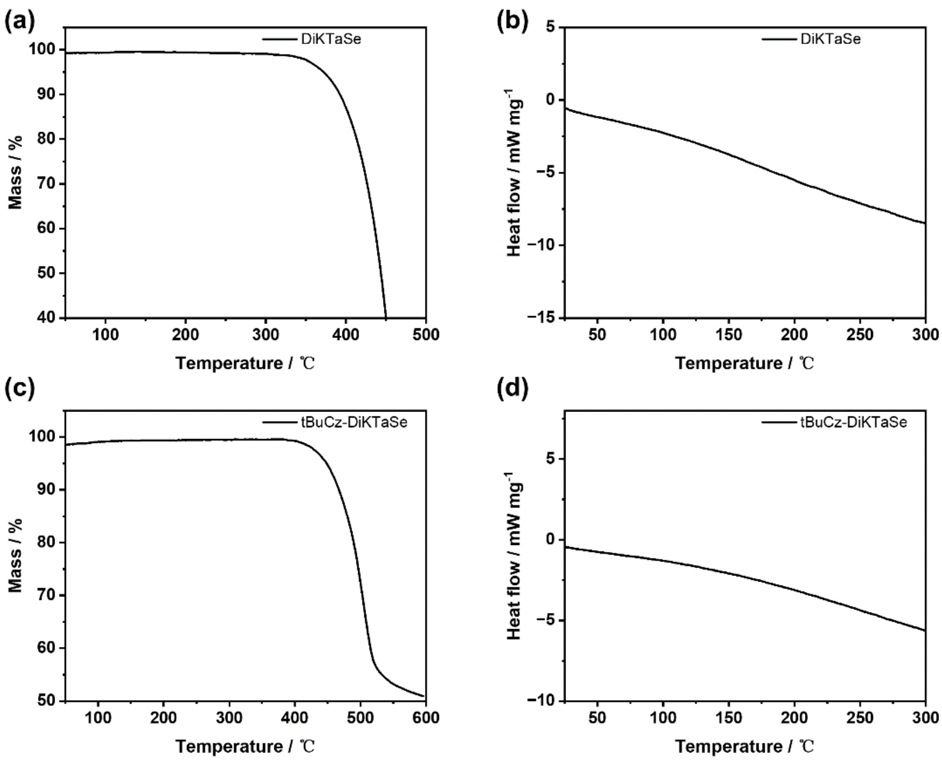

Figure **S26**. (a) TGA and (b) DSC analyses of **DiKTaSe** and (c) TGA and (d) DSC analyses of **tBuCz-DiKTaSe**.

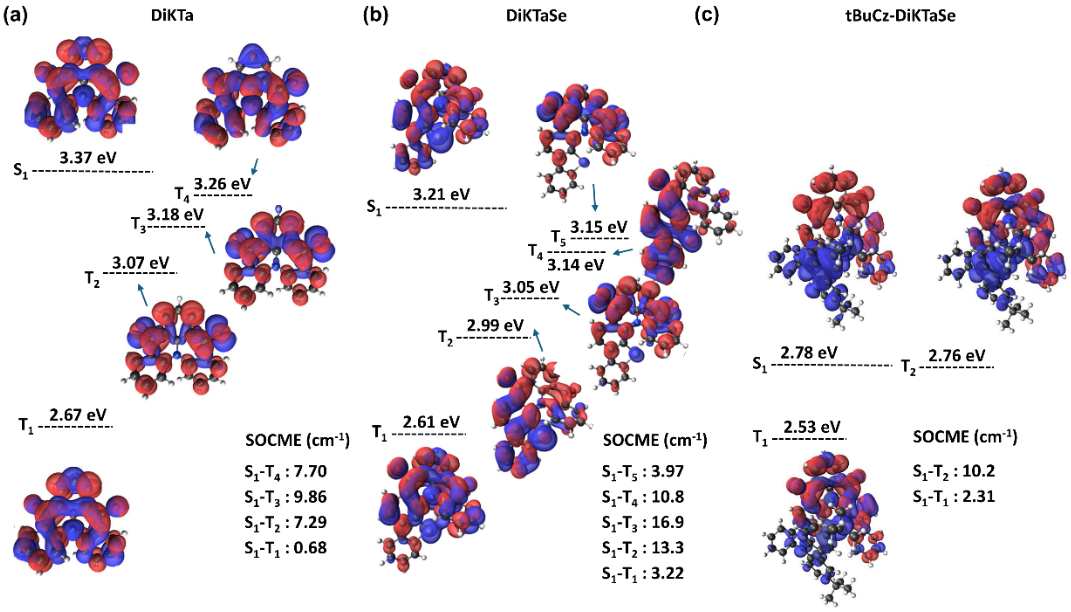

Figure **S27**. NTOs (particle and hole are represented by red and blue colors, respectively) based on the optimized S_0 geometries and the SOCME values of (a) **DiKTa**, (b) **DiKTaSe** and (c) **tBuCz-DiKTaSe** based on the optimized T_1 geometries.

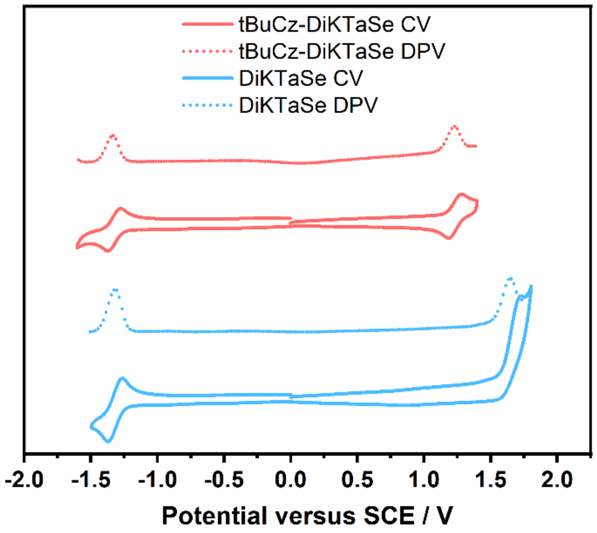

Figure **S28**. CV and DPV of **DiKTaSe** and **tBuCz-DiKTaSe** in DCM in degassed DCM with 0.1 M [^{n}Bu$_4$N]PF$_6$ as the supporting electrolyte and Fc/Fc$^+$ as the internal reference versus SCE (0.46 V vs. SCE)[16]. CV was performed at a sweep rate of 100 mV/s. DPV was conducted with an increment potential of 0.01 V and a pulse amplitude, width, and period of 50 mV, 0.06, and 0.5 s, respectively.

Table **S2**. Electrochemical data of **DiKTaSe** and **tBuCz-DiKTaSe**.[a]

	E_{ox} / V	E_{red} / V	HOMO/ eV	LUMO/ eV	ΔE / eV
DiKTaSe	1.18	-1.78	-5.98	-3.02	2.96
tBuCz-DiKTaSe	0.77	-1.79	-5.57	-3.01	2.56

[a.] In degassed DCM with 0.1 M [^{n}Bu$_4$N]PF$_6$ as the supporting electrolyte and Fc/Fc$^+$ as the internal reference (0.46 V vs. SCE)[16]. The HOMO and LUMO energies were determined using the relation HOMO/LUMO = -(E_{ox} / E_{red} + 4.8) eV,[17b] where E_{ox} and E_{red} are the peak of anodic and cathodic potentials from DPV relative to Fc/Fc$^+$. ΔE is the energy gap between HOMO and LUMO.

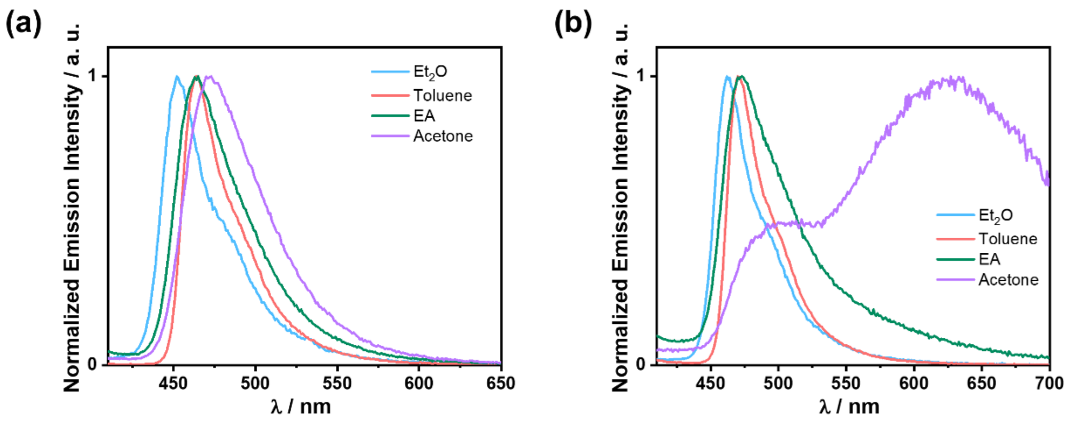

Figure **S29**. PL spectra of (a) **DiKTaSe** and (b) **tBuCz-DiKTaSe** in different solvents at 300K. (λ_{exc} = 340 nm)

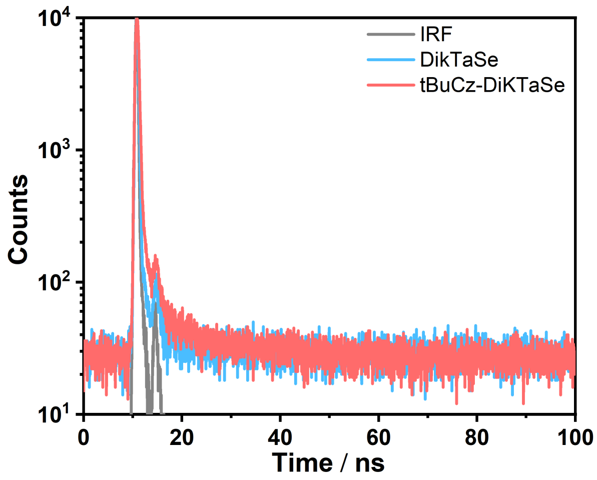

Figure **S30**. Time-resolved PL decays of the prompt emission (time window 0-100 ns) for **DiKTaSe** and **tBuCz-DiKTaSe** in degassed toluene at 300 K (λ_{exc} = 375 nm).

Table **S3**. Φ_{PL} values of **DiKTaSe** and **tBuCz-DiKTaSe** in 26-DCzPPy films (λ_{exc} = 430 nm).

Φ_{PL} (λ_{PL}) / % (nm)	1 wt% in 26-DCzPPy	2 wt% in 26-DCzPPy	5 wt% in 26-DCzPPy	10 wt% in 26-DCzPPy
**DiKTaSe**	69 (477)	62 (484)	53 (490)	44 (494)
**tBuCz-DiKTaSe**	88 (481)	82 (489)	69 (491)	62 (496)

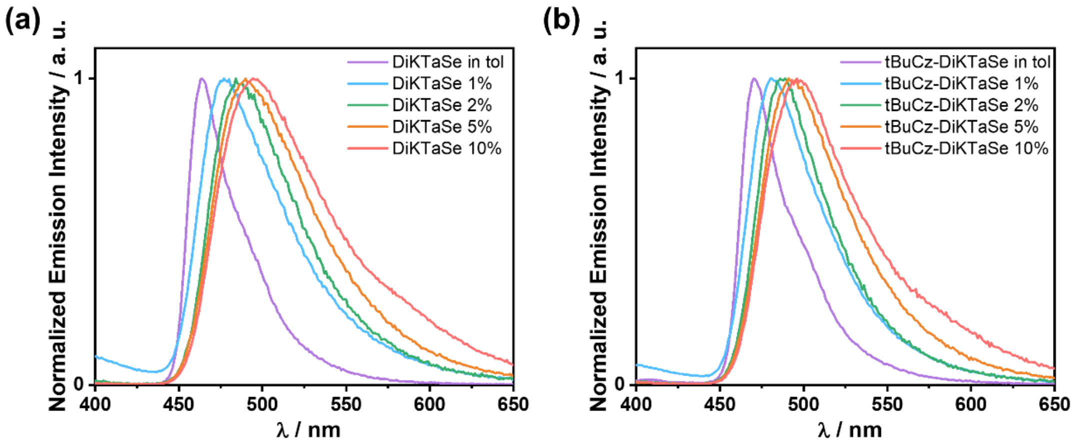

Figure **S31**. Steady-state PL spectra of (a) **DiKTaSe** and (b) **tBuCz-DiKTaSe** at 300 K in toluene and different doping concentration films in 26-DCzPPy (λ_{exc} = 340 nm).

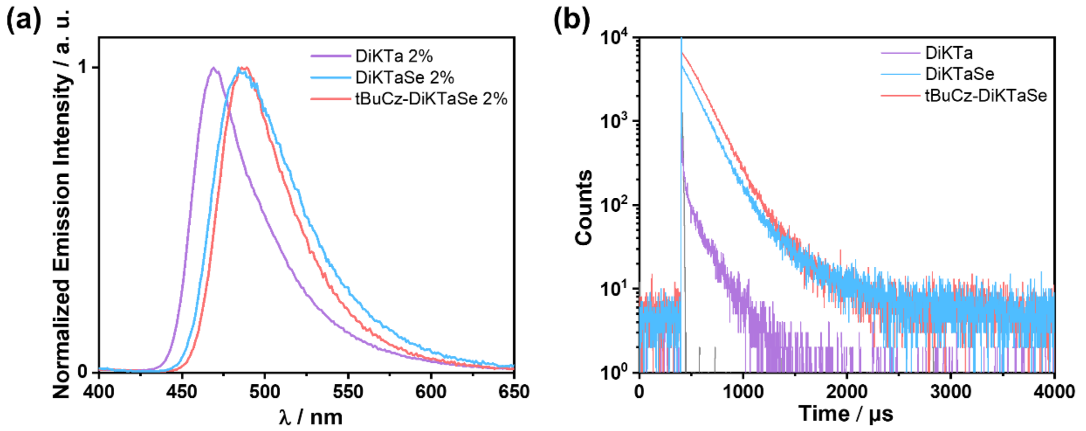

Figure **S32**. (a) Steady-state PL spectra at 300 K of 2 wt% doped film of **DiKTa**, **DiKTaSe**, and **tBuCz-DiKTaSe** in 2,6-DCzPPy (λ_{exc} = 340 nm). (b) Time-resolved PL decays of 2 wt% doped film of **DiKTa**, **DiKTaSe**, and **tBuCz-DiKTaSe** in 2,6-DCzPPy (λ_{exc} = 375 nm).

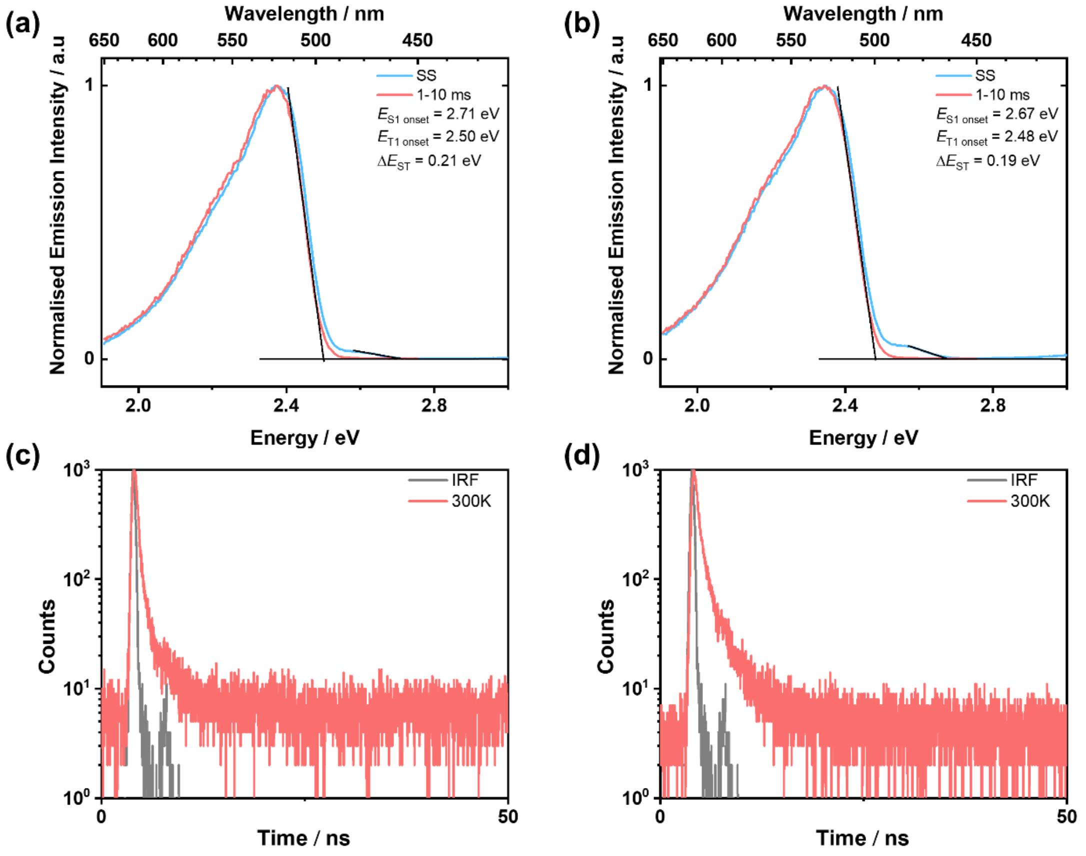

Figure **S33**. Steady state PL spectra and phosphorescence spectra of (a) **DiKTaSe** and (b) **tBuCz-DiKTaSe** in 2 wt% doped films in 26-DCzPPy at 77 K (λ_{exc} = 340 nm). PL decays of the prompt components of (c) **DiKTaSe** and (d) **tBuCz-DiKTaSe** in 2 wt% doped films in 26-DCzPPy at 300 K (λ_{exc} = 375 nm).

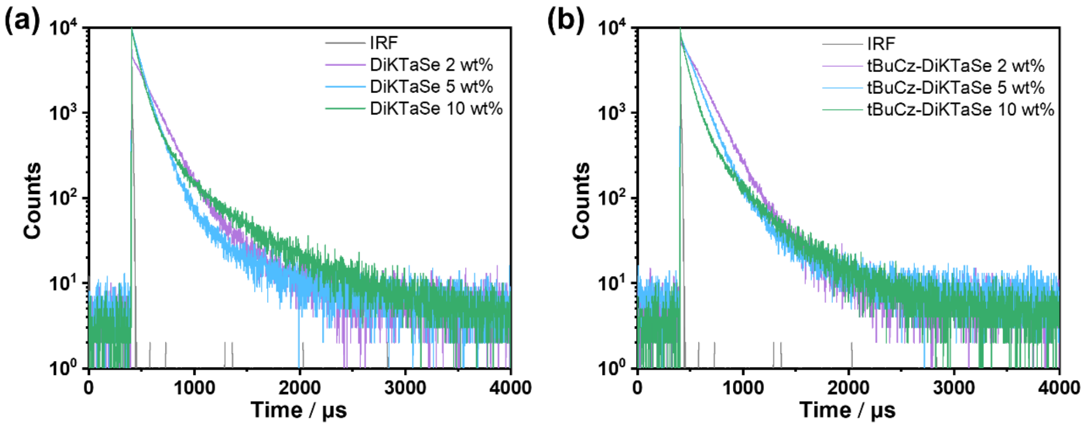

Figure **S34**. Time-resolved PL decays of 2, 5, and 10 wt% doped film of (a) **DiKTaSe** and (b) **tBuCz-DiKTaSe** in 2,6-DCzPPy (λ_{exc} = 375 nm).

The kinetics parameters were calculated according to the following equations and summarized in Table **S4**.[26]

$$\Phi_{PL} = \Phi_p + \Phi_d \quad \text{(S1)}$$

$$k_P = \frac{1}{\tau_p} \quad \text{(S2)}$$

$$k_d = \frac{1}{\tau_d} \quad \text{(S3)}$$

$$k_r^S = k_p \Phi_p \quad \text{(S4)}$$

$$k_{ISC} = k_p(1 - \Phi_p) \quad \text{(S5)}$$

$$k_{RISC} = \frac{k_p k_d}{k_{ISC}} \frac{\Phi_d}{\Phi_p} \quad \text{(S6)}$$

Where the Φ_p and Φ_d are the prompt fluorescent and delayed fluorescent quantum efficiency, which are calculated by integrating the transient PL curves; k_p is the rate constant of prompt fluorescence; k_d is the rate constant of delayed fluorescence; k_r^S is the radiative decay rate constant of S_1; k_{ISC} is the intersystem crossing rate constant; k_{RISC} is the reverse intersystem crossing rate constant.

Table **S4**. Summary of kinetics parameters of 2 wt% **DiKTa**, **DiKTaSe** and **tBuCz-DiKTaSe** doped films in 26-DCzPPy.

	Φ_p /%	Φ_d /%	k_p / 10^8 s^{-1}	k_d / 10^3 s^{-1}	k_r^S / 10^7 s^{-1}	k_{ISC} / 10^9 s^{-1}	k_{RISC} / 10^4 s^{-1}
**DiKTa**	30	40	2.60	6.58	7.81	0.18	1.25
**DiKTaSe**	3	59	17.1	5.08	5.31	1.66	9.97
**tBuCz-DiKTaSe**	2	80	9.17	4.99	2.26	0.90	16.5

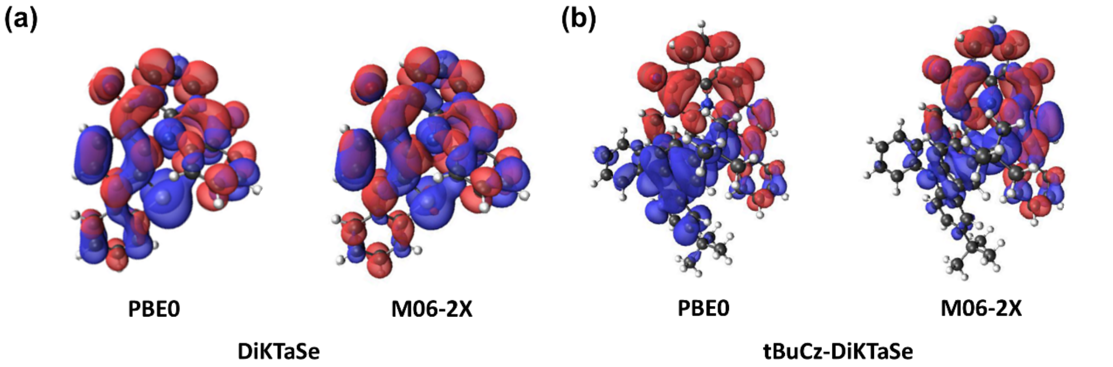

Figure **S35**. Density plot of the NTO hole (blue) & electron (red) of the S_1 states of (a) **DiKTaSe** and (b) **tBuCz-DiKTaSe** calculated at PBE0/6-31G(d,p) and M06-2X/6-31G(d,p), plotted with isovalue: 0.02.

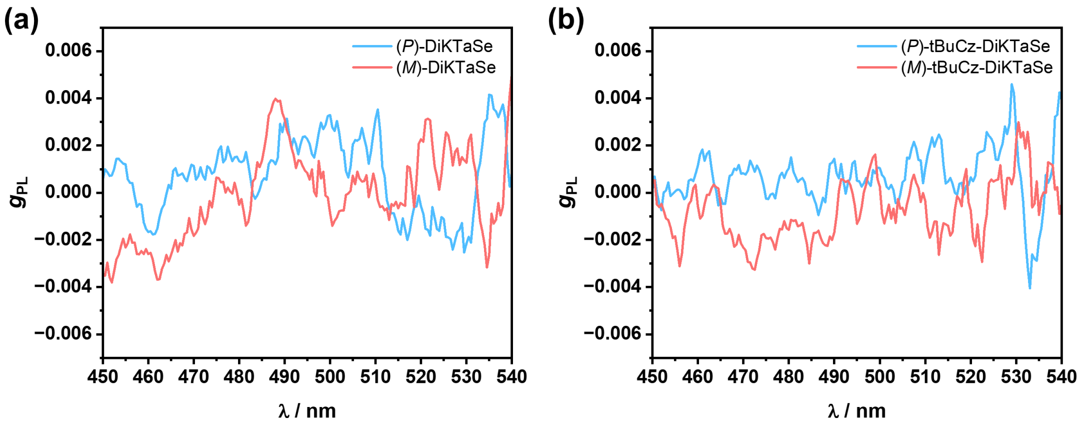

Figure **S36**. g_{PL} values of **DiKTaSe** and **tBuCz-DiKTaSe** recorded in 10^{-5} mol L^{-1} toluene solutions.

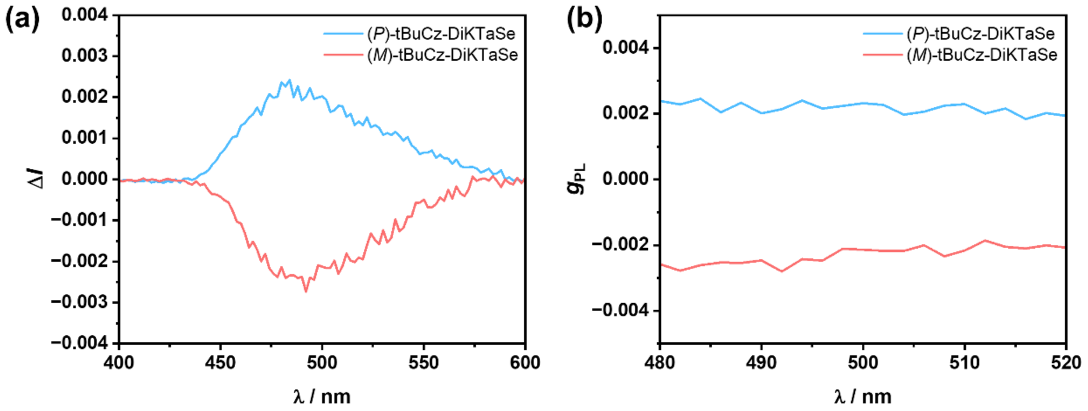

Figure **S37**. Solid-state CPL measurements of **tBuCz-DiKTaSe** enantiomers (2 wt% in PMMA). (λ_{exc} = 328 nm).

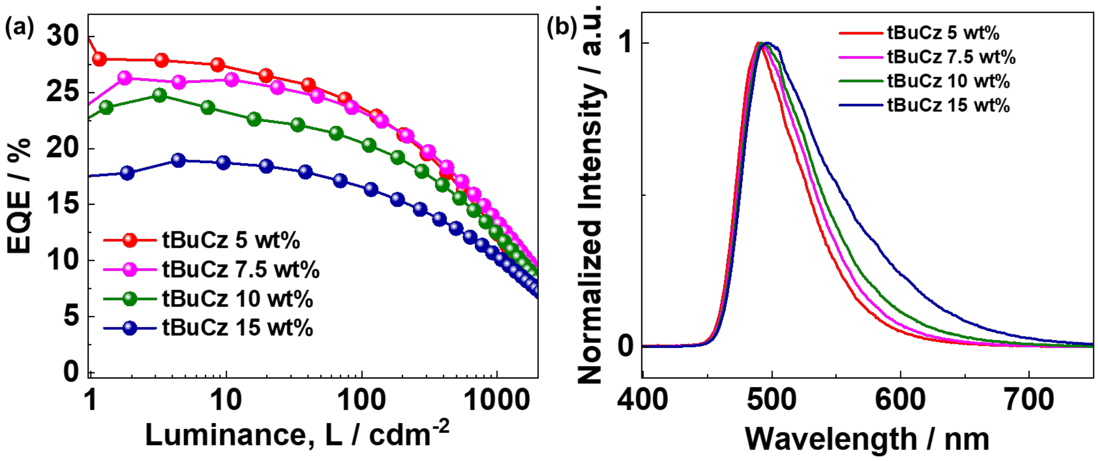

Figure **S38**. (a) EQE vs Luminance of the OLEDs with higher concentration 7.5, 10, and 15 wt% compared with 5 wt% using **tBuCz-DiKTaSe** as an emitter. (b) Electroluminescence spectra for the corresponding OLEDs.

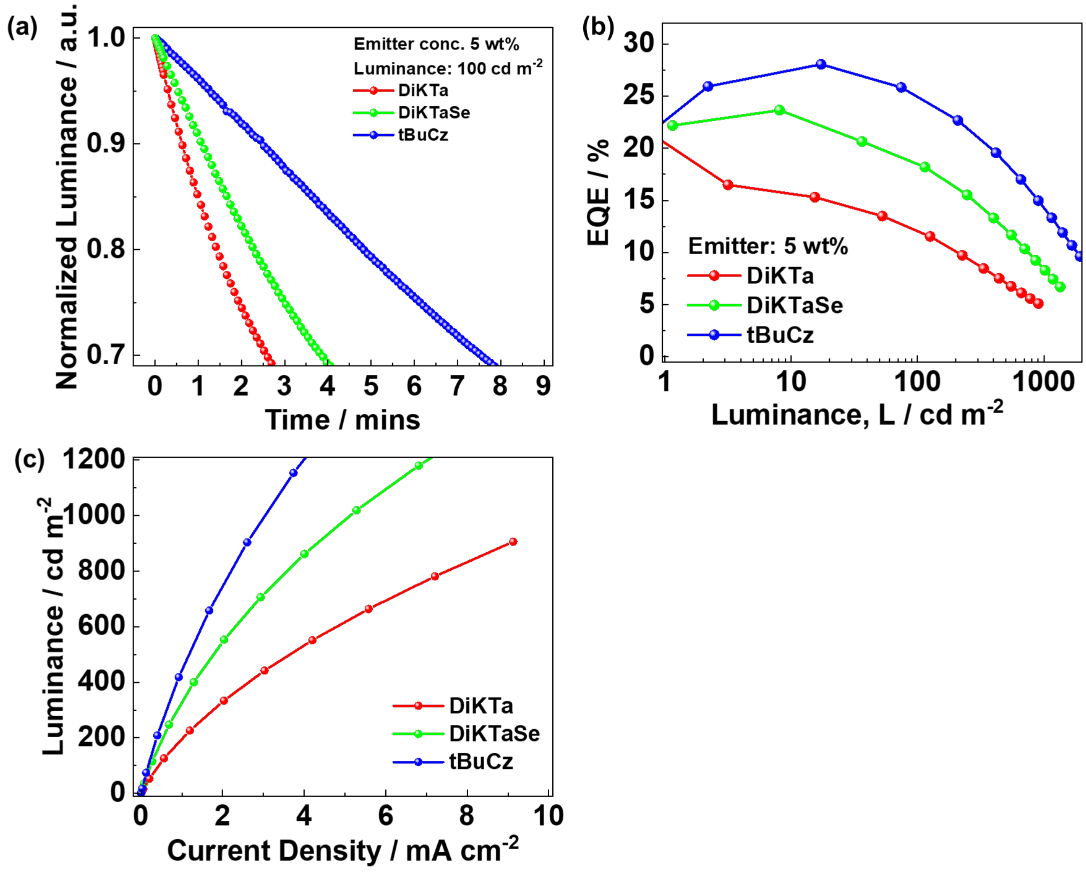

Figure **S39**. (a) Operational stability of the OLEDs with Se-based emitters compared with **DiKTa**. All devices were made using 5 wt% of the emitter concentration with similar device structures. Lifetimes were measured at the current density required for each device to start with a luminance of 100 cd m^{-2}. (b) EQE vs luminance for OLEDs with **DiKTa** and Se-based emitters. (c) luminance vs current density for the OLEDs with **DiKTa** and Se-based emitters.

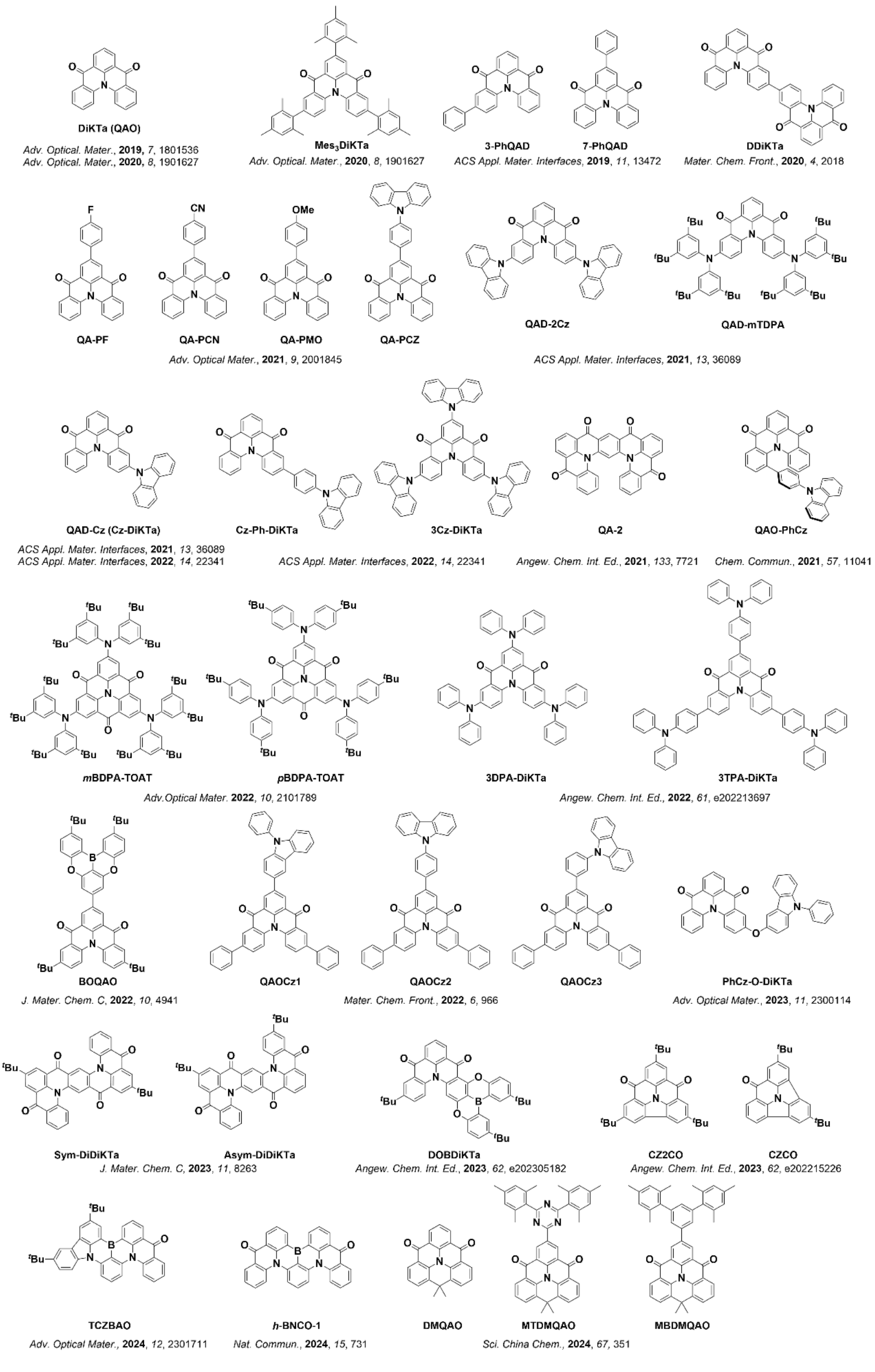
DiKTa (QAO)
Adv. Optical. Mater., 2019, 7, 1801536
Adv. Optical. Mater., 2020, 8, 1901627
Mes3DiKTa
Adv. Optical. Mater., 2020, 8, 1901627
3-PhQAD
7-PhQAD
ACS Appl. Mater. Interfaces, 2019, 11, 13472
DDiKTa
Mater. Chem. Front., 2020, 4, 2018
F
CN
OMe
QA-PF
QA-PCN
QA-PMO
QA-PCZ
Adv. Optical Mater., 2021, 9, 2001845
QAD-2Cz
QAD-mTDPA
ACS Appl. Mater. Interfaces, 2021, 13, 36089
QAD-Cz (Cz-DiKTa)
ACS Appl. Mater. Interfaces, 2021, 13, 36089
ACS Appl. Mater. Interfaces, 2022, 14, 22341
Cz-Ph-DiKTa
3Cz-DiKTa
ACS Appl. Mater. Interfaces, 2022, 14, 22341
QA-2
Angew. Chem. Int. Ed., 2021, 133, 7721
QAO-PhCz
Chem. Commun., 2021, 57, 11041
mBDPA-TOAT
pBDPA-TOAT
Adv.Optical Mater. 2022, 10, 2101789
3DPA-DiKTa
3TPA-DiKTa
Angew. Chem. Int. Ed., 2022, 61, e202213697
BOQAO
J. Mater. Chem. C, 2022, 10, 4941
QAOCz1
QAOCz2
QAOCz3
Mater. Chem. Front., 2022, 6, 966
PhCz-O-DiKTa
Adv. Optical Mater., 2023, 11, 2300114
Sym-DiDiKTa
Asym-DiDiKTa
J. Mater. Chem. C, 2023, 11, 8263
DOBDiKTa
Angew. Chem. Int. Ed., 2023, 62, e202305182
CZ2CO
CZCO
Angew. Chem. Int. Ed., 2023, 62, e202215226
TCZBAO
Adv. Optical Mater., 2024, 12, 2301711
h-BNCO-1
Nat. Commun., 2024, 15, 731
DMQAO
MTDMQAO
MBDMQAO
Sci. China Chem., 2024, 67, 351

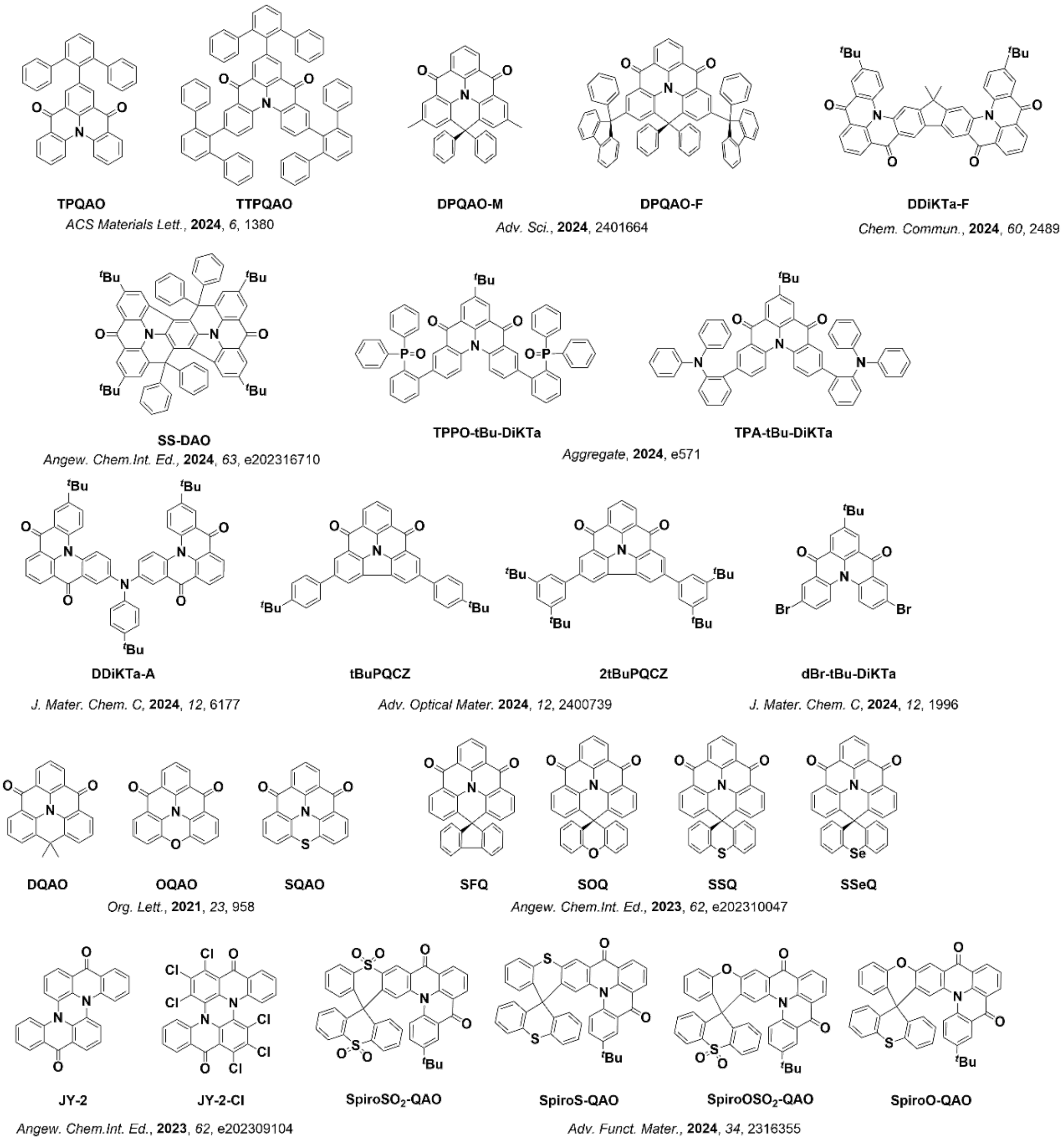

Figure **S40**. The structures of derivatives of **DiKTa** MR-TADF emitters.

Table **S5**. Properties of derivatives of **DiKTa** MR-TADF emitters in OLEDs.

Emitters	ΔE_{ST} / eV	λ_{EL} / nm	FWHM / nm	EQE_{max} / %	EQE_{100} / %	EQE_{1000} / %	Ref.
DiKTa	0.20 [a]	465	39	14.7	8.3	3.3	[27]
Mes_3DiKTa	0.21 [a]	480	36	21.1	14.5	4.5	
QAO (DiKTa)	0.18 [b]	468	39	19.4	-	-	[28]
3Ph-QAD	0.18 [c]	480	44	19.1	11.0	-	[29]

7Ph-QAD	0.19 [c]	472	34	18.7	5.8	-	
DDiKTa	0.16 [d]	500	59	19.0	-	-	[30]
QA-PF	0.24 [e]	474	27	16.8	5.6	-	[31]
QA-PCN	0.18 [e]	473	30	16.9	9.4	-	
QA-PMO	0.25 [e]	484	27	15.0	3.5	-	
QA-PCZ	0.21 [e]	482	29	17.5	7.6	-	
QAD-Cz	0.20 [f]	494	57	20.3	5.4	0.73	[32]
QAD-2Cz	0.16 [f]	530	56	27.3	23.9	12.4	
QAD-mTDPA	0.17 [f]	589	67	26.3	12.9	4.7	
QA-2	0.19 [g]	463	37	19.0	-	-	[33]
Cz-DiKTa	0.14 [c]	511	62	24.9	20.4	13.0	[34]
Cz-Ph-DiKTa	0.10 [c]	492	61	23.0	19.3	10.2	
3Cz-DiKTa	0.16 [c]	547	54	24.4	17.3	6.2	
QAO-PhCz	0.11 [f]	467	36	14.0	-	-	[35]
BOQAO	0.22 [f]	484	32	21.8	-	-	[36]
*m*BDPA-TOAT	0.23 [f]	600	45	17.3	-	-	[37]
*p*BDPA-TOAT	0.23 [f]	624	62	11.3	-	-	
QAOCz1	0.26 [f]	516	44	16.9	-	-	[38]
QAOCz2	0.18 [f]	504	43	19.4	-	-	
QAOCz3	0.16 [f]	500	40	21.1	-	-	
3TPA-DiKTa	0.20 [c]	551	62	30.8	18.1	7.3	[39]
3DPA-DiKTa	0.13 [c]	613	60	16.7	3.4	1.9	
PhCz-O-DiKTa	0.18 [h]	497	58	20	9.1	2.9	[40]
Sym-DiDiKTa	0.24 [f]	543	36	9.8	1.8	0.8	[41]
Asym-DiDiKTa	0.23 [f]	544	36	10.5	1.3	0.8	
DOBDiKTa	0.20 [f]	458	38	17.4	11.8	5.5	[42]
CZCO	0.33 [f]	432	35	15.6	6.0	5.9	[43]
CZ2CO	0.37 [f]	445	23	13	9.9	3.9	

TCZBAO	0.10[f]	520	36	25.1	21.7	14.0	[44]
h-BNCO-1	0.03[f]	528	39	40.1	-	34.6	[45]
DMQAO	0.21[f]	472	33	15.2	8.5	-	[46]
MTDMQAO	0.20[f]	472	39	29.4	21.1	-	
MBDMQAO	0.24[f]	480	27	18.9	4.7	-	
TPQAO	0.24[f]	468	31	18.1	-	-	[47]
TTPQAO	0.27[f]	480	33	21.3	-	-	
DPQAO-M	0.25[f]	471	27	16.6	4.6	2.2	[48]
DPQAO-F	0.24[f]	463	24	19.9	8.3	3.0	
SS-DAO	0.18[f]	520	24	37.2	24.8	7.1	[49]
DDiKTa-A	0.16[c]	572	69	20.3	13.2	3.9	[50]
*t*BuPQCZ	0.23[i]	470	42	20.4	6.4	-	[51]
2*t*BuPQCZ	0.24[i]	464	36	21.5	5.4	-	
DDiKTa-F	0.20[j]	493	46	15.3	9.9	-	[52]
TPPO-tBu-DiKTa	0.20[j]	480	46	24.4	10.0	3.7	[53]
TPA-tBu-DiKTa	0.19[j]	503	77	31.0	21.8	11.0	
DQAO	0.19[f]	472	34	15.2	8.5	-	[54]
OQAO	0.16[f]	532	45	20.3	15.1	-	
SQAO	0.16[f]	564	72	17.8	13.6	-	
JY-2	0.29[f]	524	-	18.1	-	-	[55]
JY-2-Cl	0.24[f]	546	-	29.1	-	-	
dBr-tBu-DiKTa	0.21[k]	480	54	21.2	11.4	1.95	[56]
SFQ	0.23[f]	460	30	21.7	12.5	3.6	[57]
SOQ	0.24[f]	456	33	24.3	14.9	4.2	
SSQ	0.23[f]	456	31	25.5	13.4	3.5	
SSeQ	0.24[f]	460	35	22.2	13.0	3.1	
SpiroS-QAO	0.21[f]	507	44	27.3	-	-	[58]
SpiroSO2-QAO	0.21[f]	470	46	9.0	-	-	

SpiroO-QAO	0.23[f]	487	34	18.0	-	-	
SpiroOSO2-QAO	0.23[f]	492	39	18.5	-	-	
**DiKTaSe**	0.22[j]	480	63	23.2	15.8	6.7	**This work**
**tBuCz-DiKTaSe**	0.22[j]	490	57	28.0	23.6	12.4	

[a.] 3.5 wt% doped films in mCP; [b] 5 wt% doped films in mCP; [c] 2 wt% doped films in mCP; [d] 9 wt% doped films in DPEPO; [e] 3 wt% doped films in mCP; [f] In toluene; [g] 3 wt% doped films in PPCz; [h] 10 wt% doped films in mCP; [i] 5 wt% doped films in mCBP; [j] In 2-MeTHF; [k] 1 wt% doped films in mCP.

References:

[1] M. J. Frisch, G. W. Trucks, H. B. Schlegel, G. E. Scuseria, M. A. Robb, J. R. Cheeseman, G. Scalmani, V. Barone, G. A. Petersson, H. Nakatsuji, X. Li, M. Caricato, A. V. Marenich, J. Bloino, B. G. Janesko, R. Gomperts, B. Mennucci, H. P. Hratchian, J. V. Ortiz, A. F. Izmaylov, J. L. Sonnenberg, Williams, F. Ding, F. Lipparini, F. Egidi, J. Goings, B. Peng, A. Petrone, T. Henderson, D. Ranasinghe, V. G. Zakrzewski, J. Gao, N. Rega, G. Zheng, W. Liang, M. Hada, M. Ehara, K. Toyota, R. Fukuda, J. Hasegawa, M. Ishida, T. Nakajima, Y. Honda, O. Kitao, H. Nakai, T. Vreven, K. Throssell, J. A. Montgomery Jr., J. E. Peralta, F. Ogliaro, M. J. Bearpark, J. J. Heyd, E. N. Brothers, K. N. Kudin, V. N. Staroverov, T. A. Keith, R. Kobayashi, J. Normand, K. Raghavachari, A. P. Rendell, J. C. Burant, S. S. Iyengar, J. Tomasi, M. Cossi, J. M. Millam, M. Klene, C. Adamo, R. Cammi, J. W. Ochterski, R. L. Martin, K. Morokuma, O. Farkas, J. B. Foresman, D. J. Fox, *Gaussian 16 Rev. C.01*, Wallingford, CT, 2016.

[2] C. Adamo, V. Barone, *J. Chem. Phys.* **1999**, *110*, 6158-6170.

[3] T. H. Dunning, Jr., *J. Chem. Phys.* **1989**, *90*, 1007-1023.

[4] a) S. Grimme, *Chem. Phys. Lett.* **1996**, *259*, 128-137; b) S. Hirata, M. Head-Gordon, *Chem. Phys. Lett.* **1999**, *314*, 291-299.

[5] R. Dennington, T. Keith, J. Millam, *GaussView, Version 6*, Semichem Inc., Shawnee Mission KS, 2019.

[6] a) C. Hättig, *J. Chem. Phys.* **2003**, *118*, 7751-7761; b) A. Hellweg, S. A. Grün, C.

Hättig, *Phys. Chem. Chem. Phys.* **2008**, *10*, 4119-4127.

[7] K. Momma, F. Izumi, *J. Appl. Crystallogr.* **2011**, *44*, 1272-1276.

[8] O. S. Lee, E. Zysman-Colman, *Silico (version 3.1), In-Silico-Computing*, St Andrews, Scotland, **2023**.

[9] N. M. O'Boyle, A. L. Tenderholt, K. M. Langner, *J. Comput. Chem.* **2008**, *29*, 839-845.

[10] W. Humphrey, A. Dalke, K. Schulten, *J. Mol. Graph. Model.* **1996**, *14*, 33-38.

[11] J. E. Stone, *An efficient library for parallel ray tracing and animation*, **1998**.

[12] J. D. Hunter, *Comput. Sci. Eng.* **2007**, *9*, 90-95.

[13] N. M. O'Boyle, M. Banck, C. A. James, C. Morley, T. Vandermeersch, G. R. Hutchison, *J. Cheminformatics* **2011**, *3*, 1-14.

[14] N. M. O'Boyle, C. Morley, G. R. Hutchison, *Chem. Cent. J.* **2008**, *2*, 1-7.

[15] X. Gao, S. Bai, D. Fazzi, T. Niehaus, M. Barbatti, W. Thiel, *J. Chem. Theory Comput.* **2017**, *13*, 515-524.

[16] N. G. Connelly, W. E. Geiger, *Chem. Rev.* **1996**, *96*, 877-910.

[17] a) J. Pommerehne, H. Vestweber, W. Guss, R. F. Mahrt, H. Bässler, M. Porsch, J. Daub, *Adv. Mater.* **1995**, *7*, 551-554; b) C. M. Cardona, W. Li, A. E. Kaifer, D. Stockdale, G. C. Bazan, *Adv. Mater.* **2011**, *23*, 2367-2371.

[18] G. A. Crosby, J. N. Demas, *J. Phys. Chem.* **1971**, *75*, 991-1024.

[19] W. H. Melhuish, *J. Phys. Chem.* **1961**, *65*, 229-235.

[20] N. Greenham, I. Samuel, G. Hayes, R. Phillips, Y. Kessener, S. Moratti, A. Holmes, R. Friend, *Chem. Phys. Lett.* **1995**, *241*, 89-96.

[21] *CrysAlisPro v1.171.41.93a and v1.171.43.109a*, Rigaku Oxford Diffraction, Rigaku Corporation, Tokyo, Japan, **2020-2023**.

[22] G. M. Sheldrick, *Acta Crystallogr., Sect. A: Found. Adv*. **2015**, *71*, 3-8.

[23] G. M. Sheldrick, *Acta Crystallogr., Sect. C: Struct. Chem.* **2015**, *71*, 3-8.

[24] A. L. Spek, *Acta Crystallogr., Sect D: Biol. Crystallogr.* **2009**, *65*, 148-155.

[25] O. V. Dolomanov, L. J. Bourhis, R. J. Gildea, J. A. Howard, H. Puschmann, *J. Appl. Crystallogr.* **2009**, *42*, 339-341.

[26] a) K. Masui, H. Nakanotani, C. Adachi, *Org. Electron.* **2013**, *14*, 2721-2726; b) Y.

Tsuchiya, S. Diesing, F. Bencheikh, Y. Wada, P. L. Dos Santos, H. Kaji, E. Zysman-Colman, I. D. W. Samuel, C. Adachi, *J. Phys. Chem. A* **2021**, *125*, 8074-8089.

[27] D. Hall, S. M. Suresh, P. L. dos Santos, E. Duda, S. Bagnich, A. Pershin, P. Rajamalli, D. B. Cordes, A. M. Z. Slawin, D. Beljonne, A. Köhler, I. D. W. Samuel, Y. Olivier, E. Zysman-Colman, *Adv. Optical Mater.* **2020**, *8*, 1901627.

[28] Y. Yuan, X. Tang, X. Y. Du, Y. Hu, Y. J. Yu, Z. Q. Jiang, L. S. Liao, S. T. Lee, *Adv. Optical Mater.* **2019**, *7*, 1801536.

[29] X. Li, Y. Z. Shi, K. Wang, M. Zhang, C. J. Zheng, D. M. Sun, G. L. Dai, X. C. Fan, D. Q. Wang, W. Liu, Y. Q. Li, J. Yu, X. M. Ou, C. Adachi, X. H. Zhang, *ACS Appl. Mater. Interfaces* **2019**, *11*, 13472-13480.

[30] D. Sun, S. M. Suresh, D. Hall, M. Zhang, C. Si, D. B. Cordes, A. M. Z. Slawin, Y. Olivier, X. Zhang, E. Zysman-Colman, *Mater. Chem. Front.* **2020**, *4*, 2018-2022.

[31] X. Qiu, G. Tian, C. Lin, Y. Pan, X. Ye, B. Wang, D. Ma, D. Hu, Y. Luo, Y. Ma, *Adv. Optical Mater.* **2021**, *9*, 2001845.

[32] F. Huang, K. Wang, Y. Z. Shi, X. C. Fan, X. Zhang, J. Yu, C. S. Lee, X. H. Zhang, *ACS Appl. Mater. Interfaces* **2021**, *13*, 36089-36097.

[33] H. Min, I. S. Park, T. Yasuda, *Angew. Chem. Int. Ed.* **2021**, *60*, 7643-7648.

[34] S. Wu, W. Li, K. Yoshida, D. Hall, S. Madayanad Suresh, T. Sayner, J. Gong, D. Beljonne, Y. Olivier, I. D. W. Samuel, E. Zysman-Colman, *ACS Appl. Mater. Interfaces* **2022**, *14*, 22341-22352.

[35] S. Y. Yang, S. N. Zou, F. C. Kong, X. J. Liao, Y. K. Qu, Z. Q. Feng, Y. X. Zheng, Z. Q. Jiang, L. S. Liao, *Chem. Commun.* **2021**, *57*, 11041-11044.

[36] Y.-J. Yu, S.-N. Zou, C.-C. Peng, Z.-Q. Feng, Y.-K. Qu, S.-Y. Yang, Z.-Q. Jiang, L.-S. Liao, *J. Mater. Chem. C* **2022**, *10*, 4941-4946.

[37] X. C. Fan, K. Wang, Y. Z. Shi, J. X. Chen, F. Huang, H. Wang, Y. N. Hu, Y. Tsuchiya, X. M. Ou, J. Yu, C. Adachi, X. H. Zhang, *Adv. Optical Mater.* **2022**, *10*, 2101789.

[38] J.-F. Liu, S.-N. Zou, X. Chen, S.-Y. Yang, Y.-J. Yu, M.-K. Fung, Z.-Q. Jiang, L.-S. Liao, *Mater. Chem. Front.* **2022**, *6*, 966-972.

[39] S. Wu, A. Kumar Gupta, K. Yoshida, J. Gong, D. Hall, D. B. Cordes, A. M. Z. Slawin,

I. D. W. Samuel, E. Zysman-Colman, *Angew. Chem. Int. Ed.* **2022**, *61*, e202213697.

[40] T. Wang, A. K. Gupta, D. B. Cordes, A. M. Z. Slawin, E. Zysman‐Colman, *Adv. Optical Mater.* **2023**, *11*, 2300114.

[41] J. M. dos Santos, C.-Y. Chan, S. Tang, D. Hall, T. Matulaitis, D. B. Cordes, A. M. Z. Slawin, Y. Tsuchiya, L. Edman, C. Adachi, Y. Olivier, E. Zysman-Colman, *J. Mater. Chem. C* **2023**, *11*, 8263-8273.

[42] S. Wu, L. Zhang, J. Wang, A. Kumar Gupta, I. D. W. Samuel, E. Zysman-Colman, *Angew. Chem. Int. Ed.* **2023**, *62*, e202305182.

[43] C. Cao, J. H. Tan, Z. L. Zhu, J. D. Lin, H. J. Tan, H. Chen, Y. Yuan, M. K. Tse, W. C. Chen, C. S. Lee, *Angew. Chem. Int. Ed.* **2023**, *62*, e202215226.

[44] Z. Yang, G. X. Yang, S. Jiang, M. Li, W. Qiu, X. Peng, C. Shen, Y. Gan, K. Liu, D. Li, *Adv. Optical Mater.* **2024**, *12*, 2301711.

[45] Y. C. Cheng, X. Tang, K. Wang, X. Xiong, X. C. Fan, S. Luo, R. Walia, Y. Xie, T. Zhang, D. Zhang, J. Yu, X. K. Chen, C. Adachi, X. H. Zhang, *Nat. Commun.* **2024**, *15*, 731.

[46] L. Chen, J.-H. Cai, Y.-J. Yu, Y.-K. Qu, S.-Y. Yang, S.-N. Zou, R.-H. Liu, D.-Y. Zhou, L.-S. Liao, Z.-Q. Jiang, *Sci. China Chem.* **2024**, *67*, 351-359.

[47] F.-M. Liu, Z.-H. Qu, P. Zuo, Y.-J. Yu, M.-T. Li, L.-S. Liao, D.-Y. Zhou, Z.-Q. Jiang, *ACS Materials Lett.* **2024**, *6*, 1380-1387.

[48] J. R. Yu, H. J. Tan, X. Q. Gao, B. Wang, Z. Q. Long, J. L. Liu, Z. Z. Lin, X. Y. Li, Z. L. Zhu, J. X. Jian, Q. X. Tong, C. S. Lee, *Adv. Sci.* **2024**, *11*, 2401664.

[49] L. Liang, C. Qu, X. Fan, K. Ye, Y. Zhang, Z. Zhang, L. Duan, Y. Wang, *Angew. Chem. Int. Ed.* **2024**, *63*, e202316710.

[50] W. Sen, Y.-N. Hu, J. Wang, D. Sun, K. Wang, X.-H. Zhang, E. Zysman-Colman, *J. Mater. Chem. C* **2024**, *12*, 6177-6184.

[51] J. Xu, M. Wang, J. Chen, Z. Wu, T. Guo, B. Z. Tang, Z. Zhao, *Adv. Optical Mater.* **2024**, *12*, 2400739.

[52] S. Wu, Y. N. Hu, D. Sun, K. Wang, X. H. Zhang, E. Zysman-Colman, *Chem. Commun.* **2024**, *60*, 2489-2492.

[53] J. Wang, H. Hafeez, S. Tang, T. Matulaitis, L. Edman, I. D. W. Samuel, E. Zysman-Colman, *Aggregate* **2024**, *5*, e571.

[54] S. N. Zou, C. C. Peng, S. Y. Yang, Y. K. Qu, Y. J. Yu, X. Chen, Z. Q. Jiang, L. S. Liao, *Org. Lett.* **2021**, *23*, 958-962.

[55] Y. Jiao, Z. Chen, W. Qiu, H. Xie, J. Yang, X. Peng, W. Xie, Q. Gu, M. Li, K. Liu, S. J. Su, *Angew. Chem. Int. Ed.* **2023**, *62*, e202309104.

[56] H. Miranda-Salinas, J. Wang, A. Danos, T. Matulaitis, K. Stavrou, A. P. Monkman, E. Zysman-Colman, *J. Mater. Chem. C* **2024**, *12*, 1996-2006.

[57] Y. J. Yu, Z. Q. Feng, X. Y. Meng, L. Chen, F. M. Liu, S. Y. Yang, D. Y. Zhou, L. S. Liao, Z. Q. Jiang, *Angew. Chem. Int. Ed.* **2023**, *62*, e202310047.

[58] S. Jiang, D. Liu, Z. Chen, Z. Yang, Y. He, G. X. Yang, D. Li, S. J. Su, *Adv. Funct. Mater.* **2024**, *34*, 2316355.
